# Supplementary material for: Common fronto-temporal effective connectivity in humans and monkeys
Source: Neuron. 2021 Mar 3;109(5):852–868.e8. doi: 10.1016/j.neuron.2020.12.026 (PMC7927917; doi:10.1016/j.neuron.2020.12.026)
Supplement: Document S1. Tables S2–S4 and Figures S1–S6 [file mmc1.pdf]

Neuron, Volume *109*

Supplemental information

Common fronto-temporal effective connectivity in humans and monkeys

Francesca Rocchi, Hiroyuki Oya, Fabien Balezeau, Alexander J. Billig, Zsuzsanna Kocsis, Rick L. Jenison, Kirill V. Nourski, Christopher K. Kovach, Mitchell Steinschneider, Yukiko Kikuchi, Ariane E. Rhone, Brian J. Dlouhy, Hiroto Kawasaki, Ralph Adolphs, Jeremy D.W. Greenlee, Timothy D. Griffiths, Matthew A. Howard III, and Christopher I. Petkov

Table S2. Human esfMRI Results, Related to Figure 3. Shown are the human brain regions in reference to the human atlas in MNI x,y,z coordinates, the peak voxel value in the region and the number of voxels above the corrected significance threshold (cluster-wise alpha < 0.05 with primary *p* < 0.005).

Site 1 (medHG) esfMRI human group results:

		MNI coordinates (mm)			
**size (voxel)**	**peak T**	**x**	**y**	**z**	Brain structure
831	7.38	54.5	0.5	-28.5	lt. middle temporal gyrus
327	7.10	66.5	56.5	13.5	lt. superior temporal gyrus
291	5.77	-41.5	4.5	-12.5	rt. insula
241	5.62	-65.5	26.5	27.5	rt. supramarginal gyrus
166	4.97	42.5	-29.5	1.5	lt. inferior frontal gyrus, including frontal operculum and orbitofrontal cortex
149	6.54	2.5	26.5	-4.5	lt. thalamus
123	4.57	-32.5	-18.5	-2.5	rt. putamen
84	5.26	32.5	-47.5	21.5	lt. middle frontal gyrus

Site 2 (latHG + PT) esfMRI human group results:

		MNI coordinates (mm)			
**size (voxel)**	**peak T**	**x**	**y**	**z**	Brain structure
696	7.29	-23.5	-5.5	-26.5	rt. parahippocampal gyrus, including hippocampus and amygdala
600	7.77	46.5	2.5	-30.5	lt. inferior temporal gyrus
377	7.19	-63.5	10.5	37.5	rt. postcentral gyrus
173	7.34	58.5	-11.5	-6.5	lt. superior temporal gyrus
140	7.58	-31.5	64.5	65.5	rt. superior parietal lobule
104	4.68	-15.5	21.5	-17.5	lt. superior orbital gyrus

Site 1 versus Site 2 esfMRI human group results:

		MNI coordinates (mm)			
**size (voxel)**	**peak T**	**x**	**y**	**z**	Brain structure
430	7.21	66.5	56.5	13.5	lt. middle and superior temporal gyrus
196	7.17	-63.5	28.5	25.5	rt. supramarginal gyrus
106	5.39	48.5	-15.5	1.5	lt. inferior frontal gyrus, including frontal operculum and orbitofrontal cortex

Site 2 versus Site 1 esfMRI human group results:

**size (voxel)**	**peak T**	**MNI coordinates (mm)**			
		**x**	**y**	**z**	Brain structure
971	5.65	-35.5	-19.5	-28.5	rt. ATL (incl. entorhinal cortex, parahippocampal gyrus, temporal pole & amygdala)
283	5.60	22.5	-5.5	-28.5	lt. ATL (incl. entorhinal cortex & amygdala)
262	5.71	-63.5	6.5	21.5	lt. precentral gyrus

Table S3. Human Patient Demographics, Related to Figure 3

Patient ID	Age	Sex	Handedness	Language dominance (Wada test)	esfMRI	esT	Speech
292	50	F	L	L	Yes (Y)		
302	47	F	R	**	Y		
307	30	M	R	L	Y		
314	30	F	R	L	Y		
316	31	F	R	**	Y		
320	50	F	R	**	Y		
330	43	M	L	**	Y		
331	35	M	R	**	Y		
334	39	M	L	L	Y		
335	31	M	R	L	Y		
339	45	M	R	**	Y		
352	31	M	Mixed	**	Y		
357	36	M	R	L	Y		Y
369	30	M	R	L	Y	Y	Y
372	34	M	R	L	Y	Y	Y
376	48	F	R	L	Y	Y	Y
384	37	M	R	L		Y	
394	23	M	L	L		Y	Y
395	13	M	R	**	Y		
399	22	F	R	L	Y	Y	Y
400	59	M	L	L	Y		
403	56	F	R	L	Y		
405	19	M	R	L	Y	Y	Y
413	22	M	L	R	Y	Y	
418	25	F	R	L		Y	
423	49	M	R	L		Y	Y
427	17	M	Mixed	**		Y	
429	32	F	R	**		Y	Y
457	18	M	R	**			Y
460	52	M	R	L (fMRI)		Y	

**(not done)

Table S4. Human Patient Clinical Observations and Surgical Resection Sites, Related to Figure 3

Patient ID	Clinical MRI finding	Surgery	Seizure onset zone
292	Left frontal focal encephalomalacia	left ATL+ left frontal seizure focus resection	Left mesial temporal lobe + left frontal lobe
302	Left MTS	Left ATL	Left mesial temporal lobe
307	Left insular cavernoma	Cavernoma resection	Left insula
314	Left occipital cortical dysplasia	No resection	Bilateral mesial temporal lobe
316	Right MTS	Right ATL	Right mesial temporal lobe
320	Right MTS	Right ATL	Right hippocampus
330	Right occipital cortical dysplasia	Right occipital lesionectomy	Left occipital lobe
331	Right MTS	No resection	Left mesial temporal lobe
334	Right temporal ganglioglioma	Right ATL	Right temporal pole, ventral surface of temporal lobe
335	Left MTS	No resection	Bilateral medial temporal lobe
339	Normal	No resection	Not determined
352	Left frontal cystic legion	Left frontal regionectomy	Left frontal cystic mass
357	Normal	Left ATL	Left mesial temporal lobe
369	Right basal ganglia venous anomaly	Right ATL	Right mesial temporal lobe
372	Normal	Left ATL	Left temporal pole
376	Normal	Right ATL	Right mesial temporal lobe
384	Normal	Right ATL, Right frontal pole resection	Right mesial temporal lobe, Right frontal pole
394	Right temporal lobe cavernoma	Right ATL	Right amygdala
395	Left frontal lobe cavernoma	Left mesial frontal lobe resection including left frontal cavernoma	Left frontal lobe
399	Normal	Right ATL+ right ventral frontal seizure focus resection	Right mesial temporal lobe + possible right basal frontal lobe
400	Left MTS	Left ATL	Left mesial temporal lobe
403	Normal	Left ATL	Left mesial temporal lobe
405	Left frontal encephalomalacia	Left frontal regionectomy	Left frontal encephalomalacia
413	Normal	Right ATL	Right mesial temporal lobe
418	Right inferior temporal dysplasia	Right ATL	Right mesial temporal lobe
423	Normal	Left ATL	Left mesial temporal lobe
427	Right frontal encephalomalacia	Right frontal lobectomy	Right frontal lobe
429	Normal	Left ATL	Left mesial temporal lobe
457	Normal	Left ATL	Left mesial temporal lobe
460	Normal	Left ATL	Left mesial temporal lobe

MTS: Mesial temporal sclerosis　　ATL: Anterior temporal lobectomy

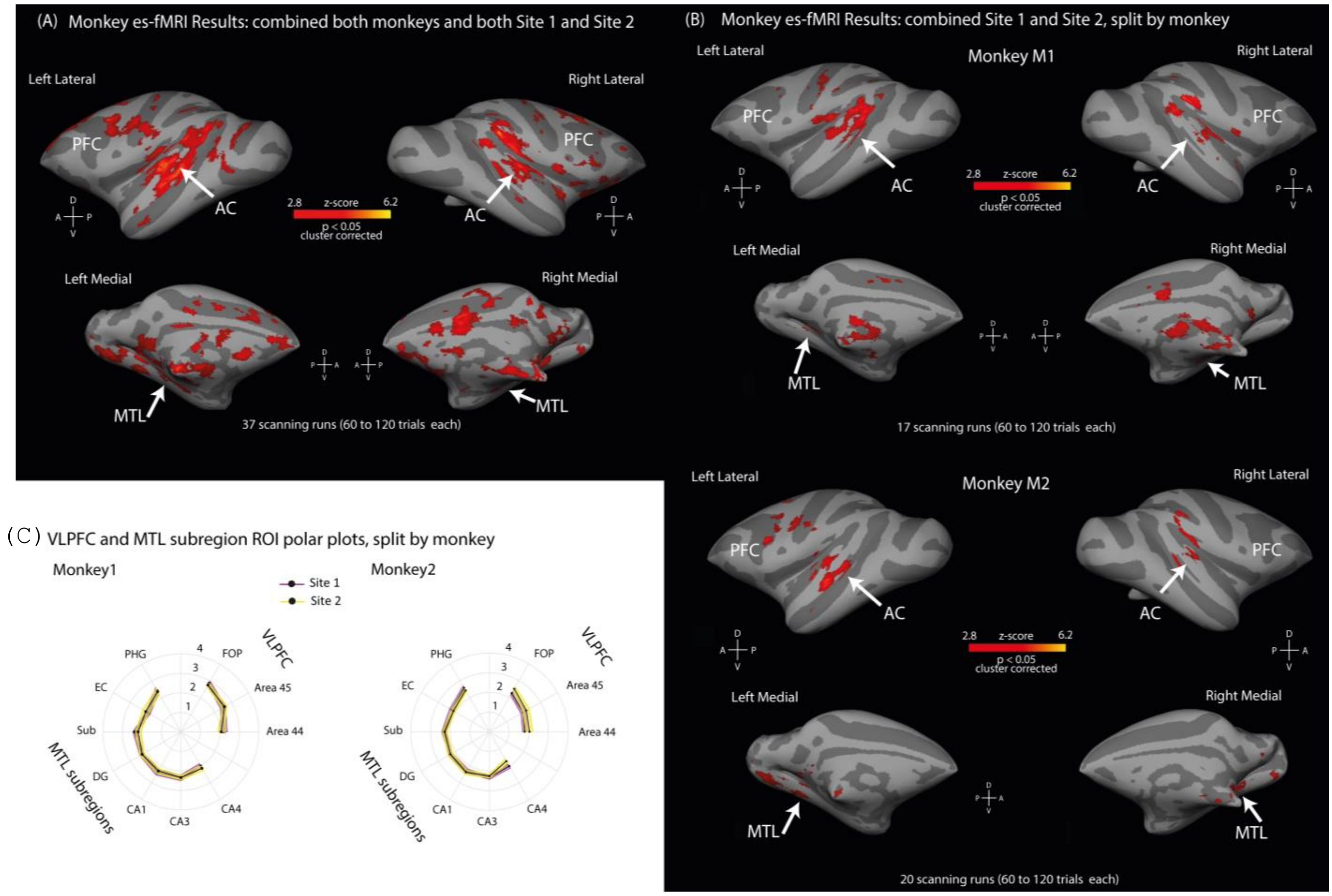

Supplementary Figure S1. Supplementary Monkey es-fMRI Whole Brain and ROI Results, Related to Figure 3. (A) Group es-fMRI result with both monkeys and both sites combined. Format and labels as in manuscript Fig. 2. (B) Group es-fMRI result combining both stimulation sites but shown split by monkey (M1 and M2). Format and labels as in manuscript Fig. 2. (C) ROI polar plot of the max *Z*-score (error boundaries: +/-SEM) across sessions/runs and participants for the VLPFC and MTL shown split by monkey (M1 and M2). Format and labels as in manuscript Fig. 5.

(A)

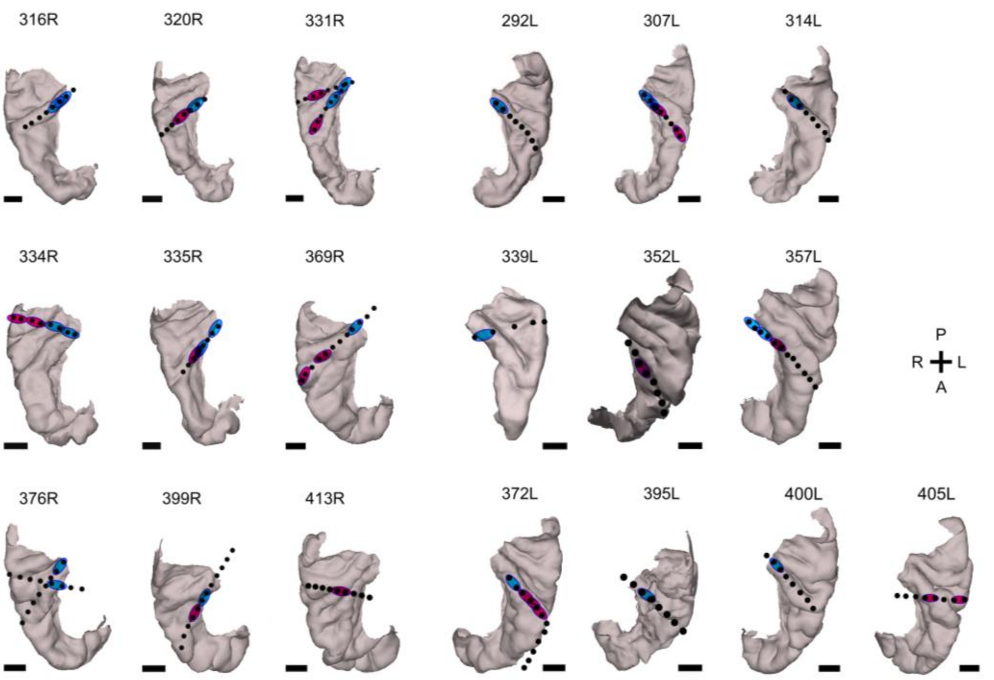
316R
320R
331R
292L
307L
314L
334R
335R
369R
339L
352L
357L
P
R
L
A
376R
399R
413R
372L
395L
400L
405L

(B)

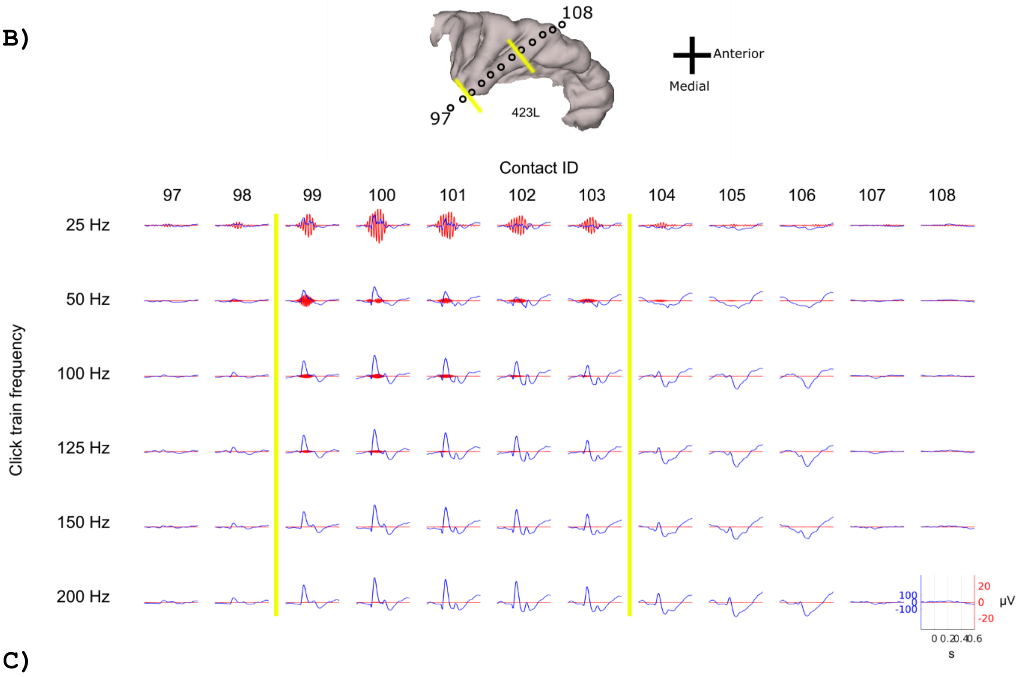
108
Anterior
Medial
97
423L
Contact ID
97
98
99
100
101
102
103
104
105
106
107
108
25 Hz
50 Hz
100 Hz
125 Hz
150 Hz
200 Hz
Click train frequency
100
-100
20
0
-20
μV
s

(C)

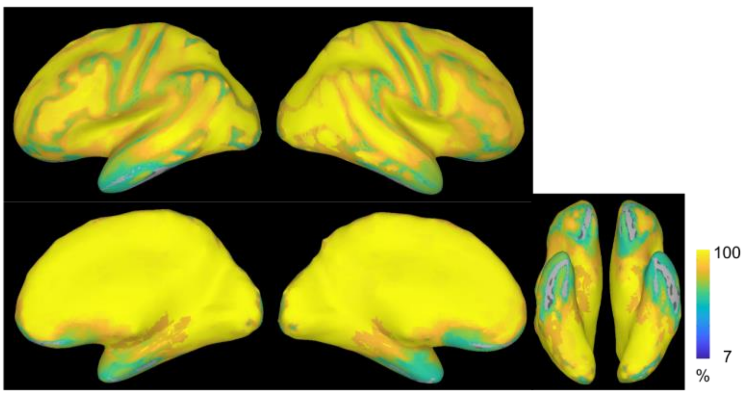
100
7
%

Supplementary Figure S2. Individual Human Depth Electrode Contact Locations for es-fMRI, Click Frequency Following Response Used to Identify medHG Sites, and Electrode Contact Locations Affecting fMRI Signal: Preserved fMRI Signal Map, Related to Figure 3.

(A) Shown are views looking down on the superior temporal plane. Black circles identify the clinically placed contacts and the blue/red regions the two contacts used for bipolar stimulation, respectively in the medHG or latHG+PT sites, shown on each individual's anatomy. Whenever more than two contacts are shown in red is an indication of other pairs of adjacent contacts that were stimulated in a separate testing run. Scale bars 10 mm. L = left; R = right; P = posterior; A = anterior.

(B) Click frequency following response used to identify Site 1 (medHG) sites in humans. Shown is how the click evoked neurophysiological response was used to identify medial HG contacts by their click-frequency following response. In this example, traces in blue show the auditory evoked potentials. Traces in red are the high-pass filtered frequency following response. Contacts between the two yellow lines show a strong frequency following response to 25-100Hz and were thus assigned to medHG. Contacts on the right are assigned to Site 2 (latHG+PT) because they respond to the sounds but do not show a clear high frequency following response. The non-responsive contacts at the flanks of these were not used.

(C) Human electrode contact locations affecting fMRI signal: preserved fMRI signal map. Regions where the fMRI signal was contaminated by the electrodes were masked and excluded from analysis. We also excluded regions that were resected, typically in the anterior medial temporal lobe (Suppl. Table S4). Here is show an incidence map identifying the regions most affected by signal drop out (blue and green colored regions), which includes areas where the fMRI signal is also affected by sinuses (orbitofrontal cortex). Orange to yellow color shows the percentage of human es-fMRI runs that were available for analysis (not masked by intracranial electrodes, weak signal or outside of the epileptic foci regions).

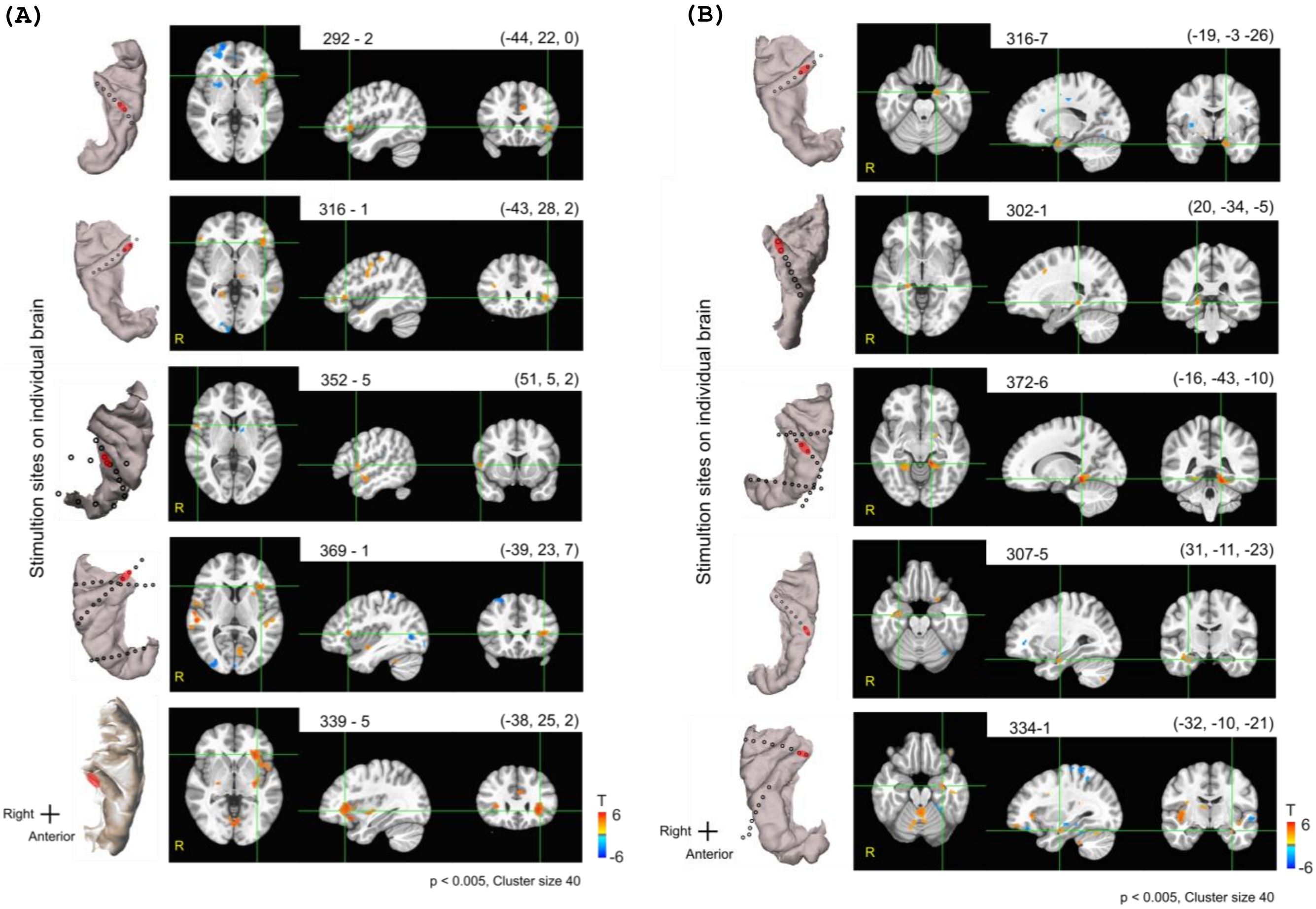

Supplementary Figure S3. Individual Human es-fMRI Activation Results Involving VLPFC and MTL, Related to Figure 3. (A) Shown are several individual human results showing substantial VLPFC (including inferior frontal gyrus) activation $p < 0.005$, cluster size = 40. Numbers above the panels indicate the subject number and testing run, followed by MNI x, y, z coordinates at the cross-hairs. The color bar shows the positive and/or negatively activated BOLD responses as T-values (range -6 to 6). (B) Individual human es-fMRI results involving MTL. Shown are several individual human results showing substantial MTL activation involving hippocampal or parahippocampal areas, $p < 0.005$, cluster size = 40. The color bar shows the positive and/or negatively activated BOLD responses as T-values (range -6 to 6).

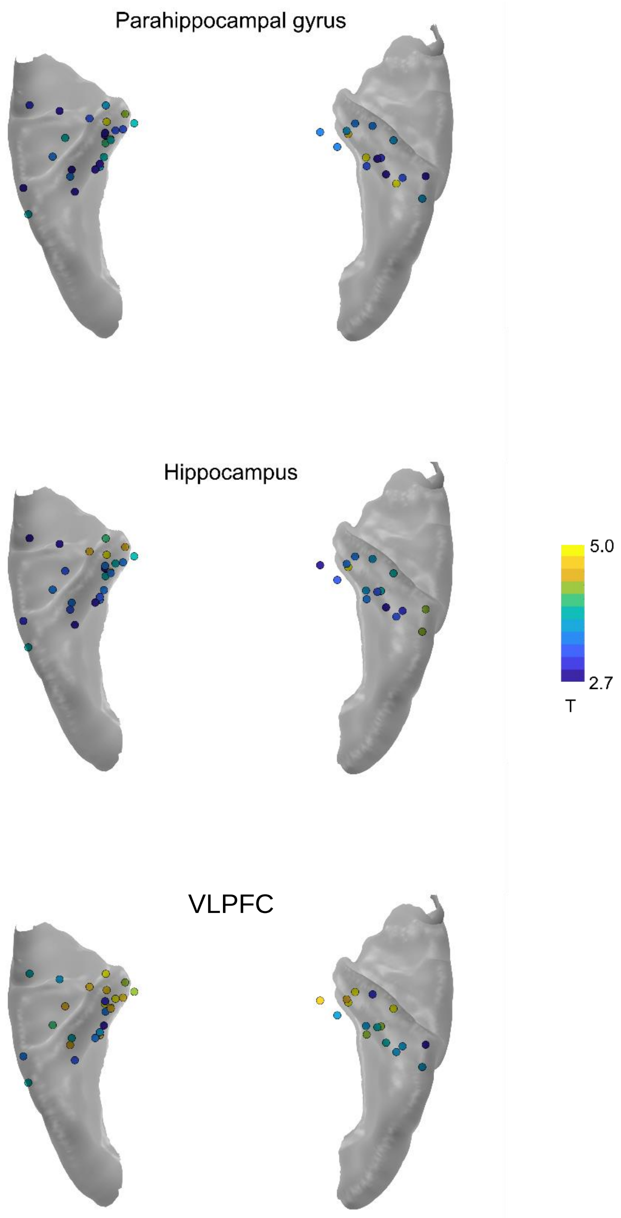

Supplementary Figure S4. Impact of Stimulation Sites on the MTL and VLPFC Contact Sites, Related to Figure 3. Shown are the auditory cortex regions that when stimulated resulted in the displayed strength of fMRI responses on VLPFC and MTL regions. Auditory cortex (Heschl's gyrus) stimulation sites are color coded from blue to yellow color map according to the strength of the fMRI BOLD response elicited in the parahippocampal, hippocampal, or VLPFC (IFG) regions. Although stronger responses tend to be seen from the more medial HG contacts, there are also contacts bordering other regions that also elicit strong responses and passive current spread is a factor, see manuscript text discussion. The color bar shows the positive and/or negatively activated BOLD responses as T-values (range -2.7 to 5).

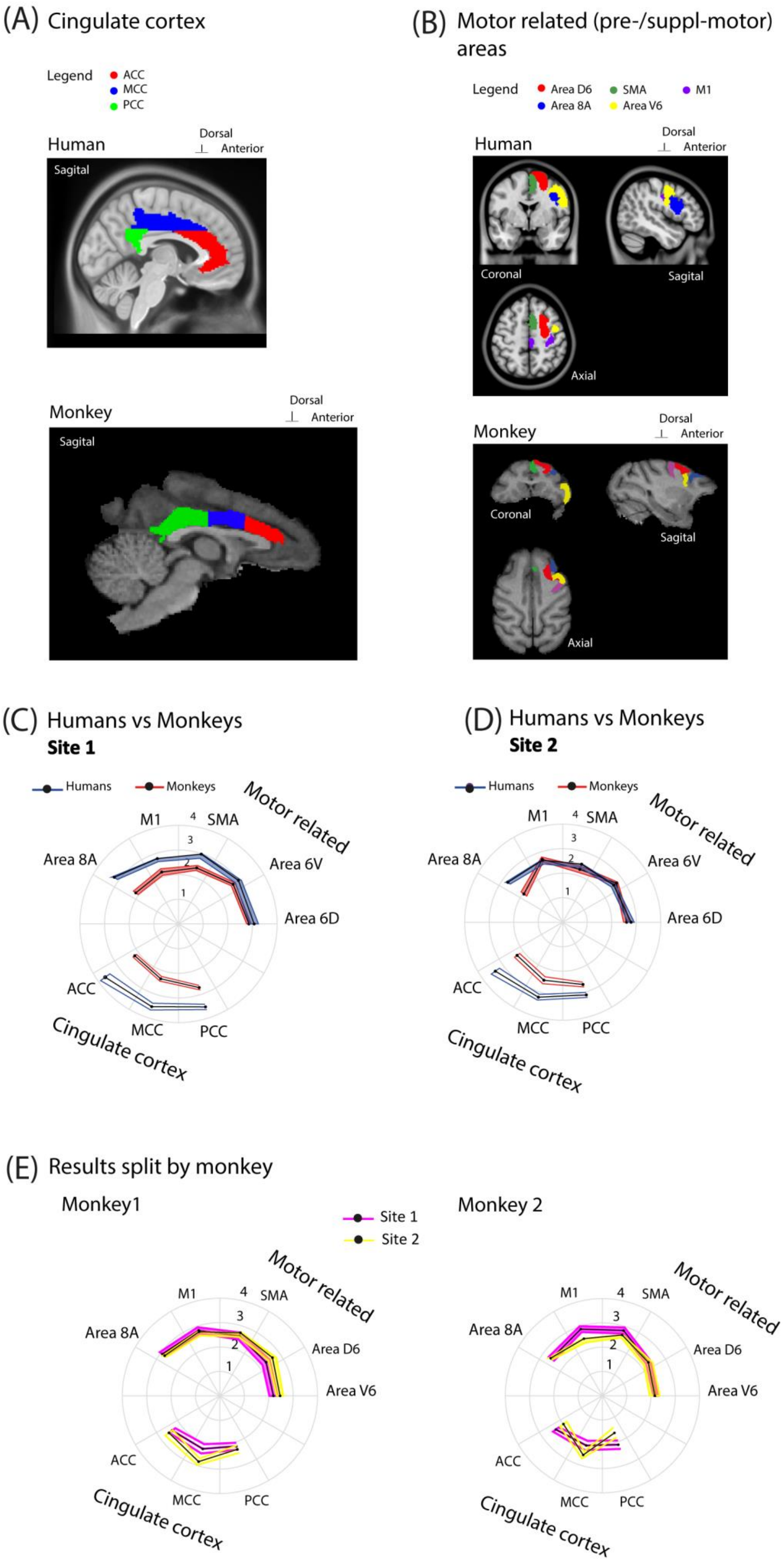

(A) Cingulate cortex
Legend
ACC
MCC
PCC
Human
Dorsal
Anterior
Sagital
Monkey
Dorsal
Anterior
Sagital
(B) Motor related (pre-/suppl-motor) areas
Legend
Area D6
Area 8A
SMA
Area V6
M1
Human
Dorsal
Anterior
Coronal
Sagital
Axial
Monkey
Dorsal
Anterior
Coronal
Sagital
Axial
(C) Humans vs Monkeys
Site 1
Humans
Monkeys
Motor related
M1
SMA
Area 8A
Area 6V
Area 6D
4
3
2
1
ACC
MCC
PCC
Cingulate cortex
(D) Humans vs Monkeys
Site 2
Humans
Monkeys
Motor related
M1
SMA
Area 8A
Area 6V
Area 6D
4
3
2
1
ACC
MCC
PCC
Cingulate cortex
(E) Results split by monkey
Monkey1
Site 1
Site 2
Monkey 2
Motor related
M1
SMA
Area 8A
Area D6
Area V6
4
3
2
1
ACC
MCC
PCC
Cingulate cortex
Motor related
M1
SMA
Area 8A
Area D6
Area V6
4
3
2
1
ACC
MCC
PCC
Cingulate cortex

Supplementary Figure S5. Vocal Production Associated (Motor Related) and Cingulate Cortex Areas: Human and Macaque ROI results, Related to Figure 5. (A) ROIs in cingulate cortex (ACC: anterior cingulate cortex; MCC: middle cingulate cortex; PCC: posterior cingulate cortex). (B) ROIs in motor and pre-/supplementary- motor areas, including vocal motor-production associated areas; Abbreviation: SMA, supplementary motor area. (C-D) human and monkey polar plots split by Site 1 and Site 2 stimulation showing a summary of the max *Z*-score (error boundaries: +/-SEM) across sessions/runs and participants. Format as in manuscript Figure 5. (E) Results split by monkey (M1 and M2).

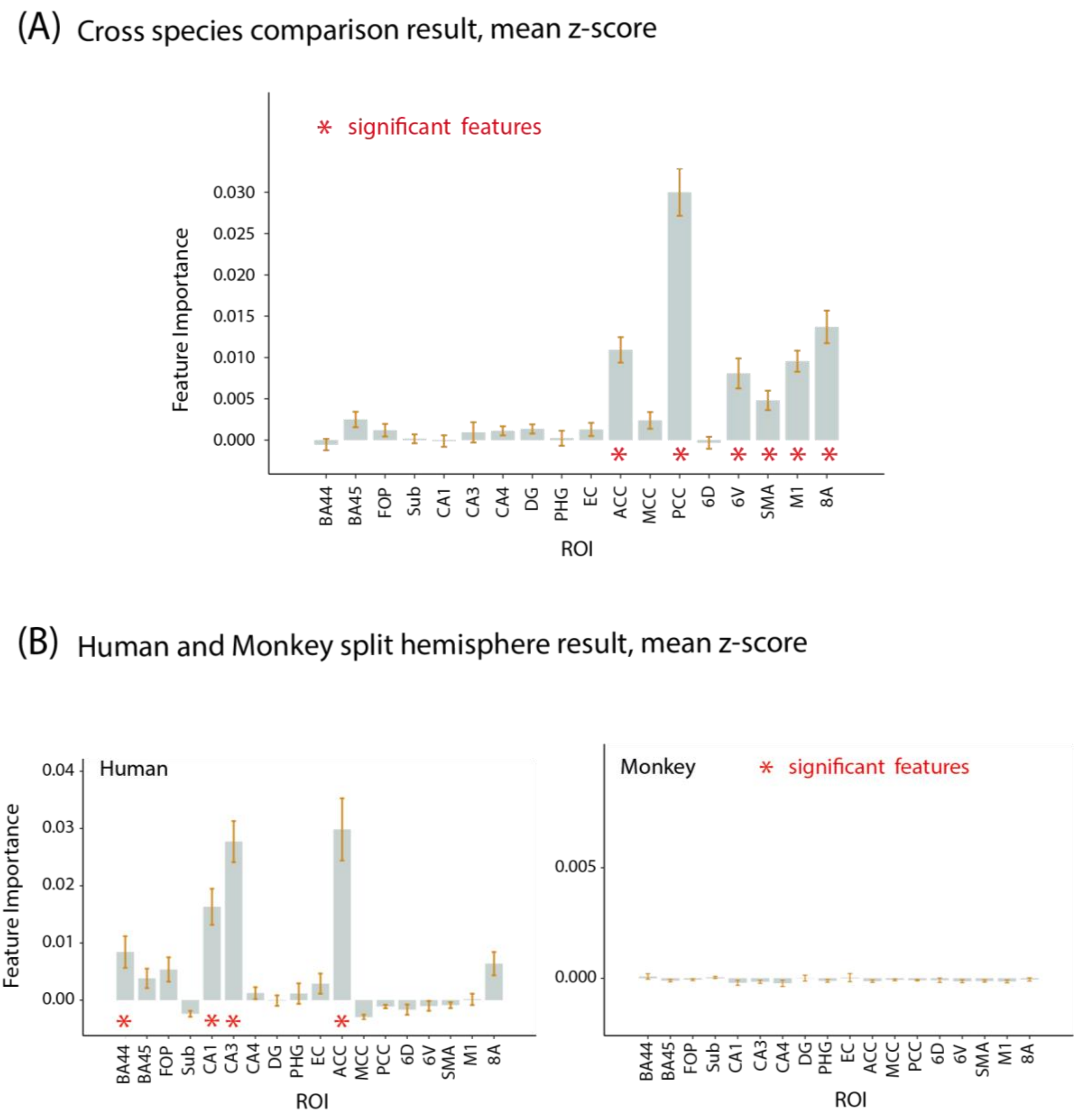

Supplementary Figure S6. Classifier Decoding Results: Species and Hemisphere, Related to Figure 5. (A) Species classification: Variable importance values from the Catboost classifier (see Methods). The larger fMRI response importance values in ACC, PCC, 6V, SMA, M1 and 8A are associated with the model predicting that the data points come from humans rather than monkeys. Red asterisks show significantly larger variable importance. Note how the classifier can only detect the differences in the data from the Cingulate and Vocal motor-related ROIs (right of and including the ACC) but is agnostic to the species difference in the VLPFC (areas 44, 45 and FOP) and MTL regions (Sub, CA1, CA3, CA4, DG, PHG and EC), see manuscript Discussion. (B) Response hemispheric laterality classification: Comparison of accuracy of hemispheric laterality classification, separately for the humans (left panel) and monkeys (right panel). This shows that the classifier can significantly attribute the hemisphere (positive feature importance is for the left hemisphere) in the es-fMRI response in human BA44, CA1, CA3 and ACC. The monkey data in the right panel show very small feature importance values, with the classifier not able to distinguish data from the two hemispheres in any of the regions. For both (A-B) 100 classification models were evaluated with different permutations. Accuracy is determined by 10-fold cross-validation (see Methods).
