# Supplementary material for: Common fronto-temporal effective connectivity in humans and monkeys
Source: Neuron. 2021 Mar 3;109(5):852–868.e8. doi: 10.1016/j.neuron.2020.12.026 (PMC7927917; doi:10.1016/j.neuron.2020.12.026)
Supplement: Document S2. Article plus supplemental information [file mmc6.pdf]

Neuron
Article

Common fronto-temporal effective connectivity in humans and monkeys

Graphical Abstract

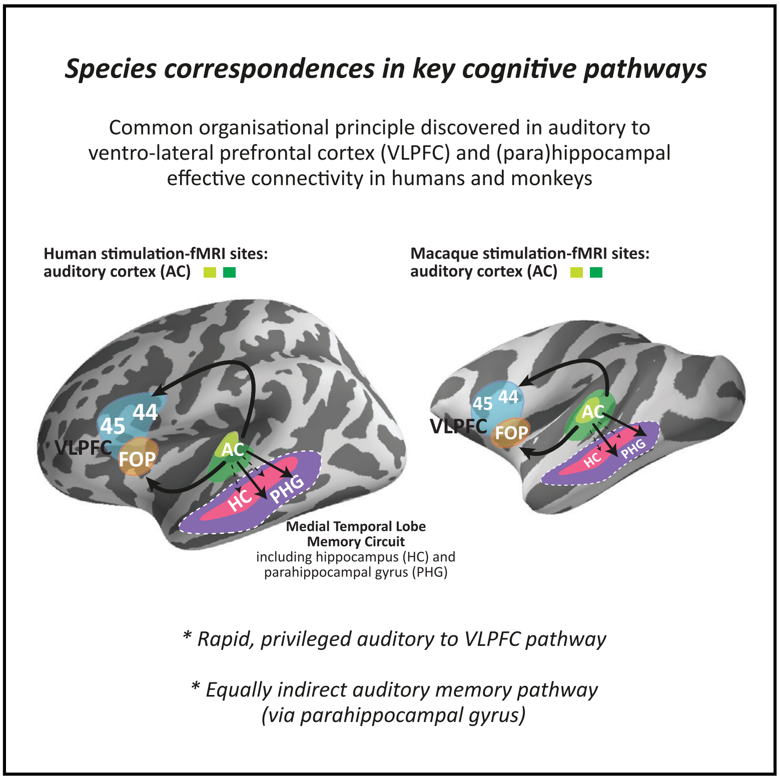

Highlights

- Privileged human auditory to ventral prefrontal connectivity, paralleled in monkeys
- Common auditory to parahippocampal effective connectivity in both species
- Stronger lateralization of connectivity effects in humans than in monkeys
- Human fronto-temporal network rooted in conserved organizational principle

Authors

Francesca Rocchi, Hiroyuki Oya, Fabien Balezeau, ..., Timothy D. Griffiths, Matthew A. Howard III, Christopher I. Petkov

Correspondence

fr93@leicester.ac.uk (F.R.),
hiroyuki-oya@uiowa.edu (H.O.),
chris.petkov@ncl.ac.uk (C.I.P.)

In Brief

Interconnectivity between brain regions crucial for language and declarative memory is thought to have substantially evolutionarily differentiated in humans. Rocchi, Oya and colleagues visualize site-specific brain connectivity in monkeys and human neurosurgery patients. They discover a common neural organizational principle that bridges gaps between primates as neurobiological models and human patients.

Rocchi et al., 2021, Neuron *109*, 852–868
March 3, 2021 © 2021 The Author(s). Published by Elsevier Inc.
https://doi.org/10.1016/j.neuron.2020.12.026

CellPress
OPEN ACCESS

Neuron

Article

Common fronto-temporal effective connectivity in humans and monkeys

Francesca Rocchi,[1,10,*] Hiroyuki Oya,[2,7,10,*] Fabien Balezeau,[1] Alexander J. Billig,[3] Zsuzsanna Kocsis,[1,2] Rick L. Jenison,[6] Kirill V. Nourski,[2,7] Christopher K. Kovach,[2] Mitchell Steinschneider,[5] Yukiko Kikuchi,[1] Ariane E. Rhone,[2] Brian J. Dlouhy,[2,7] Hiroto Kawasaki,[2] Ralph Adolphs,[4] Jeremy D.W. Greenlee,[2,7] Timothy D. Griffiths,[1,2,8] Matthew A. Howard III,[2,7,9] and Christopher I. Petkov[1,11,*]

[1]Biosciences Institute, Newcastle University Medical School, Newcastle upon Tyne, UK
[2]Department of Neurosurgery, The University of Iowa, Iowa City, IA, USA
[3]Ear Institute, University College London, London, UK
[4]Division of Biology and Biological Engineering, California Institute of Technology, Pasadena, CA, USA
[5]Departments of Neurology and Neuroscience, Albert Einstein College of Medicine, Bronx, NY, USA
[6]Department of Neuroscience, University of Wisconsin - Madison, Madison, WI, USA
[7]Iowa Neuroscience Institute, The University of Iowa, Iowa City, IA, USA
[8]Wellcome Centre for Human Neuroimaging, University College London, London, UK
[9]Pappajohn Biomedical Institute, The University of Iowa, Iowa City, IA, USA
[10]These authors contributed equally
[11]Lead contact
*Correspondence: fr93@leicester.ac.uk (F.R.), hiroyuki-oya@uiowa.edu (H.O.), chris.petkov@ncl.ac.uk (C.I.P.)
https://doi.org/10.1016/j.neuron.2020.12.026

SUMMARY

Human brain pathways supporting language and declarative memory are thought to have differentiated substantially during evolution. However, cross-species comparisons are missing on site-specific effective connectivity between regions important for cognition. We harnessed functional imaging to visualize the effects of direct electrical brain stimulation in macaque monkeys and human neurosurgery patients. We discovered comparable effective connectivity between caudal auditory cortex and both ventro-lateral prefrontal cortex (VLPFC, including area 44) and parahippocampal cortex in both species. Human-specific differences were clearest in the form of stronger hemispheric lateralization effects. In humans, electrical tractography revealed remarkably rapid evoked potentials in VLPFC following auditory cortex stimulation and speech sounds drove VLPFC, consistent with prior evidence in monkeys of direct auditory cortex projections to homologous vocalization-responsive regions. The results identify a common effective connectivity signature in human and nonhuman primates, which from auditory cortex appears equally direct to VLPFC and indirect to the hippocampus.

INTRODUCTION

Brain networks adapted for specialized functions typically show direct, rapid, or effective connectivity between regions crucial for behavior. Finding such connectivity, alongside evidence for evolutionary homology, convergence, or divergence, can be of substantial theoretical significance. Within the motor domain, human and nonhuman primates have direct cortico-spinal projections subserving fine movement control that are indirect in rodents (Lemon, 2008). Also, human laryngeal motor cortex projects directly to a brain stem nucleus (ambiguus) controlling laryngeal muscles (Simonyan and Horwitz, 2011). Such projections for vocal production are potentially more indirect in nonhuman primates (Archakov et al., 2020; Simonyan and Horwitz, 2011) and rodents (Arriaga et al., 2012), which sheds light on human speech evolution (Belyk and Brown, 2017; Boë et al., 2019; Jarvis, 2019; Jürgens, 2002) and convergent evolution in songbirds (Petkov and Jarvis, 2012; Roberts et al., 2017).

Language defines us as a species, and because of its prominent role in declarative memory, evolutionary differentiation of human cognitive pathways is expected. Comparative studies often see considerable levels of conservation alongside insights on species-specific differences (Balezeau et al., 2020; Donahue et al., 2018; Eichert et al., 2019; Flinker and Knight, 2018; Friederici, 2017; Neubert et al., 2014; Pryluk et al., 2019). Yet certain cross-species comparisons are missing, such as on the impact of directed effective connectivity with the required precision of site-specific perturbation that can be applied to both human and nonhuman primates. Thereby, the question of the extent of differentiation versus conservation in primate fronto-temporal systems—although crucial for understanding

 © 2021 The Author(s). Published by Elsevier Inc.
This is an open access article under the CC BY license (http://creativecommons.org/licenses/by/4.0/).

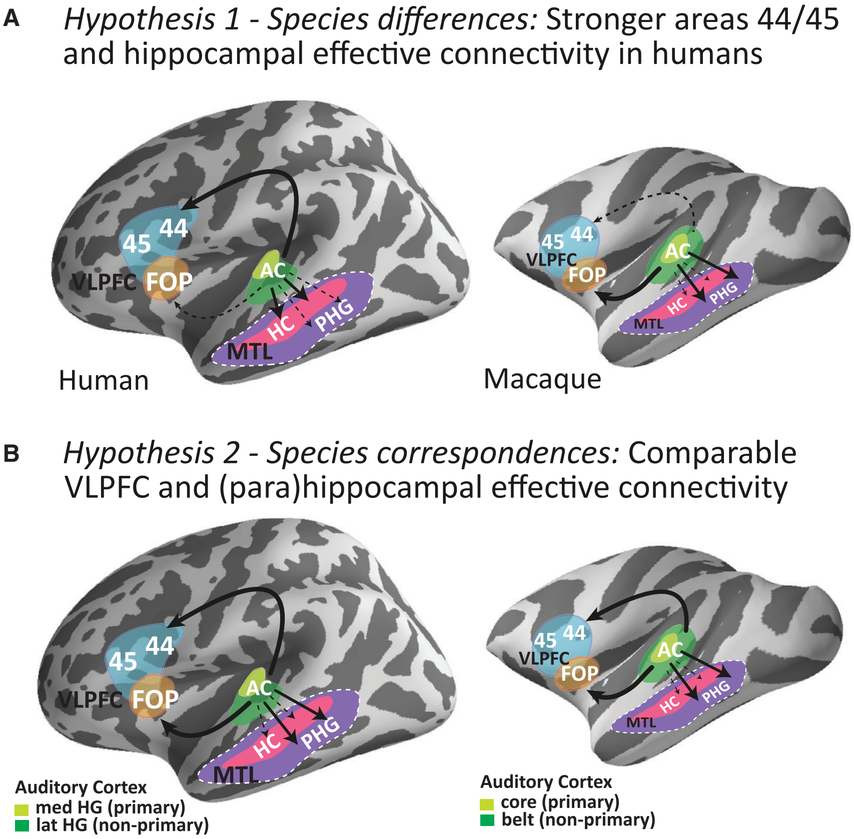

Figure 1. Illustrated hypotheses involving effective connectivity from auditory cortex to VLPFC and MTL areas in humans and monkeys

(A) Hypothesis 1 predicts stronger effective connectivity from stimulating auditory cortex on human VLPFC areas 44/45 than in monkeys, because of the prominence of the dorsal pathway in humans interconnecting these areas with auditory cortex. By comparison, the ventral pathway interconnecting auditory cortex with areas such as the frontal operculum (FOP) is structurally dominant in nonhuman primates. Thus, by this hypothesis, effective connectivity in the monkeys could be stronger in FOP than areas 44/45. MTL effects by some accounts are expected to be stronger in human hippocampal subregions than adjacent areas, such as the parahippocampal gyrus (PHG). In monkeys, the reverse pattern is expected; see text.

(B) Hypothesis 2 predicts cross-species correspondences in auditory effective connectivity with these VLPFC and MTL regions.

AC, auditory cortex; Brodmann areas 44 and 45; FOP, frontal operculum; HC, hippocampus; latHG, lateral Heschl's gyrus; medHG, medial Heschl's gyrus; MTL, medial temporal lobe; PHG, parahippocampal gyrus; VLPFC, ventro-lateral prefrontal cortex.

which aspects of human language and cognition can find realistic nonhuman animal models—remains open.

Speech and language are supported by a fronto-temporal network, including auditory and ventro-lateral prefrontal cortex (VLPFC) areas, interconnected via dorsal and ventral white matter pathways and processing streams (Rauschecker and Scott, 2009). Left hemisphere areas posterior to Heschl's gyrus (HG, an anatomical landmark associated with auditory cortex) are interconnected with Brodmann area 44 and parts of area 45 in the VLPFC by way of the dorsal arcuate fasciculus pathway (Catani et al., 2005; Friederici, 2017). There is now evidence for an auditory homolog of this dorsal pathway in chimpanzees and macaques, with its hemispheric lateralization seen as a prominent human-specific distinction (Balezeau et al., 2020). In monkeys, VLPFC neurons respond to vocalization sounds (Romanski and Goldman-Rakic, 2002), and neuronal tracer studies have shown evidence for directional connectivity between non-primary auditory (lateral belt) areas and VLPFC (Romanski et al., 1999b). Whether similar auditory to inferior frontal interconnectivity exists in humans and nonhuman primates is unclear (Garell et al., 2013; Nakae et al., 2020; Neubert et al., 2014). One hypothesis, based on the idea that the human dorsal pathway and VLPFC areas 44/45 differentiated for language (Friederici, 2017)—supported by structural evidence that the dorsal arcuate fasciculus pathway is predominant in humans whereas the ventral pathways predominate in nonhuman primates (Balezeau et al., 2020; Friederici, 2017; Rilling et al., 2012)—is that human auditory cortex might have greater effective connectivity impact on area 44. By contrast, effects on the adjacent frontal operculum (FOP) may be stronger in monkeys (Wilson et al., 2017; Figure 1A). An alternative hypothesis is that, structural connectivity differences notwithstanding, there may be substantial cross-species correspondence in effective connectivity from auditory cortex to these areas (Figure 1B).

Sensory input to the medial temporal lobe (MTL) in humans is important for declarative memory and may also have evolutionarily differentiated in humans. Monkey recognition memory for sounds is surprisingly more fleeting than for visual items (Fritz et al., 2005), yet so is human memory, particularly when sounds are difficult to verbally label (Schulze et al., 2012). Also, studies in nonhuman animals (rodents, cats, and monkeys) show primarily indirect projections from auditory cortex to the hippocampus (Munoz-Lopez et al., 2010) via parahippocampal/perirhinal cortex (Cenquizca and Swanson, 2007; Insausti and Amaral, 2008). Thus, one prediction is that auditory effective connectivity to the MTL in humans may be stronger in the hippocampus than parahippocampal cortex (Figure 1A). However, human neuroimaging studies have not demonstrated direct auditory to hippocampal connectivity, rather recent studies suggest indirect interconnectivity with auditory cortex (via parahippocampal cortex; Maller et al., 2019), as seen in other species. Another hypothesis, given that language assists humans in naming and conceptualizing sounds and thus better remembering them (Schulze et al., 2012), is that the auditory-to-MTL network is based on a largely evolutionarily conserved organizational principle (Figure 1B).

New approaches for assessing effective connectivity similarly across the species could shed light on human cognitive

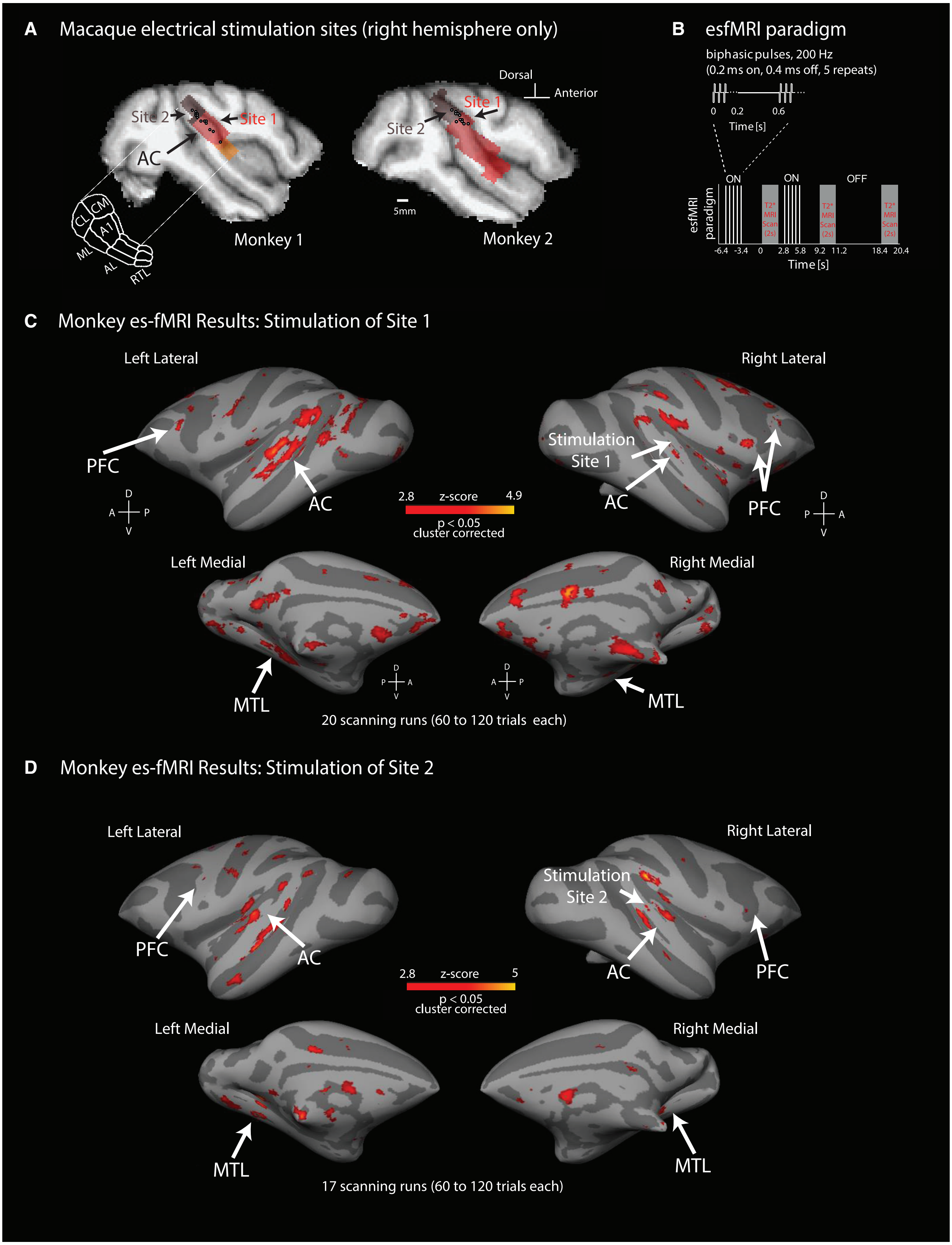

(legend on next page)

CellPress
OPEN ACCESS

pathways and their primate origins or specialization. In monkeys, electrical stimulation combined with functional magnetic resonance imaging (es-fMRI) was developed to visualize the impact of site-specific stimulation on neural network effective connectivity (Klink et al., 2020; Tolias et al., 2005). Direct electrical brain stimulation is a common treatment for debilitating brain disorders and has a history of being combined with functional imaging in humans (Klink et al., 2020; Phillips et al., 2006; Rezai et al., 1999). Recently, following substantial safety testing, the es-fMRI method has resurged as an approach in human patients being monitored for neurosurgery (Oya et al., 2017) and a human es-fMRI resource was established (Thompson et al., 2020). The human es-fMRI approach now offers the possibility for direct cross-species comparison. We conducted a first comparative study of es-fMRI effects obtained by stimulating auditory cortex in human neurosurgery patients and macaques. Electrical stimulation of auditory cortex induced differential es-fMRI activity in several VLPFC (areas 44, 45, and FOP) and MTL subregions (including parahippocampal and hippocampal areas). The effective connectivity patterns were remarkably similar across the species. In humans, we also studied electrical stimulation tractography to assess the latency of interconnectivity between auditory cortex, VLPFC, and MTL. Finally, we observed strong neurophysiological responses to speech sounds in human VLPFC and establish directional effective interconnectivity with auditory cortex.

RESULTS

The es-fMRI approach has been used in several disorders to assess the pattern of activated brain regions and the treatment response from stimulating specific brain sites (Phillips et al., 2006; Rezai et al., 1999). Safety issues are a challenge (Boutet et al., 2019), but breakthroughs have been made (Oya et al., 2017). The es-fMRI approach in humans has benefitted substantially from information obtained in nonhuman primates using the same technique (Klink et al., 2020). es-fMRI electrically stimulates neurons and axons in specific sites, and the resulting fMRI activity observed during site-specific electrical stimulation can identify effective connectivity in target regions, including the synaptic efficacy of post-synaptic neurons. Although the mode of action is not fully understood, such information is accumulating in nonhuman primates (Klink et al., 2020). The results of this paper shed further light on es-fMRI effects and whether effects are largely antidromic or orthodromic.

Auditory cortex es-fMRI in macaques

We first studied the es-fMRI response from stimulation of caudal auditory cortical sites in the right hemisphere of two macaques. Auditory cortex es-fMRI induced significantly stronger activity relative to non-stimulation trials (Figure 2; $p < 0.05$; cluster corrected; $Z > 2.8$) in areas including auditory cortex, motor-related, prefrontal, and MTL regions (Table S1). MTL activation included parahippocampal gyrus (Munoz-Lopez et al., 2010).

Grouping of stimulation sites was conducted across two tonotopically organized fields (“core” site 1 and “belt” site 2; Figure 2). In monkeys, the auditory core is associated with primary auditory cortex (Figure 1, yellow area in right panels) and the auditory belt with non-primary auditory cortex (Figure 1, green area in right panels); note that the monkey homolog of human HG (also known as the transverse temporal gyrus) may not exist, but there is general cross-species correspondence in the presence of core (primary-like) and belt (non-primary) areas (Rauschecker, 1997). However, es-fMRI results from stimulating these two sites in the monkeys were statistically indistinguishable (no significant voxels surviving the $p < 0.05$ cluster-corrected threshold for the site 1 versus site 2 contrast or vice versa). We suspected that this might arise because of partially overlapping stimulation, which was confirmed by estimating the passive current spread using the formula $r = \sqrt{I/K}$, where I is current and K a constant of pyramidal cell excitability (for a 200-μs pulse, we used a K value of 1.3 mA/mm^2; Tehovnik et al., 2006; Tolias et al., 2005). The resulting radius (r) of passive current spread is 0.9 mm, suggesting that stimulating core auditory cortex sites also passively electrically stimulates portions of the adjoining auditory belt and vice versa, a consideration for interpreting the es-fMRI results. For additional macaque es-fMRI results, split by monkey or combining the two sites of stimulation, see Figure S1.

Auditory cortex es-fMRI in humans

We assessed the es-fMRI activity in response to human auditory cortex electrical stimulation of depth electrodes at medial or lateral sites in either left or right HG (Figure 3; Figure S2A shows individual subject contact locations). Human primary auditory cortex tends to include parts of HG (Da Costa et al., 2011), particularly the medial segment (Figure 1, yellow area in left panels). The more lateral segments of HG that extend on the superior temporal gyrus (STG) are considered non-primary auditory cortex (Figure 1, green area in left panels). Stimulation sites were subdivided into site 1 (medHG), grouping contacts in medial HG areas where significant phase-locking to high-repetition click sound rates occur (STAR methods), and site 2, grouping lateral HG and planum temporale contacts (latHG+PT) lacking high click rate neural phase locking (Figures 3A and S2B). Figure S2C shows the amount of data retained following removal of neurosurgically resected sites and lost data around electrode contacts.

Figure 2. Macaque monkey auditory cortex electrical stimulation sites and es-fMRI results

(A) Stimulation sites 1 and 2 in the right hemisphere auditory cortex in the two macaques (M1 and M2), overlaid on fMRI tonotopic parcellation of auditory cortex (see STAR methods).

(B) Illustrated es-fMRI paradigm timing, not to scale.

(C and D) Macaque es-fMRI group results showing significantly activated voxels during auditory cortex stimulation relative to no-stimulation trials: site 1 (C) and site 2 (D); cluster-corrected $p < 0.05$; $Z > 2.8$ (see Table S1 for list of activated anatomical regions). Results projected to the surface-rendered macaque template brain are shown.

PFC, prefrontal cortex.

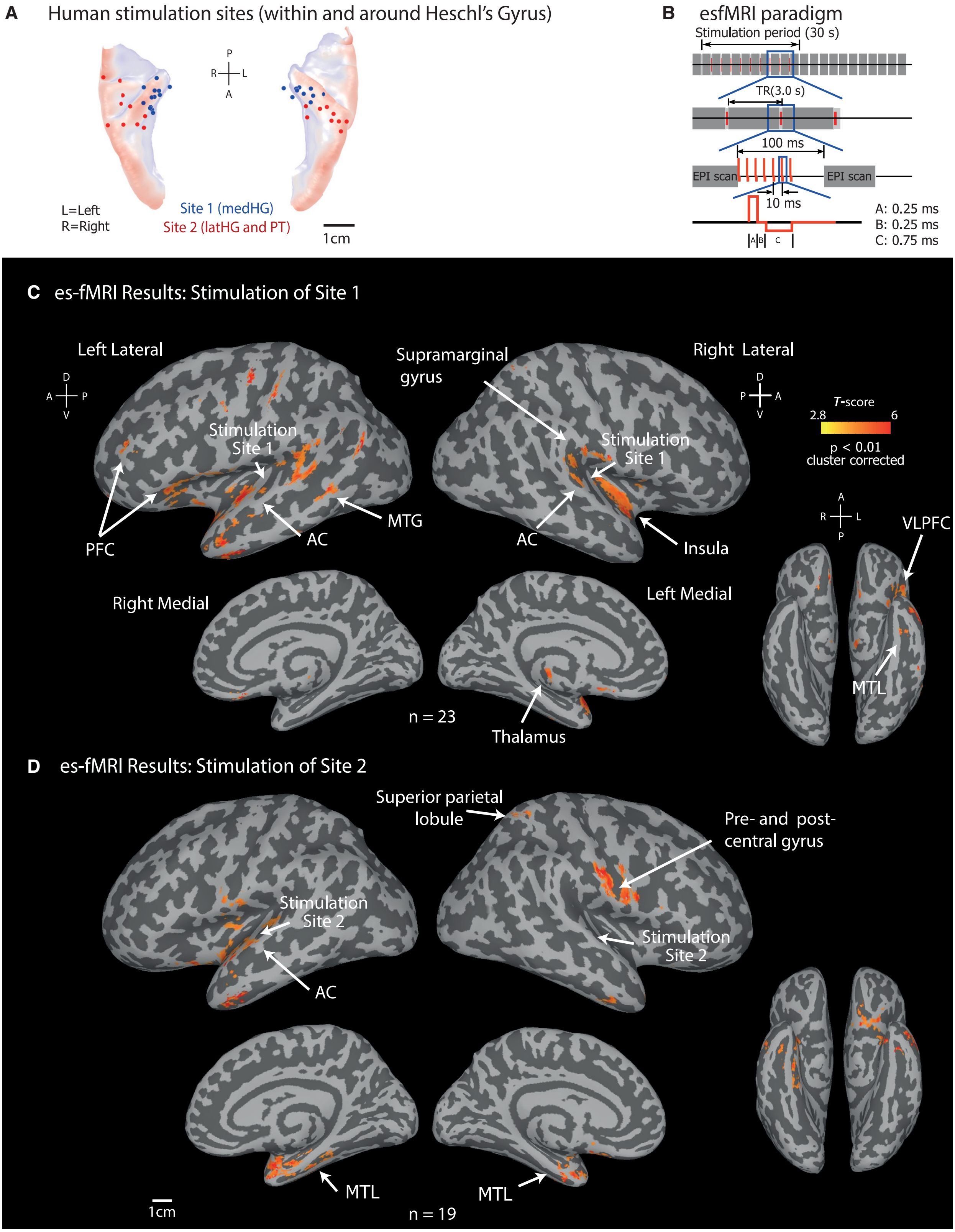

(legend on next page)

CellPress
OPEN ACCESS

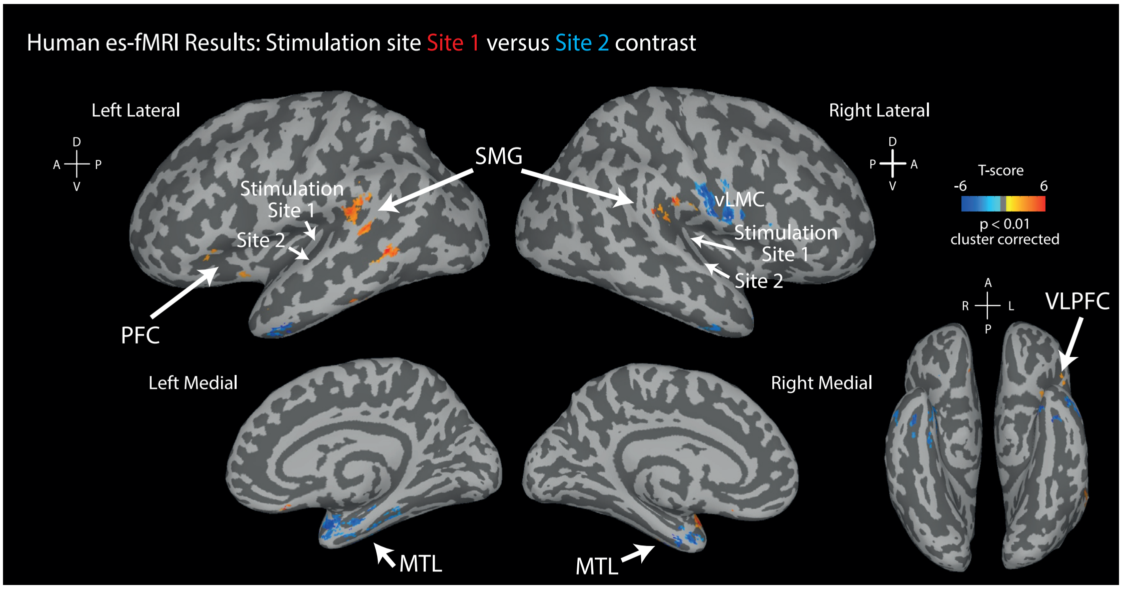

Figure 4. Human results contrasting site 1 versus site 2 es-fMRI effects

Same format as in Figures 3C and 3D, showing statistically significant ($p < 0.01$ cluster-corrected) effects where either site 1 (red color map) or site 2 (blue color map) was stronger. The corresponding contrast in monkeys yielded no cluster-corrected differences. SMG, supramarginal gyrus; vLMC, approximate location of ventral laryngeal motor cortex within M1.

Significant es-fMRI effects (Figure 3C; cluster corrected $p < 0.01$) from stimulating site 1 (medHG) included auditory cortex (STG) and VLPFC (including inferior frontal gyrus; Table S2). Site 2 (latHG+PT) stimulation activated areas including STG and MTL (Figure 2D). In some individual subjects, the activity response was significant in VLPFC and MTL (Figure S3). The strength of the es-fMRI response in VLPFC and MTL overlaid on the stimulated HG contacts that produced it is shown in Figure S4.

Unlike the monkey es-fMRI results, which did not significantly differ between the two auditory cortex stimulation sites, effects from stimulating the two sites in humans differed. We calculated the passive current spread as above: resulting in a 3-mm radius around the stimulation contact pairs, which are separated by 5–10 mm. The analytical group contrast of site 1 (medHG) versus site 2 (latHG+PT) showed stronger VLPFC activity from stimulating site 1 (Figure 4; Table S2). Site 1 stimulation also resulted in stronger activity in more posterior temporal and parietal areas (e.g., supramarginal gyrus). Site 2 stimulation resulted in stronger activity in anterior temporal areas (Table S2).

Macaque es-fMRI effects in VLPFC and MTL subregions

We assessed whether macaque auditory cortex stimulation differentially activates anatomically defined areas 44, 45, and the FOP in the VLPFC (Figures 5A and 5B). Region of interest (ROI) effects were tested with a mixed-design analysis of variance (ANOVA): between-subjects factor of monkeys; within-subjects factors of activated hemisphere (left or right); ROI (area 44, 45, or FOP); and stimulation site as covariate (site 1 or 2). Model assumptions for this and all subsequent reported analyses were met or corrected as indicated. Planned post hoc tests were used to identify differential effects across the ROIs.

The overall magnitude of the fMRI signal in the VLPFC ROIs was stronger in macaque 1 (M1) (significant monkey factor: $F_{1,34} = 7.34$; $p = 0.010$), but there were no interactions with other factors, suggesting a similar pattern of es-fMRI effects across the monkeys. The two sites of stimulation did not differentially affect the VLPFC fMRI response, consistent with the whole-brain results. The macaque VLPFC ROI effects were also statistically indistinguishable across the two hemispheres (no significant hemisphere effect). Although the ANOVA revealed only a trend toward a differential fMRI response across the ROIs ($F_{2,33} = 3.04$; $p = 0.054$), the planned post hoc comparisons identified stronger es-fMRI activity in the FOP over areas 45 and 44 in that order (all $p < 0.002$; Bonferroni corrected; Figures 5A and 5B).

Monkey MTL subregions showed differential es-fMRI activity during auditory cortex stimulation (Figure 5C). A mixed-design

Figure 3. Human auditory cortex electrical stimulation sites and es-fMRI results

(A) Stimulation of depth electrodes in the transverse temporal gyrus (Heschl's gyrus [HG]). Human auditory cortex stimulation sites 1 and 2 are shown, looking down on the superior temporal plane. Stimulation sites are identified at the center of the adjacent contacts used for stimulation (Figure S2A shows actual contact locations in each subject).

(B) es-fMRI paradigm timing.

(C and D) Human es-fMRI group results shown as significantly activated voxels during stimulation relative to no-stimulation trials (cluster corrected; $T = 2.8$; $p < 0.01$) for site 1 (C) and site 2 (D), shown on the surface-rendered Montreal Neurological Institute human standard brain template.

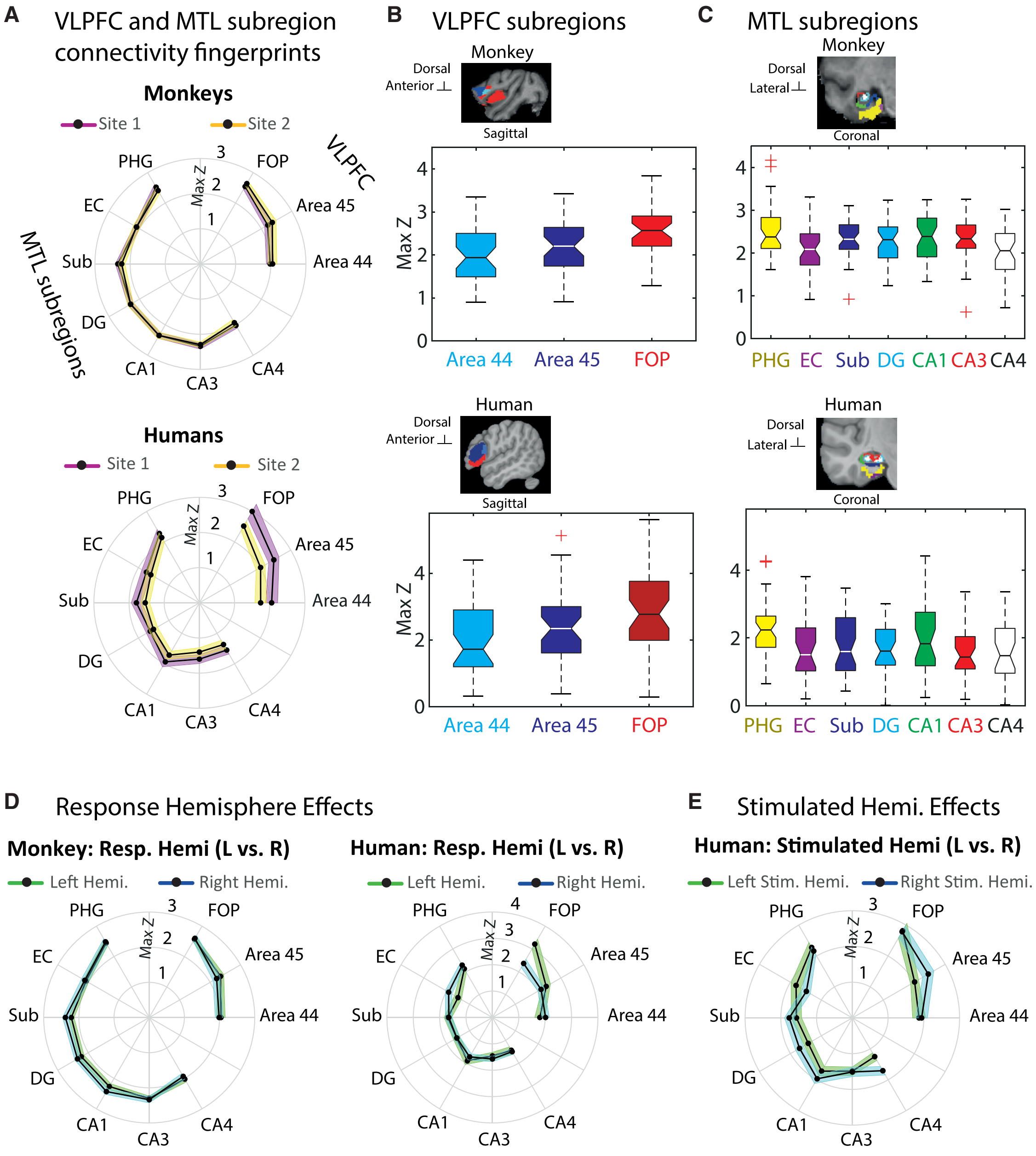

Figure 5. Human and macaque VLPFC and MTL connectivity profiles

(A) VLPFC and MTL subregion es-fMRI effects displayed as polar plots. Shown are across scanning run peak Z values and variability (±SEM [standard error of the mean]). Top plot in (A) shows monkey results; bottom plot shows human results.

(B and C) Whisker plots of VLPFC (B) and MTL (C) es-fMRI activity responses (across scanning runs, peak Z value; central mark identifies the median; edges of box are 25th and 75th percentiles; whiskers extend to extreme ends of data, not including outliers in red crosses; non-overlapping notches are significantly different at $p < 0.05$). Also shown are sagittal and coronal slices in each species with the anatomically localized ROIs used for the analysis.

(D) Effects by response hemisphere (monkeys left, humans right).

(E) Human effects by stimulated hemisphere; only right hemisphere was stimulated in the monkeys.

Note that the joined lines in the polar plots are not intended to suggest a continuing pattern across ROIs, only to assist in comparison of the patterns across species (A) and hemispheres (D and E); also see the whisker plots in (B) and (C).

ANOVA including the seven anatomically delineated MTL subregions showed that es-fMRI activity in these ROIs did not differ between monkeys, suggesting similar effects in both monkeys. Effects also did not differ across the two stimulation sites or across the two hemispheres, as seen for the VLPFC results. The MTL ROIs differed nonlinearly in their fMRI activity response, showing a more complex pattern than just a linear change in the activity response across the ROIs (cubic effect: $F_{1,34} = 4.27$; p = 0.028;

CellPress
OPEN ACCESS

Figures 5A and 5C). The planned post hoc comparisons showed stronger es-fMRI activity in the parahippocampal gyrus (PHG) than both entorhinal cortex (EC) and Cornu Ammonis (CA) field CA4 ($p < 0.003$; Bonferroni corrected). Subiculum (Sub), dentate gyrus (DG), CA1, and CA3 also had stronger activity than CA4 (all $p < 0.015$; Bonferroni corrected).

Human auditory cortex es-fMRI effects in VLPFC and MTL

We studied the human VLPFC and MTL effects in anatomically defined ROIs. Effects were tested with a mixed-design ANOVA (between-subjects factor: human subject; within-subject factors: ROI subregion, activated hemisphere, left or right; site of stimulation and stimulated hemisphere, left or right, as covariates). The vast majority of subjects were left hemisphere language dominant (Table S3).

For VLPFC, we observed differential activity of areas 44, 45, and FOP ($F_{1,21} = 5.26$; $p = 0.032$; Figure 5B) in a similar pattern as in the monkeys. The post hoc tests showed a stronger FOP response than in areas 45 and 44 ($p < 0.003$; Bonferroni corrected). Site of stimulation (site 1 versus site 2) effects on VLPFC only trended ($F_{1,21} = 3.14$; $p = 0.090$), seen as a weaker albeit not significant site 2 (latHG+PT) es-fMRI effect on the VLPFC subregions (Figure 5A). Hemispheric differences for certain ROIs were evident: visualized in Figure 5D as a stronger FOP response on the left, with the right hemisphere showing similar activity across the three VLPFC ROIs. Activated hemisphere effects did not interact with VLPFC ROI but did with site of stimulation (site 1 versus site 2 by activated hemisphere interaction: $F_{1,21} = 7.23$; $p = 0.014$) and stimulated hemisphere (stimulated hemisphere by activated hemisphere interaction: $F_{1,21} = 4.68$; $p = 0.042$; Figure 5E).

For the human MTL ROI effects, the ANOVA did not show significant differential activation between the ROIs, although the weaker pattern was similar to that seen in monkeys with the post hoc comparisons: The PHG had a stronger es-fMRI response than CA4 ($p = 0.016$; Bonferroni corrected) and DG ($p = 0.026$; corrected), and the subiculum response was stronger than CA4 ($p = 0.034$; corrected). MTL ROI effects only trended for activated hemisphere ($F_{1,10} = 3.35$; $p = 0.097$), seen in Figure 5D as a weaker, albeit not significant, right hemisphere bias, particularly for entorhinal cortex (Figures 5D and 5E). No other significant effects or interactions were observed.

Cross-species auditory cortex es-fMRI comparisons in VLPFC and MTL

We conducted cross-species es-fMRI comparisons of the VLPFC and MTL ROI responses (Figure 5 shows the anatomically delineated ROIs in both species). Statistical testing included species as a between-subjects factor in the mixed-design ANOVA.

For the VLPFC cross-species comparison, both monkey and human es-fMRI results showed stronger FOP responses than area 45 (ROI effect: $F_{1,57} = 9.68$; $p = 0.003$). There was no significant species difference in the VLPFC ROI effects ($p = 0.139$), showing statistically indistinguishable VLPFC ROI response patterns across the species (Figures 5A and 5B). An effect was found for activated hemisphere (left stronger than right; $F_{1,57} = 4.21$; $p = 0.045$) but did not significantly interact with species. There were higher order interactions with species, including hemispheric differences (VLPFC ROIs by species: $F_{1,57} = 4.97$, $p = 0.010$; ROIs by activated hemisphere by species: $F_{2,57} = 7.11$, $p = 0.002$), showing that a key difference across the species for the VLPFC areas is the hemispheric lateralization pattern being stronger in humans.

For the MTL, the overall es-fMRI activity level differed across the species (Figure 5D; $F_{2,46} = 10.55$; $p < 0.001$). The ROIs did not differ in their fMRI response pattern but interacted with species (Greenhouse-Geisser corrected: $F_{7.63,175.52} = 3.05$; $p = 0.004$; $\varepsilon = 0.636$), and the species factor further interacted with activated hemisphere (Greenhouse-Geisser corrected: $F_{8.70,200.16} = 2.175$; $p = 0.027$; $\varepsilon = 0.725$), suggesting that hemispheric lateralization in humans was a prominent species difference, as seen for the VLPFC results. For instance, human entorhinal cortex shows greater right hemisphere activation (Figure 5D), and this region was relatively more activated when the left human auditory cortex was stimulated (Figure 5E). By contrast, the monkey effects were statistically indistinguishable across the hemispheres. No other effects or higher order interactions were significant.

Classifier decoding results for the VLPFC and MTL subregions

The ANOVA analyses relied on peak/max ROI Z score values, because these data were well distributed and had very few outliers (Figure 5). Mean Z score values across voxels can provide important information but did not fit the analysis assumptions and could not be analyzed in the same way. We thus applied a machine learning classifier approach in the analysis of these data using a gradient boosting algorithm, Catboost (Prokhorenkova et al., 2017), after demeaning and normalizing the variability of these data (see STAR methods). The results with the classifier were highly complementary to the ANOVA results, in that the classifier could not differentiate the two species from the es-fMRI responses in the VLPFC and MTL ROIs (Figure S6). It is not the case that the classifier was not working or sensitive enough, because results with it recapitulated some of the hemispheric lateralization effects seen in humans and the observation that the macaque es-fMRI results are highly symmetrical across the two hemispheres. Namely, the classifier could significantly distinguish the hemisphere for several ROIs in the human results, but not for the macaque ROIs (Figure S6B).

Insights on macaque and human es-fMRI effects involving vocal motor-associated areas

Although the focus of the study was on auditory effective connectivity with the defined VLPFC and MTL areas, auditory cortex also projects via dorsal pathways to areas associated with vocal motor production, including those that support auditory-motor mapping (Archakov et al., 2020; Dichter et al., 2018; Jürgens, 2002; Petkov and Jarvis, 2012; Rauschecker and Scott, 2009; Simonyan and Horwitz, 2011). To gain insights on auditory cortex es-fMRI effects in vocal motor-related frontal areas, we conducted similar ANOVA and classifier analyses of the macaque and human es-fMRI results in cingulate cortex (anterior, middle, and posterior segments) and motor or pre-/supplementary-motor areas (including areas 6d, 6v, supplementary motor area, M1, and area 8). The ROIs used for both species and the polar plot results are shown in Figure S5.

Although, the Catboost classifier results showed interesting cross-species differences in a number of these ROIs (ACC, Anterior Cingulate Cortex; PCC, Posterior Cingulate Cortex; SMA, Supplementary Motor Area; M1, area 8, and 6v; Figure S6), the ANOVA results, however, did not statistically support a cross-species difference, unlike the consistency in results seen with both the classifier and ANOVA results for VLPFC areas 44, 45, and FOP and the MTL subregions. The ANOVA results for the motor-related areas did not show a significant species factor ($p = 0.143$) and did not significantly interact with ROI ($p = 0.115$). This may have resulted because of a potential difference in effects between sites of stimulation where interestingly the site 2 stimulation effects were at least qualitatively more similar across the species (Figure S5). However, even these site of stimulation effects were not significant in the ANOVA results and did not significantly interact with species and/or ROI (all $p > 0.143$).

The cingulate cortex results with the ANOVA were more consistent with the classifier results (Figure S6), as follows. The species factor was significant ($F_{2,75} = 21.315$; $p < 0.001$), and there was a significant ROI-by-species interaction (Greenhouse-Geisser corrected; $F_{3.192,119.712} = 4.125$; $p = 0.007$; $\varepsilon = 0.845$). For the cingulate cortex ROIs, there was no significant site of stimulation effect or higher order interactions (all $p > 0.376$; see Discussion).

Human electrical stimulation tractography

In the human patients, clinical electrode coverage could include frontal, auditory, and hippocampal sites. We studied neurophysiological connectivity with electrical tractography (esT) between auditory cortex, VLPFC, and hippocampus (Figures 6A and 6F–6H show the stimulation sites and recording contacts).

Auditory cortex site 1 (medHG) and site 2 (latHG+PT) stimulation induced neurophysiological potentials in VLPFC (Figures 5B and 5C) as soon as they could be measured after the 0–3 ms electrical stimulation artifact (Figure 6D; Videos S1 and S2; additional exemplary movies: https://osf.io/4axx2/). We assessed the polarity and latency of stimulation-induced responses using spatial Laplacian filtering to reduce volume conduction effects and spurious cross-channel correlated responses. Response waveforms in VLPFC showed positive and negative components at different latencies (Figures 6E–6H). Our labeling convention follows prior reports (Brugge et al., 2003; Garell et al., 2013), identifying positivities (*P*) and negativities (*N*) by latency (early: *a*; later: *b*).

Auditory cortex stimulation induced an early VLPFC positivity (*P(a)*: average latency: 5.3 and 6.8 ms, respectively, from stimulating medHG or latHG+PT) followed by an early negativity, *N(a)*, and later positivity and negativity (Figure 6E). Notably, the early positivity is as early as reported from stimulating medial HG and recording in postero-lateral STG (Brugge et al., 2003).

Effects from stimulating VLPFC while recording in HG indicate that connectivity appears to be bidirectional, evident as the presence of both sets of positivities and negativities: compare Figure 6F with stimulation in the opposite direction in Figure 6E. However, the waveforms in the two directions do differ particularly in the latencies of some of the components, which are earlier in the HG to VLPFC direction. An ANOVA (within-subjects factor: potential latency across recordings; between-subjects factors: stimulation sites and subjects) substantiated this impression, showing a significant interaction of stimulation site with potential latency (Greenhouse-Geisser corrected: $F_{1.467,77.742} = 45.641$; $p < 0.001$; $\varepsilon = 0.489$). No other effects or interactions were found.

Next, we studied esT effects between hippocampus and auditory cortex. The waveforms recorded in the hippocampus after stimulation of HG, or vice versa, were also distinctly different in shape, with later components much more variable in latency, depending on the direction of stimulation (compare *N(b)* latencies in Figures 6G and 6H). Also, here, there was a significant interaction of stimulation site with potential latency (Greenhouse-Geisser corrected: $F_{1.407,45.034} = 9.399$; $p < 0.001$; $\varepsilon = 0.469$). No other significant effects or interactions were found.

Testing only the earliest *P(a)* component (excluding the others) showed it to be significantly earlier when stimulating HG and recording in VLPFC than any of the other combinations of directions or stimulation/recording sites (all comparisons relative to stimulating HG and recording in VLPFC; $p < 0.001$; Bonferroni-corrected). Notably, this early latency response in VLPFC resulting from stimulating HG appears as early as the one reported from potentials recorded in the posterior STG when stimulating HG (Brugge et al., 2003) or when recording in VLPFC and stimulating STG (Garell et al., 2013); see Discussion.

Human VLPFC speech responses and directional connectivity with auditory cortex

Lastly, we studied whether speech sounds induce neurophysiological responses in human VLPFC or MTL and the directionality of neurophysiological interactions using state-space conditional Granger causality (CGC). Expectedly, auditory (HG and STG) sites showed strong broad-band speech-driven responses (Figure 7A), including power decreases in low frequencies after speech onset (Billig et al., 2019). Individual contacts showed significant speech responses in PHG and hippocampus, although responses in these areas were weak in the group average results. By comparison, VLPFC responses to speech sounds were substantial in both individual and group results (Figure 7A), evident as increases in lower frequency (theta) power and suppression in alpha and beta bands.

Directional frequency-resolved CGC analyses were conducted between pairs of contacts with the results conditioned on non-specific effects across all contacts during the neurophysiological responses to speech (subject 429; Figure 7B). The expected HG and STG directional interactions were observed (Figure 7B). In this subject, there were no hippocampal contacts, but the available PHG contact only showed significant directional interconnectivity with VLPFC. By comparison, PHG connectivity with auditory cortex was weak. However, VLPFC showed strong bidirectional interactions with the auditory sites in HG and STG (Figure 7B).

DISCUSSION

In monkeys, neuronal tracer studies have shown both direct projections from non-primary auditory cortex to vocalization-sensitive VLPFC neurons (Romanski et al., 1999a, 1999b;

CellPress
OPEN ACCESS

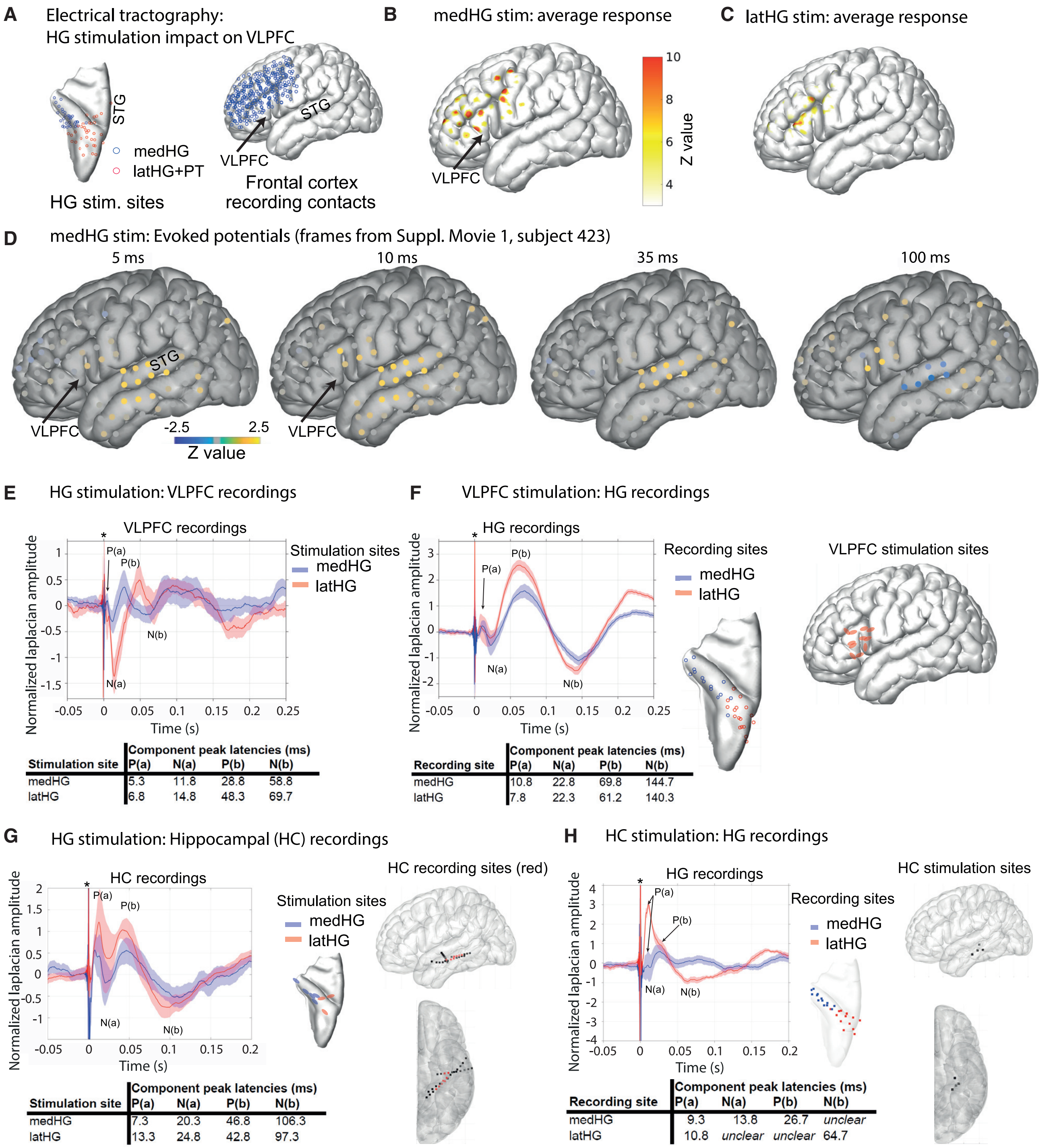

E:

Stimulation site	Component peak latencies (ms) P(a)	N(a)	P(b)	N(b)
medHG	5.3	11.8	28.8	58.8
latHG	6.8	14.8	48.3	69.7

F:

Recording site	Component peak latencies (ms) P(a)	N(a)	P(b)	N(b)
medHG	10.8	22.8	69.8	144.7
latHG	7.8	22.3	61.2	140.3

G:

Stimulation site	Component peak latencies (ms) P(a)	N(a)	P(b)	N(b)
medHG	7.3	20.3	46.8	106.3
latHG	13.3	24.8	42.8	97.3

H:

Recording site	Component peak latencies (ms) P(a)	N(a)	P(b)	N(b)
medHG	9.3	13.8	26.7	*unclear*
latHG	10.8	*unclear*	*unclear*	64.7

Figure 6. Human electrical tractography

(A) Human HG stimulation sites and VLPFC recording electrode locations. Contacts on the right hemisphere were projected onto the left hemisphere.

(B and C) Average evoked response (RMS z-scores, mean and SEM) from the recording contacts shown by medHG (B) or latHG+PT (C) sites of stimulation.

(D) Frames at 5, 10, 35, and 100 ms post-electrical pulse stimulation from Video S1 (subject 423) during medHG stimulation.

(E) Average neurophysiological evoked potentials in VLPFC from stimulating the HG sites, showing the peak latency (ms) for each component. Asterisks at time 0 indicate stimulus artifact.

(F) VLPFC stimulation and recording in HG; same format as in (E); location of stimulation and recording contacts shown on right.

(G) Stimulation of HG during recordings in hippocampus (HC, shown in red in the right panels).

(H) Stimulation of HC during recordings in HG.

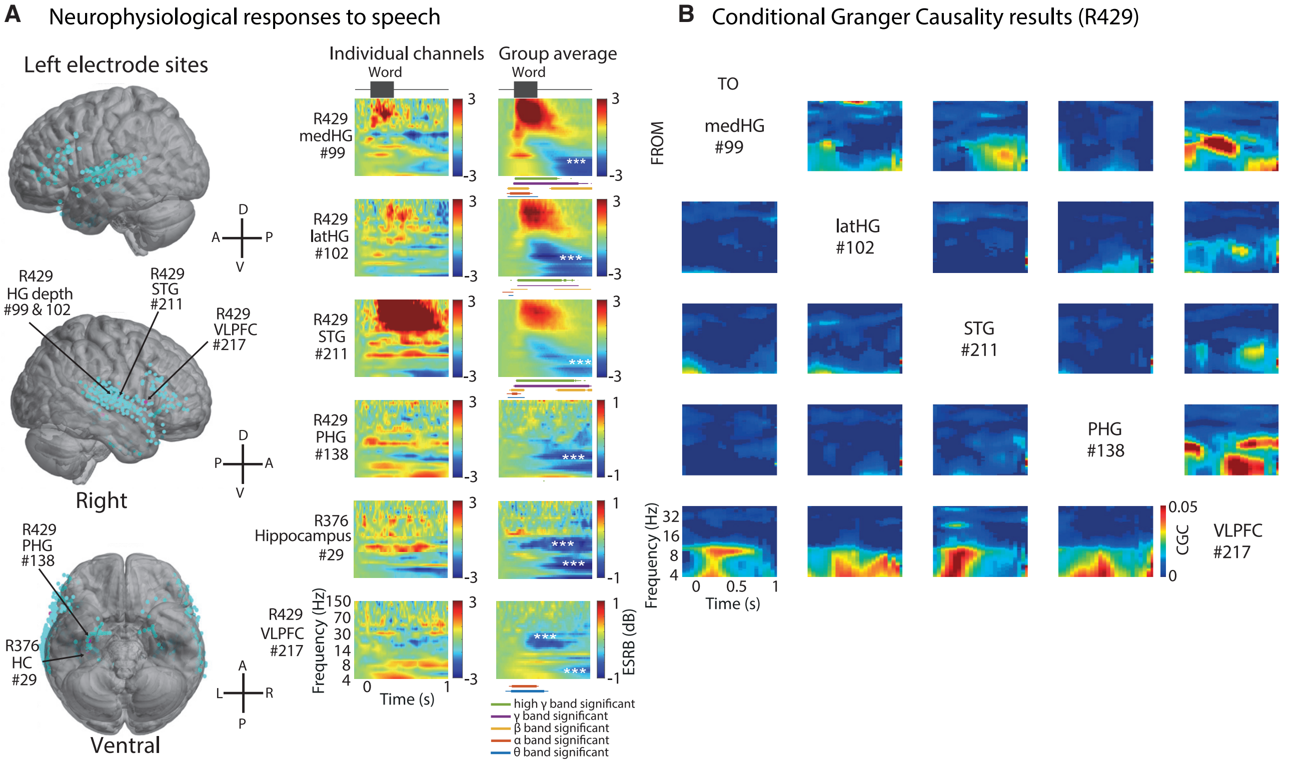

Figure 7. Human VLPFC responses to speech and conditional Granger causality interconnectivity with auditory cortex

(A) Left: electrode locations across all subjects (n = 8), shown as all subjects pooled and projected onto the standard template brain. Right: time-frequency resolved responses to speech sounds (common words) are shown. Shown are single subject individual channels (left column) and group average (right column) within Heschl's gyrus (HG), superior temporal gyrus (STG), PHG, HC, and VLPFC. Subject 429 did not have hippocampal coverage; therefore, the responses from another subject (376) are shown for this region. Below the group results, horizontal bars identify significant responses subdivided by frequency bands (thin > 2 SD; thick > 4 SD relative to the pre-word baseline variability). White *** symbols inset in the group plots identify significant suppression.

(B) State-space conditional Granger causality (CGC) results showing directional neurophysiological interactions during speech-sound presentation. Directions of influence are shown from regions of interest (rows) to recipient regions (columns) active during speech presentation. Subthreshold (not significant) regions of time-frequency CGC were set to 0 and are masked in dark blue. Note the strong dynamic directional influences, particularly between VLPFC and auditory sites, such as HG.

Romanski and Goldman-Rakic, 2002) and indirect projections from auditory cortex to the hippocampus via parahippocampal cortex (Amaral et al., 1983; Insausti and Amaral, 2008; Munoz-Lopez et al., 2010; Muñoz and Insausti, 2005). In humans, because of language and declarative memory, the corresponding pathways are proposed to have specialized in our species (Friederici, 2017; Hagoort, 2019; Schulze et al., 2012). Amidst comparative insights on differentiation involving language pathways (Balezeau et al., 2020; Friederici, 2017; Mars et al., 2018; Neubert et al., 2014), there is surprisingly little evidence for a privileged auditory to VLPFC pathway in humans (Baker et al., 2018a; Garell et al., 2013; Nakae et al., 2020; Neubert et al., 2014), as evident in macaques (Romanski et al., 1999a, 1999b; Romanski and Goldman-Rakic, 2002). Also, human functional connectivity suggests indirect auditory to hippocampal functional connectivity (Baker et al., 2018b; Maller et al., 2019), as in other species, with the only evidence for direct projections between hippocampus proper and hierarchically earlier auditory fields having been obtained in rodents (Cenquizca and Swanson, 2007).

We harnessed a new approach for similarly assessing effective connectivity in humans and monkeys, using es-fMRI (Jones et al., 2014; Klink et al., 2020; Oya et al., 2017; Petkov et al., 2015; Rezai et al., 1999). As a result of auditory cortex stimulation, we found comparable fronto-temporal effective connectivity in both species in several VLPFC (areas 44, 45, and FOP) and MTL subregions. The main species difference seen was in the stronger hemispheric lateralization effects in humans, relative to more bilaterally symmetrical effects in the monkeys. We also provide initial impressions on auditory es-fMRI effects on vocal motor-related areas (Figures S5 and S6), which were, however, less consistent across the analyses with regards to species differences or correspondences (discussed further below). In humans, we also obtained evidence for rapid electrically induced neurophysiological responses in VLPFC from stimulating auditory cortex, with shorter latency in the first measurable potential than that seen in the opposite direction (stimulating VLPFC and recording in the auditory HG), or for interactions between auditory cortex and hippocampus. Moreover, we identified a speech-responsive region in human VLPFC with directional

CellPress
OPEN ACCESS

effective connectivity with auditory cortex. The findings show human auditory pathways that appear to be as direct to VLPFC and indirect to the hippocampus (via parahippocampal cortex) as in nonhuman primates, illuminating the primate origins of fronto-temporal pathways for cognition.

Common auditory es-fMRI effective connectivity to VLPFC and MTL

The comparison of es-fMRI effects in humans and monkeys supports the notion of fronto-temporal networks involving auditory cortex, VLPFC, and MTL having largely evolutionarily conserved effective connectivity signatures. In both species, we observed stronger FOP responses than in VLPFC areas 44 and 45, with some hemispheric difference in humans, evident as a more hemispherically balanced response across these VLPFC regions in the right than left hemisphere. Also, in both species, auditory cortex stimulation induced stronger activation in PHG than other MTL subregions.

The strong FOP effect is interesting, because this region ventral to VLPFC areas 44 and 45 has been implicated in initial syntactic processes in humans, given its involvement in processing local dependencies such as between adjacent words in a sentence (Friederici, 2017). Comparative human and monkey fMRI using a sequence learning task has shown that the FOP, in particular, similarly responds in both species to adjacent sequencing dependencies (Wilson et al., 2015). Also, the FOP is part of a fronto-temporal attentional saliency or task control network that appears to be evolutionarily conserved in primates (Higo et al., 2011).

The comparably stronger auditory cortex es-fMRI effect in monkeys and humans seen in the PHG, in relation to the hippocampal subregions, suggests an equally indirect auditory cortical projection to the hippocampal memory circuit in both species. This observation is not inconsistent with impressions from human resting-state connectivity, including ultra-high-resolution data (Baker et al., 2018a, 2018b). The current findings challenge the notion that monkeys have less direct auditory effective interconnectivity with the MTL memory system than humans. Our language abilities allow us to name, conceptualize, and thus better remember sounds (Schulze et al., 2012). Yet, as our observations suggest, possibly even these language-related mnemonic functions are instantiated within a largely evolutionarily conserved MTL system.

The mode of es-fMRI action is incompletely understood. A prior monkey es-fMRI study showed that electrical stimulation of the visual thalamus elicits an fMRI response in primary visual cortex (V1) with inhibitory neurons reducing signal propagation to other areas (Logothetis et al., 2010). However, some propagation to multi-synaptically connected sites appears to be evident (Klink et al., 2020), which we also see in our results in the form of graded es-fMRI responses. Namely, the graded es-fMRI pattern across MTL ROIs is broadly consistent with known parahippocampal and hippocampal subregion connectivity (Jinno, 2009; Witter, 1993).

Electrical stimulation effects can, in principle, result from both antidromic and orthodromic propagation along axons. A comparison of electrical stimulation effects on feedforward and feedback influences between visual areas V1 and V4 indicates that orthodromic influences tend to be stronger and less stereotyped in either direction (Klink et al., 2017). For instance, seeing asymmetry in directional effects between two sites suggests primarily orthodromic influences. This is precisely what we observed in the asymmetry of the electrical tractography effects between auditory cortex and VLPFC (discussed further below).

Cross-species differences in es-fMRI effects

Evidence for human differentiation was clearest in the form of hemispheric lateralization. By contrast, the monkey es-fMRI effects were largely bilateral, even though it was only possible to stimulate the right hemisphere in the monkeys. The human results showed some hemispheric lateralization effects in VLPFC and MTL subregions.

There were also differences in effects between humans and monkeys by auditory cortical site of stimulation. We initially stimulated auditory core and belt areas in the monkeys, aiming to identify distinctly different es-fMRI effects, given that belt neurons are known to project to prefrontal cortex (Diehl and Romanski, 2013). However, the monkey results were statistically indistinguishable when comparing effects from stimulating these two auditory sites adjacent to one another, potentially because of passive current spread estimated to be ~1-mm radius around the stimulating electrode (Results). The human results were distinctly different between the two auditory stimulation sites, one of which was identified by its auditory click following response (Brugge et al., 2008). Passive current spread in the human results is also worth considering, estimated to be 3-mm radius around the stimulating contacts. Thereby, some of the human medHG effects can involve stimulation of adjacent areas.

To illustrate the power of the approach for the study of brain systems beyond the ones that we focused on in this paper involving the defined VLPFC and MTL areas, we also conducted an analysis of auditory effective connectivity to areas associated with vocal motor production (Rauschecker and Scott, 2009). There are evolutionary hypotheses on both the role of these areas for auditory-motor mapping and how the vocal production system may have differentiated in humans relative to this system in other primates and mammals (Archakov et al., 2020; Arriaga et al., 2012; Simonyan and Horwitz, 2011). There is also indication of convergent evolution in humans and songbirds (Petkov and Jarvis, 2012; Roberts et al., 2017). For instance, human laryngeal motor cortex (LMC) resides within M1 and has direct projections to the nucleus ambiguous in the brainstem, which controls laryngeal muscles for vocal production (Jürgens, 2002; Simonyan and Horwitz, 2011). By comparison, the primate evolutionary precursor of LMC appears to reside not necessarily in M1 but in parts of areas 6v and 44 that when electrically stimulated result in contraction of the laryngeal muscles (Petrides et al., 2005; Simonyan and Horwitz, 2011). Moreover, the pathway from these primate areas to the nucleus ambiguous in monkeys does not appear to be direct, although this remains under active study (Archakov et al., 2020; Boë et al., 2019; Dichter et al., 2018). Our classifier results (Figure S6) showed some potentially interesting findings in line with this evolutionary hypothesis (e.g., the classifier could detect the species differences in areas M1 and 6v among other areas, but not in area 6d, for example). However, the classifier results were not consistent

with the ANOVA results, which did not identify a species difference. Thus, this issue requires further es-fMRI study alongside functional localization of these frontal areas using electrical stimulation that results in laryngeal muscle contraction. Our initial impressions alongside establishing the comparative es-fMRI approach establish a foundation for future work on this system.

Other systems are also now worth exploring with the comparative es-fMRI approach. In this regard, Table S1 shows that there can be strong effects in a number of other brain areas, such as visual cortical areas and those associated with multisensory integration (Beauchamp et al., 2004; Petkov et al., 2015). Pathways between auditory and visual cortex for multisensory neural interaction are established, which may be why there is visual cortical involvement when auditory cortex is stimulated, as also seen elsewhere (Petkov et al., 2015). Affective pathways between auditory cortex and the amygdala (Choi et al., 2010) are also worth exploring with the comparative es-fMRI approach. To support open data sharing and further discovery, particularly for systems beyond the scope of this paper or if parcellation schemes are updated, the macaque data are shared via the Open Science Framework and the PRIMatE Data Exchange (PRIME-DE) (PRIMatE Data Exchange (PRIME-DE) Global Collaboration Workshop and Consortium, 2020; Milham et al., 2018). The human data are shared via the Open Science Framework and the recently established human es-fMRI resource in OpenNeuro (Thompson et al., 2020); links to the resources are found in the STAR methods table. There is also detailed information on safely implementing the approach with both species (Klink et al., 2020; Oya et al., 2017) including at the PRIMatE Research Exchange under the topic "electrical stimulation and neuroimaging: humans and macaques" (link in STAR methods table).

Considerations in relation to white matter pathways and monkey anterograde tracer studies

The effective connectivity approach we used is agnostic to the white matter tracts that interconnect auditory cortex with the fronto-temporal sites. Human diffusion MRI and monkey anterograde tracer studies indicate that the FOP and parts of area 45 (45A; the more anterior segment in macaques; Frey et al., 2014; Gerbella et al., 2010) are interconnected with auditory cortex via the ventral extreme capsule or uncinate fasciculus pathways (Rilling et al., 2008). Area 44 in both species is interconnected with areas caudal to auditory cortex via the dorsal arcuate fasciculus pathway (Catani et al., 2005; Frey et al., 2008). A recent comparative study showed an auditory homolog of this dorsal pathway in macaques (Balezeau et al., 2020), which interconnects caudo-medial auditory regions—near to those that we electrically stimulated—with VLPFC.

Is it unexpected that our es-fMRI results from stimulating posterior auditory cortical sites show effects in VLPFC? Monkey anterograde neuronal tracing results from a caudal auditory belt area (CL) show dense *dorso-lateral* prefrontal cortex axonal bouton labeling (Romanski et al., 1999a, 1999b; also see Hackett et al., 1999; Petrides and Pandya, 1988, 2002). Although mesoscopic effects, such as those measured by es-fMRI, lack the specificity of microscopic neuronal tracer studies, our results recapitulate key patterns shown with macaque anterograde tracing results, which show both anterior and posterior auditory belt neuronal tracer injections labeling axonal boutons in VLPFC (Romanski et al., 1999b). Also, in relation to prior macaque es-fMRI work, our results are more like those reported from stimulating posterior lateral belt (field ML) than those from stimulating the anterior lateral belt (field RTL; Petkov et al., 2015). In that study, there was one auditory field separation between the two stimulated sites. In our study, there was no such buffer region between the two stimulated sites, which may be why our effects from stimulating the two sites were statistically indistinguishable. Another consideration is the inherent differences in how the monkeys and humans were studied (e.g., different scanners, electrodes, and parameters). However, amidst all such differences, it is notable how similar the es-fMRI effects in VLPFC and MTL were across the species.

Rapid human electrical tractography from auditory cortex to VLPFC

The study provides evidence for a rapid electrical-tractography response in VLPFC following HG stimulation. An early positive potential occurred in VLPFC as soon as we could measure it after the stimulation artifact, peaking ~5–7 ms after HG stimulation. Auditory cortex and the VLPFC are separated by ~10 cm via the arcuate fasciculus. The latency of this early positivity was significantly longer in the opposite direction (stimulating VLPFC and recording in HG) and between HG and the hippocampus in either direction (Figures 6E–6H). The estimated conduction velocity, based on axonal diameter and conduction speeds (Bishop and Smith, 1964; Caminiti et al., 2013), including within fronto-temporal pathways (Greenlee et al., 2004), is 10–30 m/s. Thus, the expected latency of the first potentials from auditory cortex arriving in VLPFC is between 3 and 10 ms. Our HG to VLPFC latency results are remarkably similar to the reported ~6-ms peak latency of the initial positivity recorded in the posterior auditory STG after medial HG stimulation (Brugge et al., 2003); the two sites are separated by 1 to 2 cm. The authors in that study could not exclude the possibility of a monosynaptic connection between medial HG and the STG, because surprisingly, stimulation of a lateral HG site expected to be an intermediary elicited weak recorded potentials in the STG. In another study, stimulation of the posterior STG while recording in VLPFC resulted in an early negativity (the study focused on negative potentials) with an average VLPFC response latency of ~13.5 ms (Garell et al., 2013), which is similar to our first negativity latency in VLPFC after stimulating HG (Figure 6E). Even longer first negativity latencies can be measured in VLPFC after stimulating other temporal lobe sites (Nakae et al., 2020).

Thereby, our early electrical tractography potential latencies from HG to VLPFC appear similar to those reported from stimulating STG while recording in VLPFC (Garell et al., 2013). The results raise the possibility that the HG to VLPFC connection is as rapid as the one from posterior STG to VLPFC. Although we cannot exclude multi-synaptic effects, it is possible that, if medial or lateral HG sites require additional synaptic connections in the STG, the VLPFC early positivity (or negativity) would peak later. Moreover, although HG and VLPFC effective connectivities generally appear to be bidirectional, there were key differences in the shape of waveforms in either direction, and the early positivity was earlier when stimulating HG and recording in VLPFC than in the opposite direction. Also, the early positive

CellPress
OPEN ACCESS

potential between HG and hippocampus, in either direction, was later than the one observed between HG and VLPFC. Our latencies between HG and the hippocampus are in the range of those reported from recording in various temporal lobe sites after entorhinal cortex stimulation (Takeyama et al., 2019). The findings provide support for privileged auditory HG to VLPFC effective connectivity that fundamentally differs from effects in the opposite direction and from those involving the hippocampus. In comparison to the rapid electrical tractography effects from auditory cortex to VLPFC, we hypothesize that there might be more synaptic stages in the feedback pathway from VLPFC to auditory cortex and in both directions between auditory cortex and the hippocampus.

Human-speech-responsive VLPFC with directional auditory cortex effective connectivity

We observed considerable speech-sound-driven neurophysiological responses in human VLPFC and show conditional Granger causality results on bidirectional effective connectivity between HG and VLPFC. Speech responses in VLPFC are not unexpected. They are typically evident during active speech recognition or difficult listening conditions (Davis et al., 2007). Spoken speech or reading can also elicit responses from the human hippocampus (Jafarpour et al., 2017; Figure 7). The conditional Granger causality results, showing interconnectivity between VLPFC and HG during speech-sound processing, provides further evidence for privileged auditory to VLPFC interconnectivity in the human brain.

In summary, the findings demonstrate largely comparable effective connectivity signatures between human and macaque auditory cortex and the studied VLPFC and MTL areas. The auditory system as the model sensory system under study was expected to show substantial specialization in humans for speech, language, and declarative memory. However, even these results involving auditory cortex identify a common principle in fronto-temporal effective connectivity across these species. Future studies in other sensory modalities and species could further support or refute these observations.

STAR★METHODS

Detailed methods are provided in the online version of this paper and include the following:

- KEY RESOURCES TABLE
- RESOURCE AVAILABILITY
  - Lead contact
  - Materials availability
  - Data and code availability
- EXPERIMENTAL MODEL AND SUBJECT DETAILS
  - Macaque subjects
  - Human subjects
- METHOD DETAILS
  - Macaque es-fMRI procedure
  - Macaque es-fMRI processing
  - Human es-fMRI procedure
  - Stimulation site categorization procedure
  - Electrode localization procedure
  - Human es-fMRI data processing
  - Anatomical image preprocessing
  - Functional data preprocessing
  - Human electrical stimulation tract-tracing (esT)
  - Spline-Laplacian correction
  - esT movie creation method
  - Speech sound recording experiment and processing
  - Speech stimuli
  - Event-related spectral LFP decomposition
- QUANTIFICATION AND STATISTICAL ANALYSIS
  - Macaque quantification and statistical analysis
  - Human quantification and statistical analysis
  - Catboost classification analysis
  - Vocal motor-related analyses
  - State-space Conditional Granger Causality (CGC) analysis

SUPPLEMENTAL INFORMATION

Supplemental Information can be found online at https://doi.org/10.1016/j.neuron.2020.12.026.

A video abstract is available at https://doi.org/10.1016/j.neuron.2020.12.026#mmc5.

ACKNOWLEDGMENTS

We acknowledge the contribution of the neurosurgery patients and the nonhuman primates in this work. For the human work, we thank H. Chen and P.E. Gander for es-fMRI experiment assistance, M.A. Granner for safety monitoring, V. Magnotta for technical consultation, and J.M. Buatti for help with MRI logistics. For the macaque work, we thank J. Nacef and the Comparative Biology Centre animal technician and veterinary teams. We thank M.I. Banks and P.E. Gander for useful discussion. This work was supported by Wellcome Trust (WT092606AIA; to C.I.P.), European Research Council Horizon 2020 (ERC CoG, Consolidator Grant, MECHIDENT 724198; to C.I.P.), and National Institutes of Health (R01 DC004290-20 to M.A.H.; U01 NS103780-03 to R.A.). The human work was conducted on an MRI instrument funded by National Institutes of Health grant 1S10OD025025-01.

AUTHOR CONTRIBUTIONS

Conceptualization and Project Administration, C.I.P., M.A.H., and T.D.G.; Investigation and Formal Analysis, F.R., H.O., Z.K., R.L.J., and C.I.P.; Methodology and Materials, F.R., H.O., F.B., Z.K., R.L.J., K.V.N., C.K.K., M.S., Y.K., A.E.R., B.J.D., H.K., J.D.W.G., M.A.H., and C.I.P.; Writing – Original Draft, C.I.P., F.R., and H.O.; Writing – Review and Editing, C.I.P., F.R., H.O., F.B., A.J.B., Z.K., R.L.J., K.V.N., Y.K., A.E.R., B.J.D., H.K., R.A., J.D.W.G., T.D.G., and M.A.H.; Funding Acquisition, C.I.P., M.A.H., T.D.G., R.A., and J.D.W.G.; Data Curation and Resources, F.R., H.O., and C.I.P.

DECLARATION OF INTERESTS

The authors declare no competing interests.

Received: April 24, 2020
Revised: October 2, 2020
Accepted: December 30, 2020
Published: January 21, 2021

REFERENCES

Amaral, D.G., Insausti, R., and Cowan, W.M. (1983). Evidence for a direct projection from the superior temporal gyrus to the entorhinal cortex in the monkey. Brain Res. *275*, 263–277.

Amunts, K., and Zilles, K. (2015). Architectonic mapping of the human brain beyond Brodmann. Neuron *88*, 1086–1107.

Archakov, D., DeWitt, I., Kuśmierek, P., Ortiz-Rios, M., Cameron, D., Cui, D., Morin, E.L., VanMeter, J.W., Sams, M., Jääskeläinen, I.P., and Rauschecker, J.P. (2020). Auditory representation of learned sound sequences in motor regions of the macaque brain. Proc. Natl. Acad. Sci. USA *117*, 15242–15252.

Arriaga, G., Zhou, E.P., and Jarvis, E.D. (2012). Of mice, birds, and men: the mouse ultrasonic song system has some features similar to humans and song-learning birds. PLoS ONE *7*, e46610.

Baker, C.M., Burks, J.D., Briggs, R.G., Conner, A.K., Glenn, C.A., Robbins, J.M., Sheets, J.R., Sali, G., McCoy, T.M., Battiste, J.D., et al. (2018a). A connectomic atlas of the human cerebrum—chapter 5: the insula and opercular cortex. Oper. Neurosurg. *15*, S175–S244.

Baker, C.M., Burks, J.D., Briggs, R.G., Milton, C.K., Conner, A.K., Glenn, C.A., Sali, G., McCoy, T.M., Battiste, J.D., O'Donoghue, D.L., et al. (2018b). A connectomic atlas of the human cerebrum—chapter 6: the temporal lobe. Oper. Neurosurg. *15*, S245–S294.

Balezeau, F., Wilson, B., Gallardo, G., Dick, F., Hopkins, W., Anwander, A., Friederici, A.D., Griffiths, T.D., and Petkov, C.I. (2020). Primate auditory prototype in the evolution of the arcuate fasciculus. Nat. Neurosci. *23*, 611–614.

Barnett, L., and Seth, A.K. (2015). Granger causality for state-space models. Phys. Rev. E Stat. Nonlin. Soft Matter Phys. *91*, 040101.

Barnett, L., Barrett, A.B., and Seth, A.K. (2018). Solved problems for Granger causality in neuroscience: A response to Stokes and Purdon. Neuroimage *178*, 744–748.

Baumann, S., Griffiths, T.D., Rees, A., Hunter, D., Sun, L., and Thiele, A. (2010). Characterisation of the BOLD response time course at different levels of the auditory pathway in non-human primates. Neuroimage *50*, 1099–1108.

Beauchamp, M.S., Argall, B.D., Bodurka, J., Duyn, J.H., and Martin, A. (2004). Unraveling multisensory integration: patchy organization within human STS multisensory cortex. Nat. Neurosci. *7*, 1190–1192.

Becker, C.O., Bassett, D.S., and Preciado, V.M. (2018). Large-scale dynamic modeling of task-fMRI signals via subspace system identification. J. Neural Eng. *15*, 066016.

Belyk, M., and Brown, S. (2017). The origins of the vocal brain in humans. Neurosci. Biobehav. Rev. *77*, 177–193.

Billig, A.J., Herrmann, B., Rhone, A.E., Gander, P.E., Nourski, K.V., Snoad, B.F., Kovach, C.K., Kawasaki, H., Howard, M.A., 3rd, and Johnsrude, I.S. (2019). A sound-sensitive source of alpha oscillations in human non-primary auditory cortex. J. Neurosci. *39*, 8679–8689.

Bishop, G.H., and Smith, J.M. (1964). The sizes of nerve fibers supplying cerebral cortex. Exp. Neurol. *9*, 483–501.

Boë, L.-J., Sawallis, T.R., Fagot, J., Badin, P., Barbier, G., Captier, G., Ménard, L., Heim, J.-L., and Schwartz, J.-L. (2019). Which way to the dawn of speech?: reanalyzing half a century of debates and data in light of speech science. Sci. Adv. *5*, eaaw3916.

Boutet, A., Rashid, T., Hancu, I., Elias, G.J.B., Gramer, R.M., Germann, J., Dimarzio, M., Li, B., Paramanandam, V., Prasad, S., et al. (2019). Functional MRI safety and artifacts during deep brain stimulation: experience in 102 patients. Radiology *293*, 174–183.

Brugge, J.F., Volkov, I.O., Garell, P.C., Reale, R.A., and Howard, M.A., 3rd (2003). Functional connections between auditory cortex on Heschl's gyrus and on the lateral superior temporal gyrus in humans. J. Neurophysiol. *90*, 3750–3763.

Brugge, J.F., Volkov, I.O., Oya, H., Kawasaki, H., Reale, R.A., Fenoy, A., Steinschneider, M., and Howard, M.A., 3rd (2008). Functional localization of auditory cortical fields of human: click-train stimulation. Hear. Res. *238*, 12–24.

Caminiti, R., Carducci, F., Piervincenzi, C., Battaglia-Mayer, A., Confalone, G., Visco-Comandini, F., Pantano, P., and Innocenti, G.M. (2013). Diameter, length, speed, and conduction delay of callosal axons in macaque monkeys and humans: comparing data from histology and magnetic resonance imaging diffusion tractography. J. Neurosci. *33*, 14501–14511.

Carvalhaes, C., and de Barros, J.A. (2015). The surface Laplacian technique in EEG: theory and methods. Int. J. Psychophysiol. *97*, 174–188.

Catani, M., Jones, D.K., and ffytche, D.H. (2005). Perisylvian language networks of the human brain. Ann. Neurol. *57*, 8–16.

Cenquizca, L.A., and Swanson, L.W. (2007). Spatial organization of direct hippocampal field CA1 axonal projections to the rest of the cerebral cortex. Brain Res. Brain Res. Rev. *56*, 1–26.

Choi, J.-S., Cain, C.K., and LeDoux, J.E. (2010). The role of amygdala nuclei in the expression of auditory signaled two-way active avoidance in rats. Learn. Mem. *17*, 139–147.

Cox, R.W. (1996). AFNI: software for analysis and visualization of functional magnetic resonance neuroimages. Comput. Biomed. Res. *29*, 162–173.

Da Costa, S., van der Zwaag, W., Marques, J.P., Frackowiak, R.S., Clarke, S., and Saenz, M. (2011). Human primary auditory cortex follows the shape of Heschl's gyrus. J. Neurosci. *31*, 14067–14075.

Davis, M.H., Coleman, M.R., Absalom, A.R., Rodd, J.M., Johnsrude, I.S., Matta, B.F., Owen, A.M., and Menon, D.K. (2007). Dissociating speech perception and comprehension at reduced levels of awareness. Proc. Natl. Acad. Sci. USA *104*, 16032–16037.

Dichter, B.K., Breshears, J.D., Leonard, M.K., and Chang, E.F. (2018). The control of vocal pitch in human laryngeal motor cortex. Cell *174*, 21–31.e9.

Diehl, M., and Romanski, L. (2013). Representation and integration of faces and vocalizations in the primate ventral prefrontal cortex. In Integrating Face and Voice in Person Perception, P. Belin, S. Campanella, and T. Ethofer, eds. (Springer), pp. 45–69.

Donahue, C.J., Glasser, M.F., Preuss, T.M., Rilling, J.K., and Van Essen, D.C. (2018). Quantitative assessment of prefrontal cortex in humans relative to nonhuman primates. Proc. Natl. Acad. Sci. USA *115*, E5183–E5192.

Eichert, N., Verhagen, L., Folloni, D., Jbabdi, S., Khrapitchev, A.A., Sibson, N.R., Mantini, D., Sallet, J., and Mars, R.B. (2019). What is special about the human arcuate fasciculus? Lateralization, projections, and expansion. Cortex *118*, 107–115.

Eickhoff, S.B., Stephan, K.E., Mohlberg, H., Grefkes, C., Fink, G.R., Amunts, K., and Zilles, K. (2005). A new SPM toolbox for combining probabilistic cytoarchitectonic maps and functional imaging data. Neuroimage *25*, 1325–1335.

Ekstrom, L.B., Roelfsema, P.R., Arsenault, J.T., Bonmassar, G., and Vanduffel, W. (2008). Bottom-up dependent gating of frontal signals in early visual cortex. Science *321*, 414–417.

Esteban, O., Markiewicz, C.J., Blair, R.W., Moodie, C.A., Isik, A.I., Erramuzpe, A., Kent, J.D., Goncalves, M., DuPre, E., Snyder, M., et al. (2019). fMRIPrep: a robust preprocessing pipeline for functional MRI. Nat. Methods *16*, 111–116.

Fischl, B. (2012). FreeSurfer. Neuroimage *62*, 774–781.

Flinker, A., and Knight, R.T. (2018). Broca's area in comprehension and production, insights from intracranial studies in humans. Curr. Opin. Behav. Sci. *21*, 170–175.

Frey, S., Campbell, J.S., Pike, G.B., and Petrides, M. (2008). Dissociating the human language pathways with high angular resolution diffusion fiber tractography. J. Neurosci. *28*, 11435–11444.

Frey, S., Mackey, S., and Petrides, M. (2014). Cortico-cortical connections of areas 44 and 45B in the macaque monkey. Brain Lang. *131*, 36–55.

Friederici, A.D. (2017). Language in Our Brain: The Origins of a Uniquely Human Capacity (MIT).

Fritz, J., Mishkin, M., and Saunders, R.C. (2005). In search of an auditory engram. Proc. Natl. Acad. Sci. USA *102*, 9359–9364.

Garell, P.C., Bakken, H., Greenlee, J.D., Volkov, I., Reale, R.A., Oya, H., Kawasaki, H., Howard, M.A., and Brugge, J.F. (2013). Functional connection between posterior superior temporal gyrus and ventrolateral prefrontal cortex in human. Cereb. Cortex *23*, 2309–2321.

Gerbella, M., Belmalih, A., Borra, E., Rozzi, S., and Luppino, G. (2010). Cortical connections of the macaque caudal ventrolateral prefrontal areas 45A and 45B. Cereb. Cortex *20*, 141–168.

CellPress
OPEN ACCESS

Geweke, J. (1982). Measurement of linear dependence and feedback between multiple time series. J. Am. Stat. Assoc. *77*, 304–313.

Geweke, J.F. (1984). Measures of conditional linear dependence and feedback between time series. J. Am. Stat. Assoc. *79*, 907–915.

Greenlee, J.D., Oya, H., Kawasaki, H., Volkov, I.O., Kaufman, O.P., Kovach, C., Howard, M.A., and Brugge, J.F. (2004). A functional connection between inferior frontal gyrus and orofacial motor cortex in human. J. Neurophysiol. *92*, 1153–1164.

Hackett, T.A., Stepniewska, I., and Kaas, J.H. (1999). Prefrontal connections of the parabelt auditory cortex in macaque monkeys. Brain Res. *817*, 45–58.

Hagoort, P. (2019). The neurobiology of language beyond single-word processing. Science *366*, 55–58.

Hall, D.A., Haggard, M.P., Akeroyd, M.A., Palmer, A.R., Summerfield, A.Q., Elliott, M.R., Gurney, E.M., and Bowtell, R.W. (1999). ‘‘Sparse’’ temporal sampling in auditory fMRI. Hum. Brain Mapp. *7*, 213–223.

Higo, T., Mars, R.B., Boorman, E.D., Buch, E.R., and Rushworth, M.F.S. (2011). Distributed and causal influence of frontal operculum in task control. Proc. Natl. Acad. Sci. USA *108*, 4230–4235.

Hutchison, R.M., Gallivan, J.P., Culham, J.C., Gati, J.S., Menon, R.S., and Everling, S. (2012). Functional connectivity of the frontal eye fields in humans and macaque monkeys investigated with resting-state fMRI. J. Neurophysiol. *107*, 2463–2474.

Iglesias, J.E., and Sabuncu, M.R. (2015). Multi-atlas segmentation of biomedical images: A survey. Med. Image Anal. *24*, 205–219.

Insausti, R., and Amaral, D.G. (2008). Entorhinal cortex of the monkey: IV. topographical and laminar organization of cortical afferents. J. Comp. Neurol. *509*, 608–641.

Jafarpour, A., Piai, V., Lin, J.J., and Knight, R.T. (2017). Human hippocampal pre-activation predicts behavior. Sci. Rep. *7*, 5959.

Jarvis, E.D. (2019). Evolution of vocal learning and spoken language. Science *366*, 50–54.

Jenison, R.L. (2014). Directional influence between the human amygdala and orbitofrontal cortex at the time of decision-making. PLoS ONE *9*, e109689.

Jinno, S. (2009). Structural organization of long-range GABAergic projection system of the hippocampus. Front. Neuroanat. *3*, 13.

Jones, S.E., Zhang, M., Avitsian, R., Bhattacharyya, P., Bulacio, J., Cendes, F., Enatsu, R., Lowe, M., Najm, I., Nair, D., et al. (2014). Functional magnetic resonance imaging networks induced by intracranial stimulation may help defining the epileptogenic zone. Brain Connect. *4*, 286–298.

Jürgens, U. (2002). Neural pathways underlying vocal control. Neurosci. Biobehav. Rev. *26*, 235–258.

Kayser, J., and Tenke, C.E. (2015). On the benefits of using surface Laplacian (current source density) methodology in electrophysiology. Int. J. Psychophysiol. *97*, 171–173.

Klink, C., Aubry, J.-F., Ferrera, V., Fox, A.S., Froudist-Walsh, S., Jarraya, B., Konofagou, E., Krauzlis, R., Messinger, A., Mitchell, A.S., et al. (2020). Combined brain perturbation and neuroimaging in non-human primates. OSF Preprints. https://doi.org/10.31219/osf.io/z4x89.

Klink, P.C., Dagnino, B., Gariel-Mathis, M.-A., and Roelfsema, P.R. (2017). Distinct feedforward and feedback effects of microstimulation in visual cortex reveal neural mechanisms of texture segregation. Neuron *95*, 209–220.e3.

Kovach, C.K., and Gander, P.E. (2016). The demodulated band transform. J. Neurosci. Methods *261*, 135–154.

Koyama, M., Hasegawa, I., Osada, T., Adachi, Y., Nakahara, K., and Miyashita, Y. (2004). Functional magnetic resonance imaging of macaque monkeys performing visually guided saccade tasks: comparison of cortical eye fields with humans. Neuron *41*, 795–807.

Lancaster, G., Iatsenko, D., Pidde, A., Ticcinelli, V., and Stefanovska, A. (2018). Surrogate data for hypothesis testing of physical systems. Phys. Rep. *748*, 1–60.

Lemon, R.N. (2008). Descending pathways in motor control. Annu. Rev. Neurosci. *31*, 195–218.

Lobel, E., Kahane, P., Leonards, U., Grosbras, M., Lehéricy, S., Le Bihan, D., and Berthoz, A. (2001). Localization of human frontal eye fields: anatomical and functional findings of functional magnetic resonance imaging and intracerebral electrical stimulation. J. Neurosurg. *95*, 804–815.

Logothetis, N.K., Augath, M., Murayama, Y., Rauch, A., Sultan, F., Goense, J., Oeltermann, A., and Merkle, H. (2010). The effects of electrical microstimulation on cortical signal propagation. Nat. Neurosci. *13*, 1283–1291.

Maller, J.J., Welton, T., Middione, M., Callaghan, F.M., Rosenfeld, J.V., and Grieve, S.M. (2019). Revealing the hippocampal connectome through super-resolution 1150-direction diffusion MRI. Sci. Rep. *9*, 2418.

Mars, R.B., Eichert, N., Jbabdi, S., Verhagen, L., and Rushworth, M.F.S. (2018). Connectivity and the search for specializations in the language-capable brain. Curr. Opin. Behav. Sci. *21*, 19–26.

McLaren, D.G., Kosmatka, K.J., Oakes, T.R., Kroenke, C.D., Kohama, S.G., Matochik, J.A., Ingram, D.K., and Johnson, S.C. (2009). A population-average MRI-based atlas collection of the rhesus macaque. Neuroimage *45*, 52–59.

Milham, M.P., Ai, L., Koo, B., Xu, T., Amiez, C., Balezeau, F., Baxter, M.G., Blezer, E.L.A., Brochier, T., Chen, A., et al. (2018). An open resource for non-human primate imaging. Neuron *100*, 61–74.e2.

PRIMatE Data Exchange (PRIME-DE) Global Collaboration Workshop and Consortium (2020). Accelerating the evolution of nonhuman primate neuroimaging. Neuron *105*, 600–603.

Moeller, S., Freiwald, W.A., and Tsao, D.Y. (2008). Patches with links: a unified system for processing faces in the macaque temporal lobe. Science *320*, 1355–1359.

Muñoz, M., and Insausti, R. (2005). Cortical efferents of the entorhinal cortex and the adjacent parahippocampal region in the monkey (Macaca fascicularis). Eur. J. Neurosci. *22*, 1368–1388.

Munoz-Lopez, M.M., Mohedano-Moriano, A., and Insausti, R. (2010). Anatomical pathways for auditory memory in primates. Front. Neuroanat. *4*, 129.

Nakae, T., Matsumoto, R., Kunieda, T., Arakawa, Y., Kobayashi, K., Shimotake, A., Yamao, Y., Kikuchi, T., Aso, T., Matsuhashi, M., et al. (2020). Connectivity gradient in the human left inferior frontal gyrus: intraoperative cortico-cortical evoked potential study. Cereb. Cortex *30*, 4633–4650.

Neubert, F.X., Mars, R.B., Thomas, A.G., Sallet, J., and Rushworth, M.F. (2014). Comparison of human ventral frontal cortex areas for cognitive control and language with areas in monkey frontal cortex. Neuron *81*, 700–713.

Nourski, K.V., Brugge, J.F., Reale, R.A., Kovach, C.K., Oya, H., Kawasaki, H., Jenison, R.L., and Howard, M.A., 3rd (2013). Coding of repetitive transients by auditory cortex on posterolateral superior temporal gyrus in humans: an intracranial electrophysiology study. J. Neurophysiol. *109*, 1283–1295.

Nourski, K.V., Steinschneider, M., Rhone, A.E., and Howard Iii, M.A. (2017). Intracranial electrophysiology of auditory selective attention associated with speech classification tasks. Front. Hum. Neurosci. *10*, 691.

Nunez, P.L., and Pilgreen, K.L. (1991). The spline-Laplacian in clinical neurophysiology: a method to improve EEG spatial resolution. J. Clin. Neurophysiol. *8*, 397–413.

Oya, H., Howard, M.A., Magnotta, V.A., Kruger, A., Griffiths, T.D., Lemieux, L., Carmichael, D.W., Petkov, C.I., Kawasaki, H., Kovach, C.K., et al. (2017). Mapping effective connectivity in the human brain with concurrent intracranial electrical stimulation and BOLD-fMRI. J. Neurosci. Methods *277*, 101–112.

Oya, H., Gander, P.E., Petkov, C.I., Adolphs, R., Nourski, K.V., Kawasaki, H., Howard, M.A., and Griffiths, T.D. (2018). Neural phase locking predicts BOLD response in human auditory cortex. Neuroimage *169*, 286–301.

Perrin, F., Pernier, J., Bertrand, O., and Echallier, J.F. (1989). Spherical splines for scalp potential and current density mapping. Electroencephalogr. Clin. Neurophysiol. *72*, 184–187.

Petit, L., and Pouget, P. (2019). The comparative anatomy of frontal eye fields in primates. Cortex *118*, 51–64.

Petkov, C.I., and Jarvis, E.D. (2012). Birds, primates, and spoken language origins: behavioral phenotypes and neurobiological substrates. Front. Evol. Neurosci. *4*, 12.

Petkov, C.I., Kayser, C., Augath, M., and Logothetis, N.K. (2009). Optimizing the imaging of the monkey auditory cortex: sparse vs. continuous fMRI. Magn. Reson. Imaging *27*, 1065–1073.

Petkov, C.I., Kikuchi, Y., Milne, A.E., Mishkin, M., Rauschecker, J.P., and Logothetis, N.K. (2015). Different forms of effective connectivity in primate frontotemporal pathways. Nat. Commun. *6*, 6000.

Petrides, M., and Pandya, D.N. (1988). Association fiber pathways to the frontal cortex from the superior temporal region in the rhesus monkey. J. Comp. Neurol. *273*, 52–66.

Petrides, M., and Pandya, D.N. (2002). Comparative cytoarchitectonic analysis of the human and the macaque ventrolateral prefrontal cortex and corticocortical connection patterns in the monkey. Eur. J. Neurosci. *16*, 291–310.

Petrides, M., Cadoret, G., and Mackey, S. (2005). Orofacial somatomotor responses in the macaque monkey homologue of Broca's area. Nature *435*, 1235–1238.

Phillips, M.D., Baker, K.B., Lowe, M.J., Tkach, J.A., Cooper, S.E., Kopell, B.H., and Rezai, A.R. (2006). Parkinson disease: pattern of functional MR imaging activation during deep brain stimulation of subthalamic nucleus–initial experience. Radiology *239*, 209–216.

Prichard, D., and Theiler, J. (1994). Generating surrogate data for time series with several simultaneously measured variables. Phys. Rev. Lett. *73*, 951–954.

Prokhorenkova, L., Gusev, G., Vorobev, A., Dorogush, A.V., and Gulin, A. (2017). CatBoost: unbiased boosting with categorical features. arXiv, arXiv:1706.09516v5 https://arxiv.org/abs/1706.09516.

Pryluk, R., Kfir, Y., Gelbard-Sagiv, H., Fried, I., and Paz, R. (2019). A tradeoff in the neural code across regions and species. Cell *176*, 597–609.e18.

Rauschecker, J.P. (1997). Processing of complex sounds in the auditory cortex of cat, monkey, and man. Acta Otolaryngol. Suppl. *532*, 34–38.

Rauschecker, J.P., and Scott, S.K. (2009). Maps and streams in the auditory cortex: nonhuman primates illuminate human speech processing. Nat. Neurosci. *12*, 718–724.

Rezai, A.R., Lozano, A.M., Crawley, A.P., Joy, M.L., Davis, K.D., Kwan, C.L., Dostrovsky, J.O., Tasker, R.R., and Mikulis, D.J. (1999). Thalamic stimulation and functional magnetic resonance imaging: localization of cortical and subcortical activation with implanted electrodes. Technical note. J. Neurosurg. *90*, 583–590.

Rilling, J.K., Glasser, M.F., Preuss, T.M., Ma, X., Zhao, T., Hu, X., and Behrens, T.E. (2008). The evolution of the arcuate fasciculus revealed with comparative DTI. Nat. Neurosci. *11*, 426–428.

Rilling, J.K., Glasser, M.F., Jbabdi, S., Andersson, J., and Preuss, T.M. (2012). Continuity, divergence, and the evolution of brain language pathways. Front. Evol. Neurosci. *3*, 11.

Rinne, T., Muers, R.S., Salo, E., Slater, H., and Petkov, C.I. (2017). Functional imaging of audio–visual selective attention in monkeys and humans: How do lapses in monkey performance affect cross-species correspondences? Cereb. Cortex *27*, 3471–3484.

Roberts, T.F., Hisey, E., Tanaka, M., Kearney, M.G., Chattree, G., Yang, C.F., Shah, N.M., and Mooney, R. (2017). Identification of a motor-to-auditory pathway important for vocal learning. Nat. Neurosci. *20*, 978–986.

Romanski, L.M., and Goldman-Rakic, P.S. (2002). An auditory domain in primate prefrontal cortex. Nat. Neurosci. *5*, 15–16.

Romanski, L.M., Bates, J.F., and Goldman-Rakic, P.S. (1999a). Auditory belt and parabelt projections to the prefrontal cortex in the rhesus monkey. J. Comp. Neurol. *403*, 141–157.

Romanski, L.M., Tian, B., Fritz, J., Mishkin, M., Goldman-Rakic, P.S., and Rauschecker, J.P. (1999b). Dual streams of auditory afferents target multiple domains in the primate prefrontal cortex. Nat. Neurosci. *2*, 1131–1136.

Saleem, K.S., and Logothetis, N.K. (2012). A Combined MRI and Histology Atlas of the Rhesus Monkey Brain in Stereotaxic Coordinates (Academic).

Schulze, K., Vargha-Khadem, F., and Mishkin, M. (2012). Test of a motor theory of long-term auditory memory. Proc. Natl. Acad. Sci. USA *109*, 7121–7125.

Seth, A.K., Barrett, A.B., and Barnett, L. (2015). Granger causality analysis in neuroscience and neuroimaging. J. Neurosci. *35*, 3293–3297.

Simonyan, K., and Horwitz, B. (2011). Laryngeal motor cortex and control of speech in humans. Neuroscientist *17*, 197–208.

Sinai, A., Crone, N.E., Wied, H.M., Franaszczuk, P.J., Miglioretti, D., and Boatman-Reich, D. (2009). Intracranial mapping of auditory perception: event-related responses and electrocortical stimulation. Clin. Neurophysiol. *120*, 140–149.

Smith, S.M., Jenkinson, M., Woolrich, M.W., Beckmann, C.F., Behrens, T.E., Johansen-Berg, H., Bannister, P.R., De Luca, M., Drobnjak, I., Flitney, D.E., et al. (2004). Advances in functional and structural MR image analysis and implementation as FSL. Neuroimage 23 (*Suppl 1*), S208–S219.

Solo, V. (2016). State-space analysis of Granger-Geweke causality measures with application to fMRI. Neural Comput. *28*, 914–949.

Steinschneider, M., Nourski, K.V., Rhone, A.E., Kawasaki, H., Oya, H., and Howard, M.A., 3rd (2014). Differential activation of human core, non-core and auditory-related cortex during speech categorization tasks as revealed by intracranial recordings. Front. Neurosci. *8*, 240.

Takeyama, H., Matsumoto, R., Usami, K., Nakae, T., Kobayashi, K., Shimotake, A., Kikuchi, T., Yoshida, K., Kunieda, T., Miyamoto, S., et al. (2019). Human entorhinal cortex electrical stimulation evoked short-latency potentials in the broad neocortical regions: Evidence from cortico-cortical evoked potential recordings. Brain Behav. *9*, e01366.

Tehovnik, E.J., Tolias, A.S., Sultan, F., Slocum, W.M., and Logothetis, N.K. (2006). Direct and indirect activation of cortical neurons by electrical microstimulation. J. Neurophysiol. *96*, 512–521.

Thompson, W.H., Nair, R., Oya, H., Esteban, O., Shine, J.M., Petkov, C.I., Poldrack, R.A., Howard, M., and Adolphs, R. (2020). A data resource from concurrent intracranial stimulation and functional MRI of the human brain. Sci. Data *7*, 258.

Tolias, A.S., Sultan, F., Augath, M., Oeltermann, A., Tehovnik, E.J., Schiller, P.H., and Logothetis, N.K. (2005). Mapping cortical activity elicited with electrical microstimulation using FMRI in the macaque. Neuron *48*, 901–911.

Tyszka, J.M., and Pauli, W.M. (2016). In vivo delineation of subdivisions of the human amygdaloid complex in a high-resolution group template. Hum. Brain Mapp. *37*, 3979–3998.

Van Overschee, P., and De Moor, B. (1993). Subspace algorithms for the stochastic identification problem. Automatica *29*, 649–660.

Wilson, B., Kikuchi, Y., Sun, L., Hunter, D., Dick, F., Smith, K., Thiele, A., Griffiths, T.D., Marslen-Wilson, W.D., and Petkov, C.I. (2015). Auditory sequence processing reveals evolutionarily conserved regions of frontal cortex in macaques and humans. Nat. Commun. *6*, 8901.

Wilson, B., Marslen-Wilson, W.D., and Petkov, C.I. (2017). Conserved sequence processing in primate frontal cortex. Trends Neurosci. *40*, 72–82.

Witter, M.P. (1993). Organization of the entorhinal-hippocampal system: a review of current anatomical data. Hippocampus *3*, 33–44.

Woolrich, M.W., Jbabdi, S., Patenaude, B., Chappell, M., Makni, S., Behrens, T., Beckmann, C., Jenkinson, M., and Smith, S.M. (2009). Bayesian analysis of neuroimaging data in FSL. Neuroimage *45* (1, Suppl), S173–S186.

CellPress
OPEN ACCESS

STAR★METHODS

KEY RESOURCES TABLE

REAGENT or RESOURCE	SOURCE	IDENTIFIER
**Experimental models: organisms/strains**		
Rhesus macaque (*Macaca mulatta*)	Centre for Macaques, Porton Down, UK	N/A
Humans (*Homo sapiens*)	University of Iowa, Iowa City, USA	N/A
**Software and algorithms**		
FSL	Analysis Group, FMRIB	https://fsl.fmrib.ox.ac.uk/fsl/fslwiki
Human Connectome Workbench	Human Connectome Workbench	https://www.humanconnectome.org/software/connectome-workbench
FreeSurfer	Laboratory for Computational Neuroimaging	https://surfer.nmr.mgh.harvard.edu/
AFNI: Analysis of Functional NeuroImages	Scientific and Statistical Computing Core, NIMH	https://afni.nimh.nih.gov/
fMRIPrep: Robust Preprocessing Pipeline for fMRI Data	Center for Reproducible Neuroscience	https://fmriprep.org/en/stable/
MATLAB R2017b	Mathworks	https://www.mathworks.com/
R	R Statistical Analysis	https://www.r-project.org/
RStudio	R Studio: Analysis Platform for R	https://rstudio.com/
Catboost Classifier	Yandex	https://catboost.ai/
SPSS 26	IBM	https://www.ibm.com/analytics/spss-statistics-software
CSD toolbox	Jürgen Kayser, Columbia University	https://psychophysiology.cpmc.columbia.edu/Software/CSDtoolbox/
BIDS imaging format	Brain Imaging Data Structure	https://bids.neuroimaging.io/
SPM anatomy toolbox	Statistical Parametric Mapping, neuroanatomy toolbox	https://www.fz-juelich.de/inm/inm-1/EN/Forschung/_docs/SPMAnatomyToolbox/SPMAnatomyToolbox_node.html
Juelich brain atlas	Cytoarchitectonic brain atlas	http://jubrain.fz-juelich.decytoviewer.php
ANT	Advanced normalization tool	http://stnava.github.io/ANTs/
**Experimental hardware**		
Macaque MRI scanner	Bruker BioSpin	Vertical primate-dedicated 4.7 Tesla
Human MRI scanner	Siemens	Skyra 3 Tesla MRI scanner
Microelectrodes (macaques)	MicroProbes	Platinum/iridium parylene-C coated microelectrodes
Microelectrodes (humans)	Ad-Tech Medical Instrument Corp.	Neurosurgical recording electrodes: TG96N-NP03N-000, MS10R-IP10X-000, IG32C-SP10X-0TB, SD12R-SP05X-000, SD08R-SP10X-000, SD06R-SP10X-000, FG23C-Sp10-Z000
Microstimulator (macaques)	World Precision Instruments	DS8000 and DLS 100 isolator
Microstimulator (humans)	AM Systems	Model 2200
Neural recordings and amplifier (macaques)	Tucker-Davis Technologies	TDT System running Synapse
Neural recordings and amplifier (humans)	NeuraLynx	ATLAS system
**Deposited data**		
Experimental data	Data generated by this study in macaques (OSF, PRIME-DE, PRIME-RE repositories) and humans (OSF and OpenNeuro)	https://osf.io/arqp8
		https://fcon_1000.projects.nitrc.org/indi/indiPRIME.html
		https://prime-re.github.io/
		https://osf.io/arqp8
		https://openneuro.org/

RESOURCE AVAILABILITY

Lead contact

The lead contact for the study is Christopher I. Petkov: chris.petkov@ncl.ac.uk

Materials availability

This study did not generate new unique reagents.

Data and code availability

The macaque and human datasets generated during this study are available as follows. For the macaque data: Open Science Framework https://osf.io/arqp8, PRIMatE Data Exchange https://fcon_1000.projects.nitrc.org/indi/indiPRIME.html and PRIMatE Resource Exchange: https://prime-re.github.io/. For the human data: Open Science Framework https://osf.io/arqp8 or Open Neuro https://openneuro.org/. This study did not generate unique code.

EXPERIMENTAL MODEL AND SUBJECT DETAILS

Macaque subjects

All the nonhuman animal work and procedures were approved by the university Animal Welfare and Ethical Review Body and UK Home Office. The work complies with the revised UK Animal Scientific Procedures Act (1986), the US National Institutes of Health Guidelines for the Care and Use of Animals for Experimental Procedures and with the European Directive on the protection of animals used in research (2010/63/EU). We support the principles on reporting animal research stated in the consortium on Animal Research Reporting of *In Vivo* Experiments (ARRIVE). All persons involved in animal handling and procedures were certified and the work was strictly regulated by the UK Home Office.

Two male rhesus macaques (*Macaca mulatta*) from a group-housed colony were scanned awake with combined electrical stimulation and fMRI (M1 and M2, 10 and 12 years old, weighing 12.2 and 12.6 kg, respectively). The pen sizes in the colony range from 130 × 240 cm to 215 × 240 cm. They are 230 cm high, and hatches between neighboring cages are used to increase the space available to the animals. Given the ethical sensitivities involved in studying nonhuman primates and the 3Rs principles (one of which is on the Reduction of animal numbers), our work requires using the fewest monkeys possible. A sample size of two is common in neuroscience work with macaques provided that results are robust with each individual and that the effects generalize beyond one animal. Given that our results from several hundred trials and many scanning runs with each animal are statistically robust and consistent in the overall pattern of effects between the animals (i.e., no significant interactions of monkeys as a factor with the reported patterns of es-fMRI results) there was little ethical justification to test additional monkeys.

Human subjects

Patients with intractable epilepsy requiring chronic invasive monitoring as part of their clinical treatment participated: n = 30; male = 19, female = 10; age range: 13 - 59 years old; median age = 33 years; handedness: right dominant = 22, left = 6, mixed = 2. All experimental procedures were approved by university Institutional Review Board and written informed consent was obtained from all subjects. Depth and subdural surface platinum electrodes (Ad-Tech Medical Instruments) had been implanted for clinical monitoring. The patients' demographic information and treatment outcomes are shown in Tables S3 and S4.

METHOD DETAILS

Macaque es-fMRI procedure

The electrical stimulation procedure used in this work is based on methodology developed in prior macaque es-fMRI studies (Logothetis et al., 2010; Petkov et al., 2015; Tehovnik et al., 2006; Tolias et al., 2005). Here, the procedure was conducted on awake monkeys in absence of alterations due to anesthetics (Ekstrom et al., 2008; Moeller et al., 2008) to allow more direct comparison with awake human es-fMRI data.

Prior to the experiments, an MRI-compatible PEEK head post and chamber were implanted stereotaxically during an aseptic procedure under general anesthesia. The chamber was positioned over the right hemisphere to provide access to posterior auditory cortex, including fMRI tonotopically localized fields A1 and the posterior area adjoining the border between belt fields CL and CM (Figure 2).

During each scanning session, a custom-made PEEK microdrive was used to advance the stimulating electrode to auditory cortex. Monopolar electrical stimulation was induced through platinum/iridium microelectrodes coated in parylene-C (Microprobes, Gaithersburg, Germany), with a typical impedance of 100-200 kΩ (electrodes with impedance below 75 kΩ were not used). The stimulating electrode location in auditory cortex was confirmed via MRI structural scans and sound-evoked neurophysiological responses as in previous studies (Petkov et al., 2015), using a TDT system running Synapse (Tucker-Davis Technologies, Alachua, FL). The

experiment was controlled by MATLAB software (MathWorks, Natick, Massachusetts, US) running the Psychophysics Toolbox, interacting with the hardware connected via a LabJack interface device (LabJack Measurement and Automation). The experimental computer triggered each MRI scan.

The target sites were electrically stimulated using a World Precision Instruments (DS8000) waveform generator with an electrical stimulus isolator unit (DLS 100). The system delivered a constant-current, charge-balanced electrical pulse of either 0.5 or 1 mA (fixed within a stimulation session) at a repetition rate of 200 Hz (within 5 pulse periods; Figure 2A). Each biphasic-square-wave pulse had 0.2 ms duration. The stimulation and MRI scanning timing paradigm are shown in Figure 2B. Electrical stimulation was randomly induced in 50%–70% of the scanning trials with the others containing no stimulation, acting as the baseline point of reference.

Functional MRI measured the blood oxygen level dependent (BOLD) signal, using an actively shielded vertical primate-dedicated 4.7 Tesla MRI scanner (Bruker BioSpin, Ettlingen, Germany). The monkeys had been slowly acclimated over several months with reinforcement training to work in the primate scanning chair and with the required periods of head immobilization during fMRI (Rinne et al., 2017). They were scanned awake under head immobilisation while conducting a visual spot fixation task (Wilson et al., 2015).

A custom 4-channel surface receiver coil array and a saddle transmitter coil were used for MRI acquisition (WK Scientific, California USA). Functional data were obtained using a gradient-recalled echo planar imaging (GE-EPI) sequence, with the following parameters: echo time (TE) = 20 ms; volume acquisition time (TR) = 2000 ms; flip angle = 90 degrees; 32 slices, in plane with a field of view (FOV) of 10.7 × 10.7 cm^2, on a grid of 88 × 88 voxels. Voxel resolution was 1.2 × 1.2 × 1.3 mm^3 covering much of the brain. A sparse fMRI acquisition paradigm was used to minimize the interference caused by the scanner noise on the auditory cortex response (Hall et al., 1999; Petkov et al., 2009); a single T2* weighted MRI volume was acquired after the electrical stimulation or no-stimulation periods for each trial. The onset of the volume lagged by ~4 s to account for the lag in the hemodynamic response (Baumann et al., 2010); namely the stimulation period started 6.4 s prior to the MRI volume onset and lasted for 3 s (5 repeats of the alternating 200 ms stimulation and 400 ms no-stimulation period, see Figure 2B). Each trial lasted approximately 10-13 s. The number of trials obtained for each scanning run and animal were 60 for M1 and 120 for M2. The numbers of scanning runs analyzed were 20 (9 in M1; 11 in M2) for Site 1 stimulation and 17 (8 in M1; 9 in M2) for Site 2, see Figure 2.

Anatomical images, including those for helping to visualize the electrode position, were acquired at the beginning and end of each experimental testing session using magnetization-prepared rapid gradient-echo (MP-RAGE) sequences. Typical sequence parameters were: TE = 20 ms; inversion time, TI = 750 ms; TR = 2000 ms; 50 slices with in-plane field of view: 10.7 × 10.7 cm^2 on a grid of 140 × 140 voxels. The voxel resolution was 0.75 × 0.75 × 0.75 mm^3.

Macaque es-fMRI processing

We performed General Linear Analysis using FEAT in FSL (Smith et al., 2004; Woolrich et al., 2009). We contrasted the BOLD responses during stimulation and non-stimulation trials. Each scanning volume in this sparse imaging paradigm was assigned to either the stimulation or no-stimulation conditions. Brain extraction used the BET function in FSL, and the fMRI image series were motion corrected and realigned within a given testing session using FLIRT (typically 9 or 12 degrees of freedom affine transformation with the normalized mutual information option). The motion parameters were used as regressors of no interest in the fMRI analyses. The data were smoothed with a Gaussian kernel 2 mm Full Width Half Maximum (FWHM). For registration of the fMRI time series, the T2-weighted scans were registered to the animal's session mean functional scan covering the brain. This scan was then registered to the animal's session anatomical and then to its high-resolution anatomical scan. Finally these scans were registered to a macaque template brain (McLaren et al., 2009), which is in register to a macaque atlas in stereotactic coordinates (Saleem and Logothetis, 2012).

Human es-fMRI procedure

Details of the human es-fMRI procedure and safety testing are also available elsewhere (Oya et al., 2017). Briefly, the human subjects in this study were scanned awake and passively. In this study, we focused on data from auditory cortex stimulation with combined fMRI. Pre-electrode implantation T1-weighted and T2-weighted structural MRI images were obtained at 3 Tesla (GE Discovery 750W scanner, 32 channel head coil): T1w inversion recovery fast spoiled gradient recalled (BRAVO) sequence, 1.0 × 1.0 × 0.8 mm^3 voxel size, TE = 3.28 ms, TR = 8.49 ms, TI = 450 ms, FOV = 240 mm^3, flip angle = 12 degrees; T2w: 3-D fast spin-echo (CUBE) sequence, 1.0 × 1.0 × 1.0 mm^3 voxel size, TE = 77.21 ms, TR = 3200 ms, TI = 450 ms, FOV = 256 mm^3, flip angle = 90 degrees.

During es-fMRI scanning sessions, structural T1-weighted images were obtained using a Siemens Skyra 3 Tesla scanner (MPRAGE sequence with 1.0 × 1.0 × 1.0 mm^3 resolution, TE = 3.44 ms, TR = 1970 ms, TI = 1000 ms, Flip angle = 10 deg. , FOV = 250 mm^3). A transmit and receive head coil was used for es-fMRI sessions to obtain both structural and functional scans. Gradient-echo, echo-planar imaging (GE-EPI) was used to obtain the T2* weighted BOLD scans (TR = 3.0 s, TE = 30 ms, slice thickness = 3.0 mm, FOV = 220 mm^3, flip angle = 90 degrees, Phase encoding lines = 68).

Stimulus isolators were used for electrical stimulation, connected to two of the available intracranial electrode contacts. Stimulus waveforms were computer controlled and electrical stimulation was induced via an optically isolated stimulation unit (AM Systems, Model 2200). The control computer received and timed the electrical stimulation via a trigger from the scanner indicating the start of each EPI volume acquisition. Stimulation was bipolar using adjacent contacts (inter-contact distance was 5 or 10 mm) with stimulus intensity between 9-12 mA using constant-current electrical stimulation. In-vivo impedance of the electrode contacts ranged from 1.5 kΩ to 5.5 kΩ at 100 Hz for both depth (cylindrical) and surface (disk) electrode contacts. Electrical stimulus waveforms were

charge-balanced biphasic square waves (0.2 and 0.6 ms duration) with a 0.2 ms inter-stimulation pulse period at 100 Hz repetition as illustrated in Figure 3B. Stimulation was delivered in blocks of 7 or 9 pulses repeated for 10 consecutive TRs followed by a 30 s no-stimulation baseline period. Each scanning run contained 10 stimulation and 11 no-stimulation blocks. Overall, 42 es-fMRI runs in 19 patients were available for this study (Table S3). Stimulated sites in all of the individuals' brains are shown in Figure S2.

Stimulation site categorization procedure

The stimulation sites in the human es-fMRI (and the electrical tractography, see below) were divided into two categories: 1) postero-medial HG sites (medHG); and 2) antero-lateral HG sites which included some sites on the planum temporale (latHG+PT). The number of es-fMRI runs with Site 1 stimulation and Site 2 stimulation were 23 and 19, respectively.

The two sites were categorized according to the electrophysiological responses to click sounds presented at different rates (Brugge et al., 2008). Click trains of various repetition rates (0.2 ms square pulse, 25, 50, 100, 150 and 200 Hz, 50 presentations for each condition) were presented to the subject through earphones (ER4B Etymotic Research) binaurally fitted in custom-made ear-molds. The intracranial neurophysiological signal was recorded with an ATLAS system (NeuraLynx) at a sampling rate of 2 kHz (0.1 - 500 Hz acquisition filter). Raw (wideband) averaged potentials were calculated. The wideband averaged potentials were subsequently bandpass filtered centered at the click repetition rate (Windowed FIR filter with tap length of 250 sampling points, passband width 8 Hz). If averaged evoked potentials in response to click trains showed short-latency (< 20 ms) waveform components and a frequency-following response to the 50 Hz or higher click rate, that contact was categorized as Site 1 (medHG). This is because the typical distribution of these sites are in the posterior to medial part of HG (Brugge et al., 2008). If the click-train induced averaged potentials showed clear wideband auditory evoked potentials but failed to show a strong frequency-following response, that site was categorized as Site 2 (latHG+PT); these types of responses are more typical in higher-order auditory regions including the antero-lateral parts of HG and planum temporale, see Figure S2B.

Electrode localization procedure

The location of the implanted electrodes was determined by comparing pre- and post-electrode implantation structural T1w MRI scans. To compensate for potentially significant displacement of the electrode due to postoperative brain shift, post-implantation volumes were non-linearly warped into pre-implantation MRI volume space using a thin-plate spline (TPS) procedure with manually selected control points for the electrodes in three-dimensional space (Oya et al., 2017, 2018). Between 50 and 100 control points throughout the brain were selected in this step. Contact coordinates in the subject's original space were transformed to standard MNI space using affine transformation and surface-based non-linear transformation implemented in FreeSurfer (Fischl, 2012).

For surface grid electrodes, the locations of all grid contacts were identified in a postoperative CT scan. This was accomplished by manually identifying the location of a subset of contacts in the grid on the basis of the characteristic hyper-intense radiological artifacts. Identified contacts included the depth electrode contacts or the full 64- or 96-grid fitted to these locations by TPS warping, using a negligibly small regularization parameter. Applying TPS allowed the non-linear deformation of the grid to be approximated. Accuracy of fitting was evaluated by visually comparing fitted contact locations with the contact artifacts in the CT and by verifying that inter-contact spacing fell within 0.2 mm of the expected 5-10 mm contact spacing.

After the initial grid locations were determined using CT, these were further corrected using a pre-explantation MR scan. Because displacement of brain parenchyma related to electrode mass-effect and post-operative swelling is often difficult to evaluate accurately on the CT scan, the results of CT-based localization were compared against a T1w MR scan obtained shortly before explantation. When significant discrepancy (greater than approximately 2 mm) was observed between CT-derived contact locations and corresponding magnetic susceptibility artifacts in the MR scan, a rigid linear transform was used to adjust grid positioning on the basis of clearly identifiable electrode-related artifacts in the MRI scan. The corner contacts were used as control points in this transformation. Individual T1w structural volumes were warped onto the CIT168 template brain (registered in MNI space, NeuroVault) with ANTs symmetric normalization algorithm (Cox, 1996; Tyszka and Pauli, 2016) and the contact coordinates in the original space warped onto the CIT168 template yielded the MNI coordinates used in this study.

Human es-fMRI data processing

The anatomical and functional imaging data were pre-processed using the fMRIPrep pipeline (Esteban et al., 2019).

Anatomical image preprocessing

The high resolution T1w image was corrected for intensity non-uniformity using 'N4BiasFieldCorrection' and was used as a reference image throughout the workflow. The T1w-reference was then skull-stripped using 'antsBrainExtraction.sh' (ANTs 2.2.0) with the OASIS template as a target (Cox, 1996). Spatial normalization to the ICBM 152 Nonlinear Asymmetrical template version (2009c) was performed through nonlinear registration with 'antsRegistration', using brain-extracted versions of both the T1w and template brains. Brain tissue segmentation of cerebrospinal fluid (CSF), white-matter (WM) and gray-matter (GM) was performed on the brain-extracted T1w using FAST in FSL.

The subject's pre-electrode implantation structural MRI and the template brain (MNI-152-NonLinear-2009c Asymmetrical brain) were processed with FreeSurfer 'recon-all' procedure to create the surface mesh. For improved pial surface reconstruction, cortical parcellation was facilitated by the T2w structural scans (1.0 × 1.0 × 1.0 mm^3) obtained during the same imaging session whenever

CellPress
OPEN ACCESS

possible. For mapping the BOLD and electrophysiological response onto the brain surface, the FreeSurfer surface meshes were further processed with AFNI's @SUMA_Make_Spec_FS to create standard icosahedron surfaces with various mesh densities.

Functional data preprocessing

For each of the es-fMRI runs per subject, first a reference volume and its skull-stripped version were generated using fMRIPrep. The T2*-weighted reference was then co-registered to the T1w reference using FLIRT in FSL with the boundary-based registration cost-function. Co-registration was configured with nine degrees of freedom to account for T2*w distortions. Head-motion parameters with respect to the BOLD reference (transformation matrices, and the six rotation and translation parameters) were estimated before any spatiotemporal filtering using FLIRT in FSL. EPI scans were slice-time corrected using '3dTshift' from AFNI (Cox, 1996). The BOLD time-series (including slice-timing correction when applied) were resampled onto their original, native space by applying a single, composite transform to correct for head-motion and susceptibility distortions. The BOLD time-series were then resampled to the MNI-152-NonLinear-2009cAsymmetrical standard space. Several confounding time-series were calculated based on the preprocessed BOLD: framewise displacement (FD), DVARS and three region-wise global signals. FD and DVARS are calculated for each functional run, both using the implementation in Nipype @power_fd_dvars.

The three global signals, CSF, WM and whole-brain masks were extracted, though not used as nuisance regressors. Additionally, a set of physiological regressors were extracted to allow for component-based noise correction (CompCor). Principal components were estimated after high-pass filtering the preprocessed BOLD time-series (using a discrete cosine filter with 128 s cut-off). We also defined two CompCor variants: temporal (tCompCor) and anatomical (aCompCor). Six tCompCor components were also calculated from the top 5% voxel variability within a mask covering subcortical regions. This subcortical mask was obtained by heavily eroding the brain mask, which ensured it did not include cortical gray matter. The head-motion estimates calculated in the motion correction step were also added to the confounding variables file. All resampling was then performed using a single interpolation step by composing all the pertinent transformations (i.e., head-motion transform matrices, susceptibility distortion correction, when available, and co-registration information to anatomical and template spaces). Gridded volumetric resampling was performed using 'antsApplyTransforms' (ANTs), configured with Lanczos interpolation to minimize smoothing effects (@lanczos).

The above fMRIPrep processing pipeline generally yielded good registration between anatomical and functional imaging data, even with signal dropout due to the intracranial electrodes. If misalignment was obvious in the visual inspection of EPI to T1w registration or as reported in fMRIPrep, a further registration step was implemented. Here, the functional data was clipped in the sagittal plane discarding parts of the functional data that had significant signal dropout and this volume was used for finding the coregistration parameters using AFNI's 'align_epi_anat.py' program with normalized mutual information as a cost function. During this step, the part of the brain contaminated with the intracranial electrodes was not used for coregistration and this yielded safisfactory coregistration.

Human electrical stimulation tract-tracing (esT)

Electrical stimulation neurophysiological tractography (esT) was conducted in human patients (n = 13, Table S3) according to general methods described previously (Brugge et al., 2003; Garell et al., 2013). Here, we used a single constant current electrical stimulation pulse (biphasic charge-balanced square wave, duration = 0.2 ms/phase, 9 or 12 mA). The electrical pulses were delivered through stimulus isolators connected to the intracranial electrodes in a bipolar configuration (always connected to adjacent contacts). The inter-stimulus interval was set to 2 s and repeated 60 times. The intracranial EEG signal was recorded using the ATLAS system (NeuraLynx) with a sampling frequency of 8 kHz. A disk contact in the subgaleal space was used as a reference electrode for the recordings. Stimulated sites included HG, STG, VLPFC and hippocampal contacts. Average potentials were calculated for each contact after rejecting trials that contained large amplitude non-physiological signals after applying a high-pass filter (4th order Chebyshev type 2, −6 dB roll-off at 3 Hz) and de-meaning the potentials. The trial exclusion criterion for rejection was a signal greater than 3 times the interquartile range above the 75th percentile of the amplitude distribution.

Spline-Laplacian correction

A Laplacian procedure was applied to reduce non-specific neurophysiological or electrical stimulation effects evident as cross-correlated potentials common to many electrodes from a common source or far-field potentials (Nourski et al., 2013; Nunez and Pilgreen, 1991). This procedure spatially corrects the average potentials after spline interpolation. The Laplacian operation is a spatial high-pass filter (second derivative) and the resulting potentials are reference independent and de-emphasize far-field effects or those from volume conduction. For depth electrode potentials, a 1-dimensional spline-Laplacian was calculated using the inter-contact distance information to calculate the spline-Laplacians. To calculate spherical spline-Laplacians (Carvalhaes and de Barros, 2015; Kayser and Tenke, 2015; Perrin et al., 1989). For potentials from surface grids and strip electrodes, we used the spherical coordinates corresponding to each contact's MNI coordinates based on FreeSurfer's spherical surface mapping (Fischl, 2012). The regularization parameter for spherical surface spline-Laplacian computation was determined by generalized cross-validation (Carvalhaes and de Barros, 2015) and the spline-flexibility parameter was set to 3. The magnitude of esT responses was quantified by comparing the root mean square (RMS) values of the Laplacian transformed waveforms between the post-stimulation period (10 - 200 ms after stimulation onset) and pre-stimulation period (−500 to −10 ms) prior to the electrical stimulation pulse. To reduce the effect of response magnitude variability on the across-run average potentials, the individual-run laplacian waveforms were normalized with respect to their RMS values within 10 - 300 ms after the onset (not incuding the stimulus artifacts).

esT movie creation method

To make the movies, continuous intracranial recordings were cut into trials (−1 to 1 s around electrical stimulation). The following procedure that we used yields similar results to one extracting high-frequency power (gamma and high-gamma band), but it avoids the need for temporal band-pass filtering and frequency decomposition. Stimulation artifacts (−5 to 5 ms from stimulation) were first replaced with the time-reversed waveform of the same length (10 ms) during an artifact free pre-stimulus period (−10 to −5 ms). Then a peri-stimulus waveform of 12 ms duration (−6 to 6 ms) was smoothed twice with median filters (length 3 ms, followed by 5 ms). This process was done for all single-trial waveforms. The trials were then re-referenced to the average of the surface grid contacts, to reduce the volume conducted artifact waveforms common across the recording surface grid. To extract induced responses (not phase-locked components), each channel's averaged potential in the re-referenced signal was subtracted from the single trials in that channel (Sinai et al., 2009). Induced response magnitude was calculated by taking the trial average of the full-wave rectified signal.

The magnitude of stimulation-induced responses was calculated by taking the logarithm of the ratio of the magnitude in the pre-stimulus period (0.5 to 0.3 s before stimulus onset) and post-stimulus period. This yields the relative magnitude change with respect to the pre-stimulus period in dB. To make the movies, averaged induced responses of the surface contacts were calculated for non-overlapping temporal windows (length 5 ms), then color-coded and plotted onto the MNI template brain for each experimental run and temporal window. We employed bootstrapping (1000 iterations) of the mean activity within 5 ms temporal windows from the pre-stimulus period (55 to 7.5 ms before stimulation onset) and thresholded the response at the lower and upper 2.5% points. Responses not exceeding this threshold were set to 0 and thus were not mapped to the brain surface. For patients who had multiple esT sessions for either medHG or latHG sites, we averaged responses for each site separately to create the movies (Videos S1 and S2).

Speech sound recording experiment and processing

To examine the brain's effective connectivity under a natural sensory stimulation setting, we examined neurophysiological responses to speech sounds. Many of the subjects were the same neurosurgical patients (n = 8) who took part in the electrical tractography study (Tables S3 and S4).

Speech stimuli

The experiment used a speech presentation paradigm previously described (Nourski et al., 2017; Steinschneider et al., 2014). The speech sounds were common monosyllabic consonant-vowel-consonant English words, e.g., "cat," "dog." All sounds were normalized to the same root mean square amplitude and edited to be 300 ms in duration with 5 ms amplitude rise and fall times. Sounds were delivered binaurally via insert earphones (ER4B, Etymotic Research, Elk Grove Village, IL, USA) integrated into custom-fit ear molds. Sound delivery was controlled using Presentation software (Version 16.5 Neurobehavioral Systems). Altogether 240 presentation trials were presented during two experimental blocks. The subjects were asked to press a button when they heard a target word (only non-target word responses analyzed here) using their index fingers ipsilateral to the hemisphere from which the recordings were made.

Event-related spectral LFP decomposition

Intracranial recording data were downsampled to 1000 Hz. The analysis of the neurophysiological responses focused on five frequency bands: theta (4-8 Hz), alpha (8-14 Hz), beta (14-30 Hz), gamma (30-70 Hz) and high gamma (70-150 Hz) denoised using a demodulated band transform-based algorithm (Kovach and Gander, 2016). Event-related spectral perturbations were calculated by log-transforming the power for each center frequency and normalizing it to the baseline (mean power in the pre-stimulus reference interval of −200 ms to −100 ms before stimulus onset). The waveforms were then averaged across trials.

QUANTIFICATION AND STATISTICAL ANALYSIS

Macaque quantification and statistical analysis

General Linear Modeling

We first preprocessed each scanning run using first-level analyses. Individual scanning runs with little evidence of the expected electrically induced activity (at a liberal uncorrected threshold $Z > 2.3$) in auditory areas around the electrode or in auditory cortex in the opposite hemisphere were not analyzed further. Group higher-level analyses were conducted combining all of the viable scanning runs grouped by stimulation site (Site 1 or 2). Higher-level analyses were conducted using FLAME in FSL with a significance threshold at a cluster corrected ($p < 0.05$, $Z > 2.8$) level. FreeSurfer was used to project the results onto the surface-rendered macaque template brain (Fischl, 2012). Table S1 shows the anatomical regions with significant electrically induced es-fMRI activity (x, y, z in the macaque atlas brain space) resulting from Site 1 or 2 stimulation. The contrast between Site 1 and Site 2 stimulation did not result in any cluster corrected ($p < 0.05$) voxels.

Region of Interest analyses

ROI analyses used anatomically defined regions from the macaque atlas (Saleem and Logothetis, 2012) registered to a macaque template brain (McLaren et al., 2009) and to each animal's dataset. For VLPFC subregion analyses, we used anatomically delineated areas 44 and 45 from the atlas and FOP from prior work (Wilson et al., 2015). The FOP includes anatomical areas PrCO and the dysgranular insula ventral to areas 44 and 45. It excludes area 6v, caudal to area 44, and somatosensory, gustatory and orbital frontal cortex areas.

CellPress
OPEN ACCESS

The MTL subregion analyses used anatomical regions corresponding to the PHG, entorhinal cortex (EC), subiculum (Sub), dentate gyrus (DG) and the CA1, 3 and 4 subregions (CA2 was not used because it is not available in the human brain atlas). No voxels overlapped between ROIs. Polar plots using the ROIs (Figure 5) show the average positive BOLD peak *Z*-value across the scanning runs.

Statistical tests

Mixed-design ANOVA models were used to examine ROI effects. The statistical test was implemented in SPSS 24 (IBM Corp, USA) and used scanning run ROI peak *Z*-values as the dependent variable, with between-subject factors of Monkey (M1, M2) and Species (in the cross-species comparison: Macaque), within-subjects factors of ROI (VLPFC or MTL ROIs), Hemisphere (left or right) and Stimulation site (Site 1 or 2) as covariate. We ensured that the data fit normality and equality of variance assumptions by using rank-based normalization and reporting Greenhouse-Geisser corrected results as required.

Human quantification and statistical analysis

General Linear Modeling

The preprocessed functional datasets were subjected to univariate general linear model (GLM) analysis using AFNI's 3dDeconvolve routine. For the GLM analysis, functional data was spatially smoothed with a Gaussian kernel (FWHM = 6.0 mm). Stimulus times were convolved with a 1-parameter gamma function. Baseline detrending was applied with a Legendre polynomial (5 degrees). Volumes (TRs) that showed large levels of motion (FD > 1.0 mm) across adjacent TRs were discarded. The first six tCompCor components extracted above and the FD time-series were added to the baseline model as nuisance regressors.

A brain mask was created before spatial smoothing using intensity thresholded EPIs excluding areas of signal dropout from the electrodes contributing to the fMRI analyses. The clinically determined seizure onset zone (SOZ) was also excluded from the brain mask. If the SOZ affected any part of an ROI, the result for that run and ROI was excluded from further analysis. We show an incidence map in Figure S2C showing the data across the brain that were included.

For the higher-level group analysis we used AFNI's '3dREMLfit' (Cox, 1996). Datasets showing evidence of a response anywhere within the brain mask (false-discovery rate corrected, Z > 2) were submitted to higher-level analysis (42 datasets out of 53 datasets). Resulting statistical maps were subjected to multi-level mixed-effects analysis using '3dMEMA'. The first- and higher-level GLMs were conducted in standard space (MNI-152-NonLinear-2009c Asymmetrical).

ROI analyses

Regions of interest analyses of the VLPFC and MTL used anatomically defined ROIs from standard anatomical atlases of the human brain. The VLPFC subregion analyses included as ROIs areas 44 and 45, and the frontal operculum (FOP). For area 44 and 45, the parcellation is based on the Jülich histological (cyto- and myelo-architectonic) atlas using a 25% probability threshold (Eickhoff et al., 2005). The human FOP ROI is based on prior work (Wilson et al., 2015), and it includes the frontal operculum areas ventral to areas 44 and 45, excluding somatosensory, gustatory and orbital frontal cortex areas. The MTL subregion analyses used anatomical regions corresponding to the following subregions: subiculum (Sub), dentate gyrus (DG) and the CA1, 3 and 4 subregions, using FreeSurfer's hippocampal subfield segmentation (v6.0) (Iglesias and Sabuncu, 2015). For the entorhinal cortex (EC) and parahippocampal gyrus (PHG), we used FreeSurfer's cortical segmentation (aparc+aseg files) from the Desikan-Killiany atlas. No ROIs had overlapping voxels. Polar plots (Figure 5) show the average positive BOLD peak *Z*-value, with variability across the scanning runs in the humans.

Statistical tests

Mixed-design ANOVA models were used to examine ROI effects. The statistical test used scanning run ROI peak *Z*-values as the dependent variable, with between-subject factors of Human and Species (in the cross-species comparison: Human), within-subjects factors of ROI (VLPFC or MTL ROIs), Hemisphere of activation (left or right) and Stimulation site (Site 1 or 2) and Stimulated hemisphere (left or right) as covariates. We ensured that the data fit normality and equality of variance assumptions of the models or transformed the data to achieve normal distributions and report Greenhouse-Geisser corrected results as required.

Catboost classification analysis

Cross-species and hemispheric es-fMRI ROI tests

Mean Z-scores across the individual runs within all of the ROIs in the monkey and human data were used for the classification analysis. The data were centered and normalized with respect to their mean and standard deviation for both species to remove response magnitude difference between the species and among ROIs. The machine learning algorithm used was Catboost, which is based on gradient boosting on decision trees (Prokhorenkova et al., 2017). We used 200 iterations with an automatic learning rate with trees of depth 4. Its performance was assessed with cross-validation (10-fold, 100 times). The importance of each feature and ROI for the classification was also derived from the model by calculating the change in loss function (log-loss) when removing each feature from the model. We also performed the same analysis using data with shuffled labels (human-versus-monkey, 1000 times). The importance of each feature using the actual dataset was compared with that from the shuffled data and the 95% point was used to assess significance. Mean feature importance for each ROI and their standard errors are reported (Figure S6).

Species classification

Overall mean species classification accuracy over 100 10-fold cross validation runs was 73.7% (SD = 1.6%). We also performed the same analysis using data with shuffled labels (human-versus-monkey, 1000 times). Mean and standard deviation of the accuracy of the shuffled data analysis was 50.1% and 5.3%, see Figure S6A.

Response hemispheric laterality classification

We also conducted the classification of response hemisphere to see whether the classifier could discriminate the hemisphere from which the data was obtained. For this, the classifier was built separately to analyze the human and monkey data. Significance of the variable importance was assessed in the same manner as above. Classification accuracy for human and monkey data was 67.0% and 47.3%, respectively, and significantly differed across species (t test, $p < 10^{-10}$; Figure S6B).

Vocal motor-related analyses

Vocal motor-related ROIs

The macaque cingulate cortex areas (anterior, middle and posterior) and areas 8, 6v, 6d, M1 and SMA were used from the Saleem and Logothetis macaque atlas linked to the template brain (Saleem and Logothetis, 2012). For the human brain, primary motor, anterior, middle and posterior cingulate cortex ROIs were defined using the Jurich-Zilles macro-label parcellation in MNI space (Eickhoff et al., 2005). Human area 6v parcellation was from (Neubert et al., 2014), and area 6d, primary motor cortex and SMA (areas 6ma and 6mp) were from the Julich-Brain cytoarchitectonic atlas (Amunts and Zilles, 2015). Since there has been a discrepancy in defining the human homolog of the frontal eye fields (which in macaques is within area 8), putative frontal eye-fields in humans seem to be at the junction of the precentral sulcus and the superior frontal sulcus (Hutchison et al., 2012; Koyama et al., 2004; Lobel et al., 2001; Petit and Pouget, 2019). Therefore, for this region in humans, we used a probabilistic inferior frontal junction parcellation from the SPM anatomy toolbox (Eickhoff et al., 2005). For this reason, there is unavoidable overlap between parts of the human area 6v and the area 8 ROIs. For the ROIs provided with probability maps, the threshold was set to 0.25.

State-space Conditional Granger Causality (CGC) analysis

CGC was used to investigate the directional influence between brain regions during speech processing. The method is multivariate and conditional, in the sense that simultaneous time series from a collection of electrodes are included in order to account for both direct and indirect influences between contacts. Intracranial recordings were downsampled to 100 Hz and sectioned into trials from −200 to 1000 ms relative to sound onset. Intuitively, CGC tests if activity in a source area can be used to predict subsequent activity on a target area. We estimated spectral CGC in 500 ms sliding windows in steps of 50ms to construct trial-averaged time-frequency CGC representations between selected pairs of electrodes (Jenison, 2014). Prior to time-frequency CGC analysis, the mean at individual time points across trials was subtracted from the single trial responses and then scaled by the standard deviation.

Several significant problems arise in applying standard Vector Auto-Regressive (VAR) based CGC models to intracranial recordings, which are related to downsampling and nonstationarities (Seth et al., 2015). Most of these recorded time-series contain a moving-average (MA) component that may not be adequately modeled using VAR due to the intractably large model order necessary to handle the MA component. It has been shown that spectral CGC estimates can be obtained with high computational reliability using estimation approaches based on single model-fits. The state-space model addresses a number of theoretical and practical problems related to spectral CGC estimation, e.g., see (Barnett et al., 2018). Spectral CGC was directly computed using Geweke's formulations based on the estimated state-space innovations covariance matrix (Geweke, 1982; Geweke, 1984), cross-spectral densities and transfer functions (Barnett and Seth, 2015; Solo, 2016).

State-space models use state variables to describe a system by a set of first-order differential or difference equations, rather than by one or more VAR *n*th-order differential or difference equations. State variables can be reconstructed from the measured recordings but are not themselves measured during an experiment. For modeling directional influence in the brain, it is possible to directly express the interactions between different regional signal time series as a state-space model defined by:

$$\vec{x}(t+1) = A\,\vec{x}(t) + K\,\vec{\varepsilon}(t) \quad \text{state transition equation} \qquad \text{(Equation 1)}$$

$$\vec{y}(t) = C\,\vec{x}(t) + \vec{\varepsilon}(t) \quad \text{observation equation} \qquad \text{(Equation 2)}$$

where $\vec{x}(t)$is an unobserved (latent) *m*-dimensional state vector, and $\vec{\varepsilon}(t)$ is the vector of the innovations or prediction errors. The observed vector of time-series $\vec{y}(t)$ corresponds to the recordings from regions in the targeted network. The state transition matrix *A*, observation matrix *C* and the steady-state Kalman gain matrix *K* are estimated using a subspace method (Van Overschee and De Moor, 1993). Subspace methods are optimal for state-space model parameter estimation, especially for high-order multivariable systems (Becker et al., 2018). The order of the state-space model was 25, which corresponds to the vector size of $\vec{x}(t)$.

To statistically evaluate the reliability of the connectivity results we used a phase-randomization surrogate data technique to construct a null distribution (Lancaster et al., 2018; Prichard and Theiler, 1994). This method consists of randomly shuffling the Fourier phases of each of the intracranial recordings, which generates uncorrelated data with preserved autocorrelation properties. For each original dataset, 500 surrogates were generated, and CGC values that exceeded the 95% threshold were unmasked in the time-frequency representations.

Neuron, Volume *109*

Supplemental information

Common fronto-temporal effective connectivity in humans and monkeys

Francesca Rocchi, Hiroyuki Oya, Fabien Balezeau, Alexander J. Billig, Zsuzsanna Kocsis, Rick L. Jenison, Kirill V. Nourski, Christopher K. Kovach, Mitchell Steinschneider, Yukiko Kikuchi, Ariane E. Rhone, Brian J. Dlouhy, Hiroto Kawasaki, Ralph Adolphs, Jeremy D.W. Greenlee, Timothy D. Griffiths, Matthew A. Howard III, and Christopher I. Petkov

Table S2. Human esfMRI Results, Related to Figure 3. Shown are the human brain regions in reference to the human atlas in MNI x,y,z coordinates, the peak voxel value in the region and the number of voxels above the corrected significance threshold (cluster-wise alpha < 0.05 with primary *p* < 0.005).

Site 1 (medHG) esfMRI human group results:

			**MNI coordinates (mm)**		
**size (voxel)**	**peak T**	**x**	**y**	**z**	Brain structure
831	7.38	54.5	0.5	-28.5	lt. middle temporal gyrus
327	7.10	66.5	56.5	13.5	lt. superior temporal gyrus
291	5.77	-41.5	4.5	-12.5	rt. insula
241	5.62	-65.5	26.5	27.5	rt. supramarginal gyrus
166	4.97	42.5	-29.5	1.5	lt. inferior frontal gyrus, including frontal operculum and orbitofrontal cortex
149	6.54	2.5	26.5	-4.5	lt. thalamus
123	4.57	-32.5	-18.5	-2.5	rt. putamen
84	5.26	32.5	-47.5	21.5	lt. middle frontal gyrus

Site 2 (latHG + PT) esfMRI human group results:

			**MNI coordinates (mm)**		
**size (voxel)**	**peak T**	**x**	**y**	**z**	Brain structure
696	7.29	-23.5	-5.5	-26.5	rt. parahippocampal gyrus, including hippocampus and amygdala
600	7.77	46.5	2.5	-30.5	lt. inferior temporal gyrus
377	7.19	-63.5	10.5	37.5	rt. postcentral gyrus
173	7.34	58.5	-11.5	-6.5	lt. superior temporal gyrus
140	7.58	-31.5	64.5	65.5	rt. superior parietal lobule
104	4.68	-15.5	21.5	-17.5	lt. superior orbital gyrus

Site 1 versus Site 2 esfMRI human group results:

			**MNI coordinates (mm)**		
**size (voxel)**	**peak T**	**x**	**y**	**z**	Brain structure
430	7.21	66.5	56.5	13.5	lt. middle and superior temporal gyrus
196	7.17	-63.5	28.5	25.5	rt. supramarginal gyrus
106	5.39	48.5	-15.5	1.5	lt. inferior frontal gyrus, including frontal operculum and orbitofrontal cortex

Site 2 versus Site 1 esfMRI human group results:

size (voxel)	peak T	MNI coordinates (mm) x	y	z	Brain structure
971	5.65	-35.5	-19.5	-28.5	rt. ATL (incl. entorhinal cortex, parahippocampal gyrus, temporal pole & amygdala)
283	5.60	22.5	-5.5	-28.5	lt. ATL (incl. entorhinal cortex & amygdala)
262	5.71	-63.5	6.5	21.5	lt. precentral gyrus

Table S3. Human Patient Demographics, Related to Figure 3

Patient ID	Age	Sex	Handedness	Language dominance (Wada test)	esfMRI	esT	Speech
292	50	F	L	L	Yes (Y)		
302	47	F	R	**	Y		
307	30	M	R	L	Y		
314	30	F	R	L	Y		
316	31	F	R	**	Y		
320	50	F	R	**	Y		
330	43	M	L	**	Y		
331	35	M	R	**	Y		
334	39	M	L	L	Y		
335	31	M	R	L	Y		
339	45	M	R	**	Y		
352	31	M	Mixed	**	Y		
357	36	M	R	L	Y		Y
369	30	M	R	L	Y	Y	Y
372	34	M	R	L	Y	Y	Y
376	48	F	R	L	Y	Y	Y
384	37	M	R	L		Y	
394	23	M	L	L		Y	Y
395	13	M	R	**	Y		
399	22	F	R	L	Y	Y	Y
400	59	M	L	L	Y		
403	56	F	R	L	Y		
405	19	M	R	L	Y	Y	Y
413	22	M	L	R	Y	Y	
418	25	F	R	L		Y	
423	49	M	R	L		Y	Y
427	17	M	Mixed	**		Y	
429	32	F	R	**		Y	Y
457	18	M	R	**			Y
460	52	M	R	L (fMRI)		Y	

**(not done)

Table S4. Human Patient Clinical Observations and Surgical Resection Sites, Related to Figure 3

Patient ID	Clinical MRI finding	Surgery	Seizure onset zone
292	Left frontal focal encephalomalacia	left ATL+ left frontal seizure focus resection	Left mesial temporal lobe + left frontal lobe
302	Left MTS	Left ATL	Left mesial temporal lobe
307	Left insular cavernoma	Cavernoma resection	Left insula
314	Left occipital cortical dysplasia	No resection	Bilateral mesial temporal lobe
316	Right MTS	Right ATL	Right mesial temporal lobe
320	Right MTS	Right ATL	Right hippocampus
330	Right occipital cortical dysplasia	Right occipital lesionectomy	Left occipital lobe
331	Right MTS	No resection	Left mesial temporal lobe
334	Right temporal ganglioglioma	Right ATL	Right temporal pole, ventral surface of temporal lobe
335	Left MTS	No resection	Bilateral medial temporal lobe
339	Normal	No resection	Not determined
352	Left frontal cystic legion	Left frontal regionectomy	Left frontal cystic mass
357	Normal	Left ATL	Left mesial temporal lobe
369	Right basal ganglia venous anomaly	Right ATL	Right mesial temporal lobe
372	Normal	Left ATL	Left temporal pole
376	Normal	Right ATL	Right mesial temporal lobe
384	Normal	Right ATL, Right frontal pole resection	Right mesial temporal lobe, Right frontal pole
394	Right temporal lobe cavernoma	Right ATL	Right amygdala
395	Left frontal lobe cavernoma	Left mesial frontal lobe resection including left frontal cavernoma	Left frontal lobe
399	Normal	Right ATL+ right ventral frontal seizure focus resection	Right mesial temporal lobe + possible right basal frontal lobe
400	Left MTS	Left ATL	Left mesial temporal lobe
403	Normal	Left ATL	Left mesial temporal lobe
405	Left frontal encephalomalacia	Left frontal regionectomy	Left frontal encephalomalacia
413	Normal	Right ATL	Right mesial temporal lobe
418	Right inferior temporal dysplasia	Right ATL	Right mesial temporal lobe
423	Normal	Left ATL	Left mesial temporal lobe
427	Right frontal encephalomalacia	Right frontal lobectomy	Right frontal lobe
429	Normal	Left ATL	Left mesial temporal lobe
457	Normal	Left ATL	Left mesial temporal lobe
460	Normal	Left ATL	Left mesial temporal lobe

MTS: Mesial temporal sclerosis ATL: Anterior temporal lobectomy

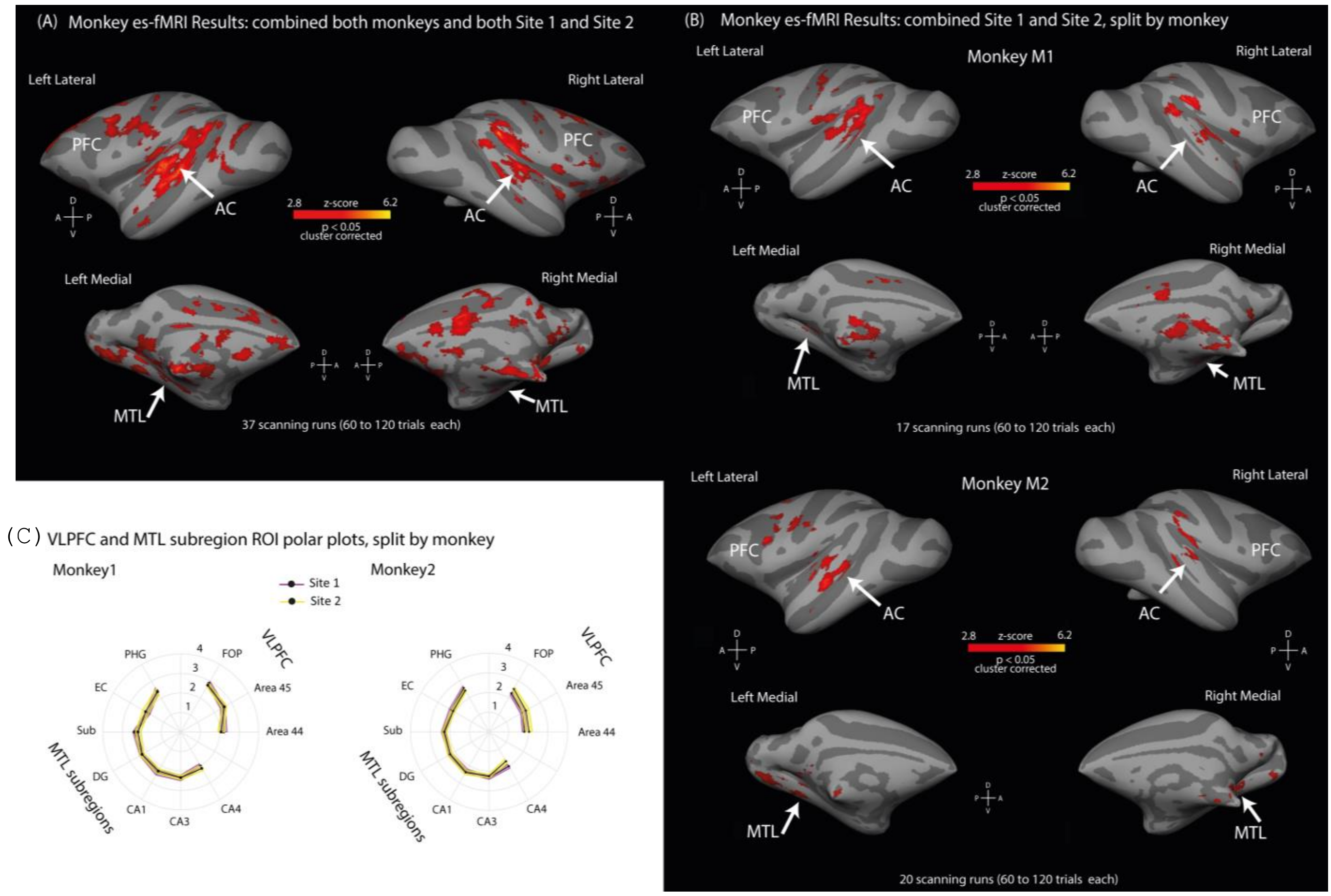

Supplementary Figure S1. Supplementary Monkey es-fMRI Whole Brain and ROI Results, Related to Figure 3. (A) Group es-fMRI result with both monkeys and both sites combined. Format and labels as in manuscript Fig. 2. (B) Group es-fMRI result combining both stimulation sites but shown split by monkey (M1 and M2). Format and labels as in manuscript Fig. 2. (C) ROI polar plot of the max *Z*-score (error boundaries: +/-SEM) across sessions/runs and participants for the VLPFC and MTL shown split by monkey (M1 and M2). Format and labels as in manuscript Fig. 5.

(A)

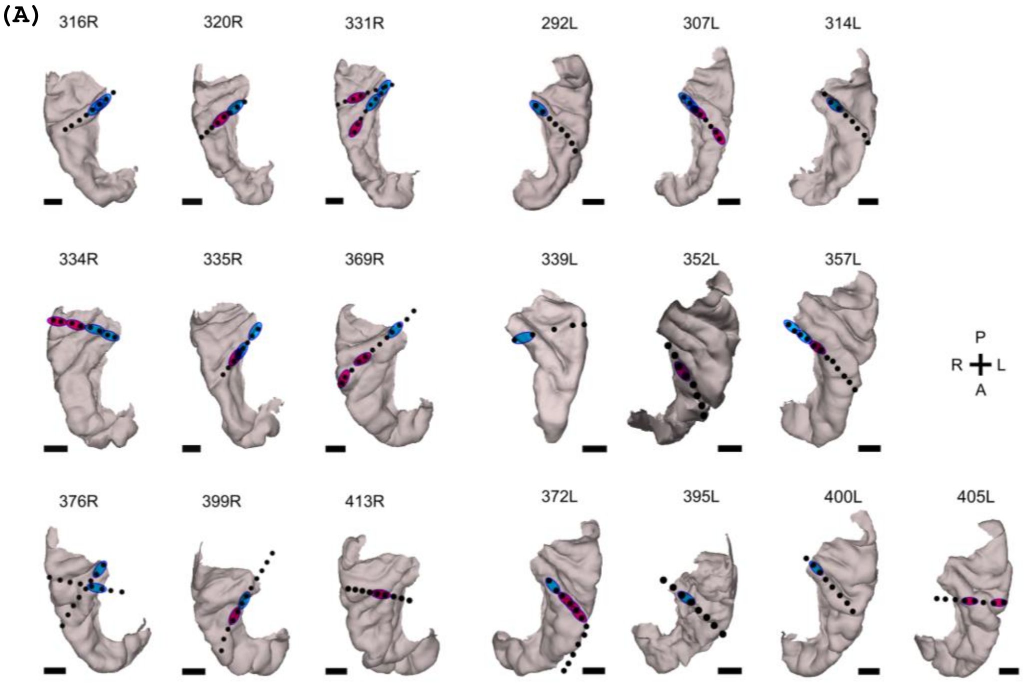

316R
320R
331R
292L
307L
314L
334R
335R
369R
339L
352L
357L
376R
399R
413R
372L
395L
400L
405L
P
R
L
A

(B)

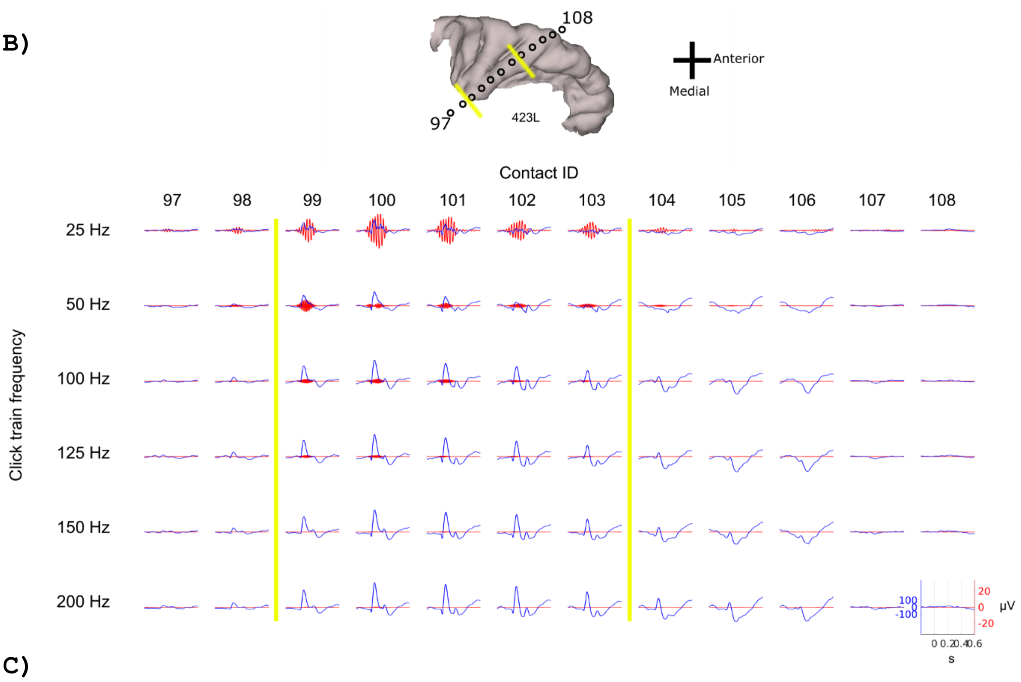

108
97
423L
Anterior
Medial
Contact ID
97
98
99
100
101
102
103
104
105
106
107
108
25 Hz
50 Hz
100 Hz
125 Hz
150 Hz
200 Hz
Click train frequency
100
-100
20
0
-20
µV
s

(C)

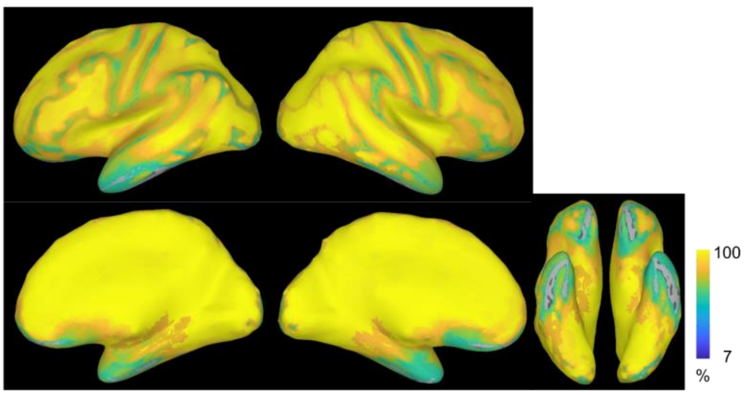

100
7
%

Supplementary Figure S2. Individual Human Depth Electrode Contact Locations for es-fMRI, Click Frequency Following Response Used to Identify medHG Sites, and Electrode Contact Locations Affecting fMRI Signal: Preserved fMRI Signal Map, Related to Figure 3.

(A) Shown are views looking down on the superior temporal plane. Black circles identify the clinically placed contacts and the blue/red regions the two contacts used for bipolar stimulation, respectively in the medHG or latHG+PT sites, shown on each individual's anatomy. Whenever more than two contacts are shown in red is an indication of other pairs of adjacent contacts that were stimulated in a separate testing run. Scale bars 10 mm. L = left; R = right; P = posterior; A = anterior.

(B) Click frequency following response used to identify Site 1 (medHG) sites in humans. Shown is how the click evoked neurophysiological response was used to identify medial HG contacts by their click-frequency following response. In this example, traces in blue show the auditory evoked potentials. Traces in red are the high-pass filtered frequency following response. Contacts between the two yellow lines show a strong frequency following response to 25-100Hz and were thus assigned to medHG. Contacts on the right are assigned to Site 2 (latHG+PT) because they respond to the sounds but do not show a clear high frequency following response. The non-responsive contacts at the flanks of these were not used.

(C) Human electrode contact locations affecting fMRI signal: preserved fMRI signal map. Regions where the fMRI signal was contaminated by the electrodes were masked and excluded from analysis. We also excluded regions that were resected, typically in the anterior medial temporal lobe (Suppl. Table S4). Here is show an incidence map identifying the regions most affected by signal drop out (blue and green colored regions), which includes areas where the fMRI signal is also affected by sinuses (orbitofrontal cortex). Orange to yellow color shows the percentage of human es-fMRI runs that were available for analysis (not masked by intracranial electrodes, weak signal or outside of the epileptic foci regions).

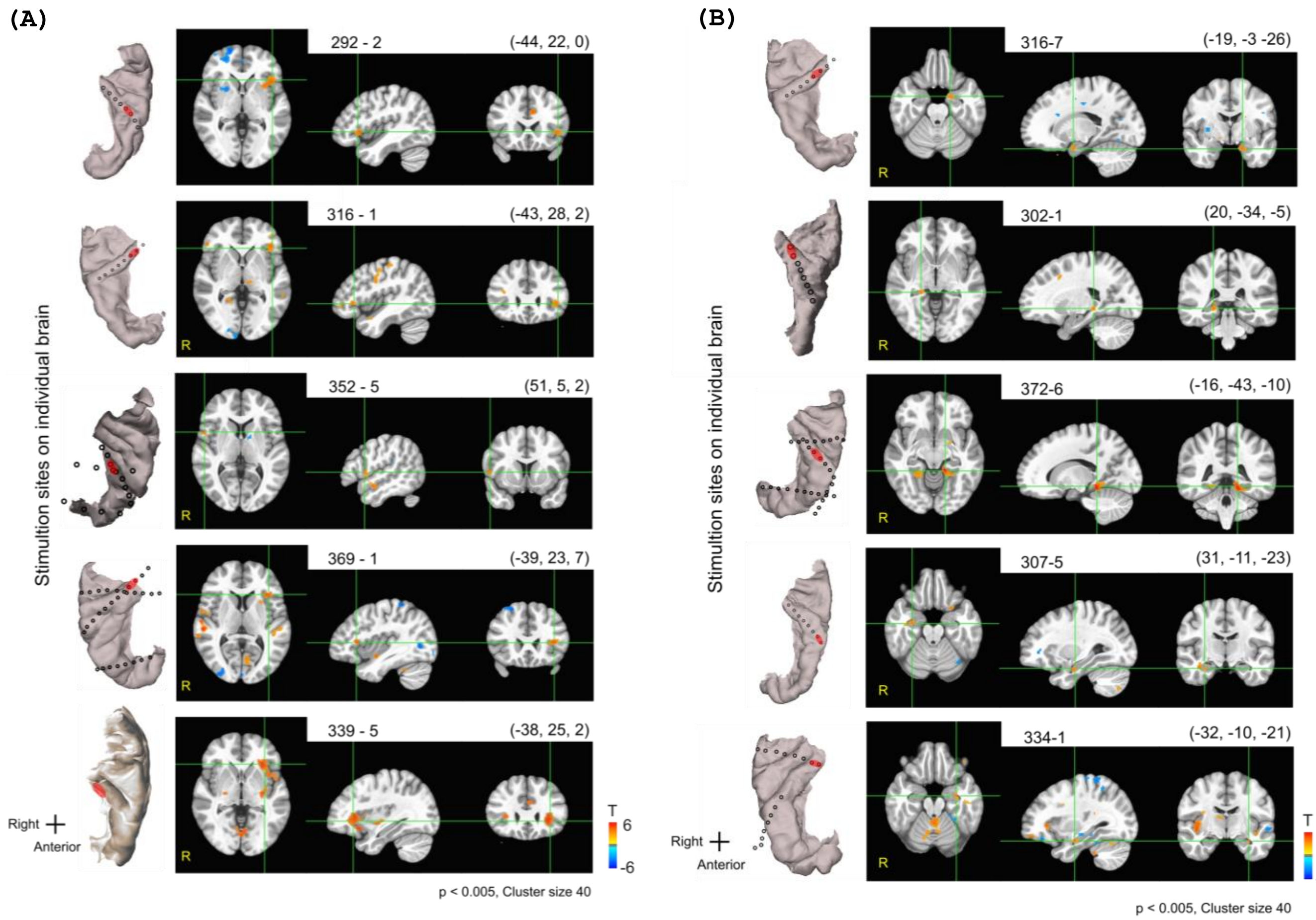

Supplementary Figure S3. Individual Human es-fMRI Activation Results Involving VLPFC and MTL, Related to Figure 3. (A) Shown are several individual human results showing substantial VLPFC (including inferior frontal gyrus) activation $p < 0.005$, cluster size = 40. Numbers above the panels indicate the subject number and testing run, followed by MNI x, y, z coordinates at the cross-hairs. The color bar shows the positive and/or negatively activated BOLD responses as T-values (range -6 to 6). (B) Individual human es-fMRI results involving MTL. Shown are several individual human results showing substantial MTL activation involving hippocampal or parahippocampal areas, $p < 0.005$, cluster size = 40. The color bar shows the positive and/or negatively activated BOLD responses as T-values (range -6 to 6).

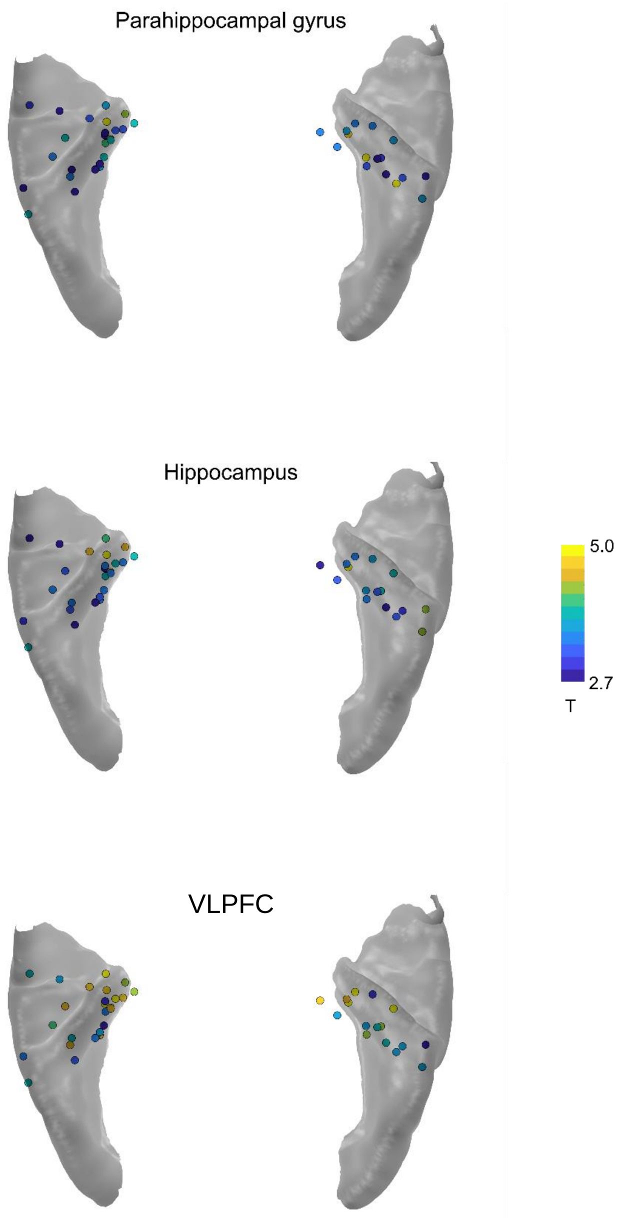

Supplementary Figure S4. Impact of Stimulation Sites on the MTL and VLPFC Contact Sites, Related to Figure 3. Shown are the auditory cortex regions that when stimulated resulted in the displayed strength of fMRI responses on VLPFC and MTL regions. Auditory cortex (Heschl's gyrus) stimulation sites are color coded from blue to yellow color map according to the strength of the fMRI BOLD response elicited in the parahippocampal, hippocampal, or VLPFC (IFG) regions. Although stronger responses tend to be seen from the more medial HG contacts, there are also contacts bordering other regions that also elicit strong responses and passive current spread is a factor, see manuscript text discussion. The color bar shows the positive and/or negatively activated BOLD responses as T-values (range -2.7 to 5).

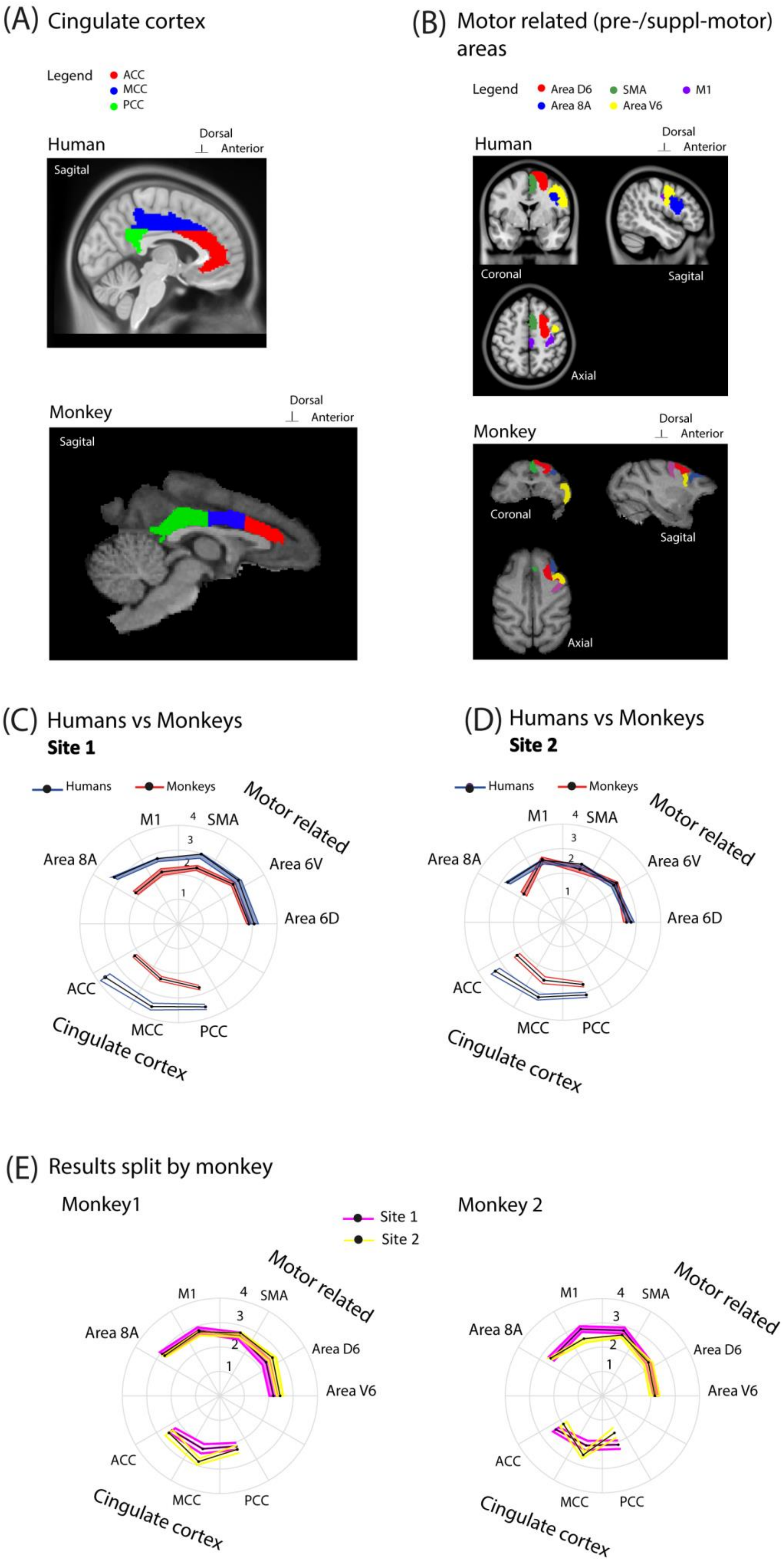

(A) Cingulate cortex
Legend
ACC
MCC
PCC
Human
Dorsal
Anterior
Sagital
Monkey
Dorsal
Anterior
Sagital
(B) Motor related (pre-/suppl-motor) areas
Legend
Area D6
Area 8A
SMA
Area V6
M1
Human
Dorsal
Anterior
Coronal
Sagital
Axial
Monkey
Dorsal
Anterior
Coronal
Sagital
Axial
(C) Humans vs Monkeys
Site 1
Humans
Monkeys
Motor related
M1
SMA
Area 8A
Area 6V
Area 6D
ACC
MCC
PCC
Cingulate cortex
(D) Humans vs Monkeys
Site 2
Humans
Monkeys
Motor related
M1
SMA
Area 8A
Area 6V
Area 6D
ACC
MCC
PCC
Cingulate cortex
(E) Results split by monkey
Monkey1
Monkey 2
Site 1
Site 2
Motor related
M1
SMA
Area 8A
Area D6
Area V6
ACC
MCC
PCC
Cingulate cortex

Supplementary Figure S5. Vocal Production Associated (Motor Related) and Cingulate Cortex Areas: Human and Macaque ROI results, Related to Figure 5. (A) ROIs in cingulate cortex (ACC: anterior cingulate cortex; MCC: middle cingulate cortex; PCC: posterior cingulate cortex). (B) ROIs in motor and pre-/supplementary- motor areas, including vocal motor-production associated areas; Abbreviation: SMA, supplementary motor area. (C-D) human and monkey polar plots split by Site 1 and Site 2 stimulation showing a summary of the max *Z*-score (error boundaries: +/-SEM) across sessions/runs and participants. Format as in manuscript Figure 5. (E) Results split by monkey (M1 and M2).

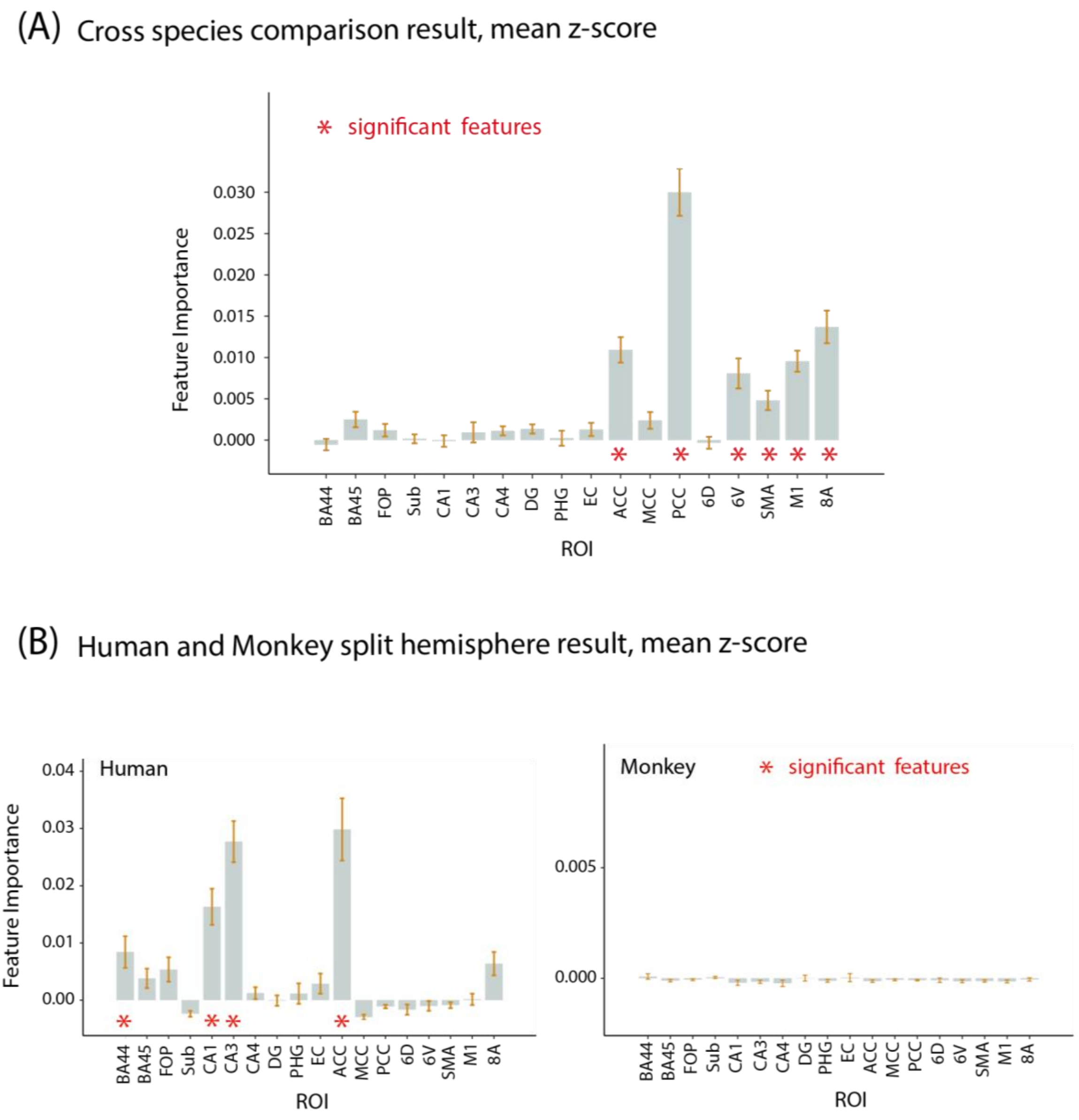

Supplementary Figure S6. Classifier Decoding Results: Species and Hemisphere, Related to Figure 5. (A) Species classification: Variable importance values from the Catboost classifier (see Methods). The larger fMRI response importance values in ACC, PCC, 6V, SMA, M1 and 8A are associated with the model predicting that the data points come from humans rather than monkeys. Red asterisks show significantly larger variable importance. Note how the classifier can only detect the differences in the data from the Cingulate and Vocal motor-related ROIs (right of and including the ACC) but is agnostic to the species difference in the VLPFC (areas 44, 45 and FOP) and MTL regions (Sub, CA1, CA3, CA4, DG, PHG and EC), see manuscript Discussion. (B) Response hemispheric laterality classification: Comparison of accuracy of hemispheric laterality classification, separately for the humans (left panel) and monkeys (right panel). This shows that the classifier can significantly attribute the hemisphere (positive feature importance is for the left hemisphere) in the es-fMRI response in human BA44, CA1, CA3 and ACC. The monkey data in the right panel show very small feature importance values, with the classifier not able to distinguish data from the two hemispheres in any of the regions. For both (A-B) 100 classification models were evaluated with different permutations. Accuracy is determined by 10-fold cross-validation (see Methods).
